# Key interplay between the co-opted sorting nexin-BAR proteins and PI3P phosphoinositide in the formation of the tombusvirus replicase

**DOI:** 10.1371/journal.ppat.1009120

**Published:** 2020-12-28

**Authors:** Zhike Feng, Nikolay Kovalev, Peter D. Nagy

**Affiliations:** Department of Plant Pathology, University of Kentucky, Lexington, Kentucky, United States of America; Agriculture and Agri-Food Canada, CANADA

## Abstract

Positive-strand RNA viruses replicate in host cells by forming large viral replication organelles, which harbor numerous membrane-bound viral replicase complexes (VRCs). In spite of its essential role in viral replication, the biogenesis of the VRCs is not fully understood. The authors identified critical roles of cellular membrane-shaping proteins and PI(3)P (phosphatidylinositol 3-phosphate) phosphoinositide, a minor lipid with key functions in endosomal vesicle trafficking and autophagosome biogenesis, in VRC formation for *tomato bushy stunt virus* (TBSV). The authors show that TBSV co-opts the endosomal SNX-BAR (sorting nexin with Bin/Amphiphysin/Rvs- BAR domain) proteins, which bind to PI(3)P and have membrane-reshaping function during retromer tubular vesicle formation, directly into the VRCs to boost progeny viral RNA synthesis. We find that the viral replication protein-guided recruitment and pro-viral function of the SNX-BAR proteins depends on enrichment of PI(3)P at the site of viral replication. Depletion of SNX-BAR proteins or PI(3)P renders the viral double-stranded (ds)RNA replication intermediate RNAi-sensitive within the VRCs in the surrogate host yeast and *in planta* and ribonuclease-sensitive in cell-free replicase reconstitution assays in yeast cell extracts or giant unilamellar vesicles (GUVs). Based on our results, we propose that PI(3)P and the co-opted SNX-BAR proteins are coordinately exploited by tombusviruses to promote VRC formation and to play structural roles and stabilize the VRCs during viral replication. Altogether, the interplay between the co-opted SNX-BAR membrane-shaping proteins, PI(3)P and the viral replication proteins leads to stable VRCs, which provide the essential protection of the viral RNAs against the host antiviral responses.

## Author summary

Positive-stranded RNA viruses are major pathogens of plants, humans and animals. These viruses hijack and deform intracellular membranes to build viral replication compartments, which support virus replication. In this paper, the authors have identified the critical roles of cellular membrane-shaping proteins and a unique lipid in the formation of the replicase complex for tomato bushy stunt virus (TBSV). TBSV co-opts and retargets the endosomal sorting nexin-BAR proteins into the large TBSV-induced replication compartment. TBSV also promotes the enrichment of PI(3)P phosphoinositide within the subverted membranes. The interplay between the membrane-shaping proteins, PI(3)P and the viral replication proteins leads to stable membranous structures around the viral replicase, which provide the essential protection of the viral RNAs against the host antiviral responses.

## Introduction

RNA virus replication depends on the formation of large intracellular viral replication compartments or organelles (VROs), which represent the sites of intensive viral RNA replication. Biogenesis of VROs for positive-strand (+)RNA viruses requires major membrane remodeling and proliferation, retargeting of trafficking vesicles, and recruitment of numerous host proteins. [1–5]. The functions of VROs include sequestering and concentrating viral components and host factors to support efficient viral RNA synthesis. The virus-induced VROs harbor numerous viral replicase complexes (VRCs). In case of (+)RNA viruses, VRCs are membranous structures, which perform viral RNA synthesis. In addition, VRCs are expected to provide protection of the viral RNAs from recognition by host antiviral sensors and from destruction by host ribonucleases.

Many (+)RNA viruses induce the formation of numerous small spherules, which are vesicle-like structures with narrow opening towards the cytosol [6–8]. The spherules represent single structural units of viral replication and they harbor the VRCs, which replicate viral RNAs, thus critical for virus replication. In spite of major advances in our understanding of spherule formation, the mechanistic and structural insights are still incomplete.

Among plant viruses, tombusviruses are intensively studied to unravel host-virus interactions [9,10]. Tombusviruses have one component (+)RNA genome of ~4.8 kb [11]. They are members of the large Flavivirus-like supergroup that includes important human, animal and plant pathogens. Among the five tombusvirus proteins, only p33 and p92^pol^ are essential for viral replication. p92^pol^ is the RdRp protein and translated from the genomic gRNA via readthrough of the translational stop codon in p33 ORF. The auxiliary p33 replication protein is an RNA chaperone involved in recruitment of the viral (+)RNA for replication and is the master regulator of the VRC assembly process [11–13]. The TBSV replicon (rep)RNA, which contains four non-contiguous segments from the gRNA, can replicate efficiently in yeast and plant cells expressing p33 and p92^pol^ [14].

Intriguingly, tombusviruses take advantage of various cellular compartments for the biogenesis of large VROs, including the formation and assembly of spherule-like VRCs [15]. Tomato bushy stunt virus (TBSV) and the closely related cucumber necrosis virus (CNV) use peroxisomal membranes, whereas carnation Italian ringspot virus (CIRV) exploits the outer membranes of mitochondria. The ER also contributes to VRO formation [16–18] and ER membranes even support TBSV replication efficiently in the absence of peroxisomes [19]. TBSV, similar to other (+)RNA viruses, induces major metabolic and structural changes in the infected cells, including aggregation of peroxisomal and ER membranes, membrane deformations by forming hundreds of 40–70 nm spherules that harbor the VRCs [6,20,21]. TBSV co-opts a large number of host proteins to support various viral functions, including the biogenesis of VROs and the formation of VRCs [10]. VRC formation requires the subversion of several components of the endosomal sorting complex required for transport (ESCRT) machinery of the host cells [21,22]. The TBSV-driven recruitment of the ESCRT components facilitates the invagination of membranes into the peroxisomes, thus promoting spherule formation. TBSV also hijacks lipid resources, leading to enrichment of sterols at the viral replication sites via stabilizing membrane contact sites [23]. TBSV also retargets phosphatidylethanolamine (PE) to the replication sites [24] and recruit Vps34 PI3P kinase (PI3K) to produce PI(3)P (phosphatidylinositol 3-phosphate) within VROs [25]. In spite of the significance of PI(3)P in TBSV replication, the actual role and function of PI(3)P in VRO biogenesis is not known. In addition, we still do not know if PI(3)P might be involved in recruitment of additional host components to facilitate the formation of functional TBSV VRCs.

PI(3)P is a critical signaling and a minor structural lipid molecule, which is a key player in endosomal vesicle trafficking by conferring identity to endosomes [26]. Moreover, PI(3)P plays a crucial role in regulating vesicle fusion and autophagosome formation through its protein effectors. Many intracellular microbes and parasites exploit the cellular PI(3)P to establish infections [27], thus highlighting the central role of PI(3)P in microbe-host intracellular interactions. Because TBSV hijacks the early endosomal compartment and it requires Vps34 PI3K and the production of PI(3)P phosphoinositide within VROs [24,25,28], we have tested if 14 cellular effectors of PI(3)P could affect tombusvirus replication.

In this work, we show key evidence that a family of the PI(3)P effectors, namely the endosomal SNX-BAR (sorting nexin containing Bin/Amphiphysin/Rvs domain) proteins are recruited by TBSV and this is required for complete VRC formation. SNX-BAR proteins, such as Vps5p in yeast and SNX1 and SNX2a/b in plants and SNX1 in mammals, are recruited to specific subdomains of endosomes through binding to PI(3)P via their Phox-homology (PX) domain and sensing positive membrane-curvature through their banana-shaped BAR domains [29,30]. SNX-BAR proteins remodel endosome membranes into tubules, via sensing local membrane curvature and induction of oligomerization [29,31]. SNX-BAR proteins are in the forefront of cellular research due to their central roles in assembly of the retromer cargo-recycling complex [29,32,33]. SNXs are also involved in neurodegenerative diseases including Alzheimer's Disease, Parkinson's Disease and Frontotemporal Lobar Degeneration [34]. SNX proteins are involved in the interactions between hosts and viruses. For example, Cytomegalovirus utilizes SNX5 to regulate the localization of the viral glycoprotein [35]. SNX17 is involved in the cell entry of Papillomavirus [36]. SNX8 is a critical component of the host innate immune response to the herpes simplex virus 1 [37].

We demonstrate that the yeast Vps5p SNX-BAR protein is required for TBSV replication in yeast. The p33 replication protein of TBSV re-localizes Vps5p into VROs as a permanent component of the viral replicase complex. The PI(3)P binding of Vps5p is required for its recruitment and pro-viral function in yeast. Depletion of SNX-BAR proteins renders the viral double-stranded (ds)RNA replication intermediate ribonuclease-sensitive within the VRCs in yeast and plants and also *in vitro* in replicase reconstitution assays. Based on our results, we propose that PI(3)P and the co-opted SNX-BAR proteins are coordinately exploited by tombusviruses to stabilize the VRCs during viral replication.

## Results

### The endosomal sorting nexin-BAR proteins are required for tombusvirus replication in yeast and plant cells

**Fig 1:**
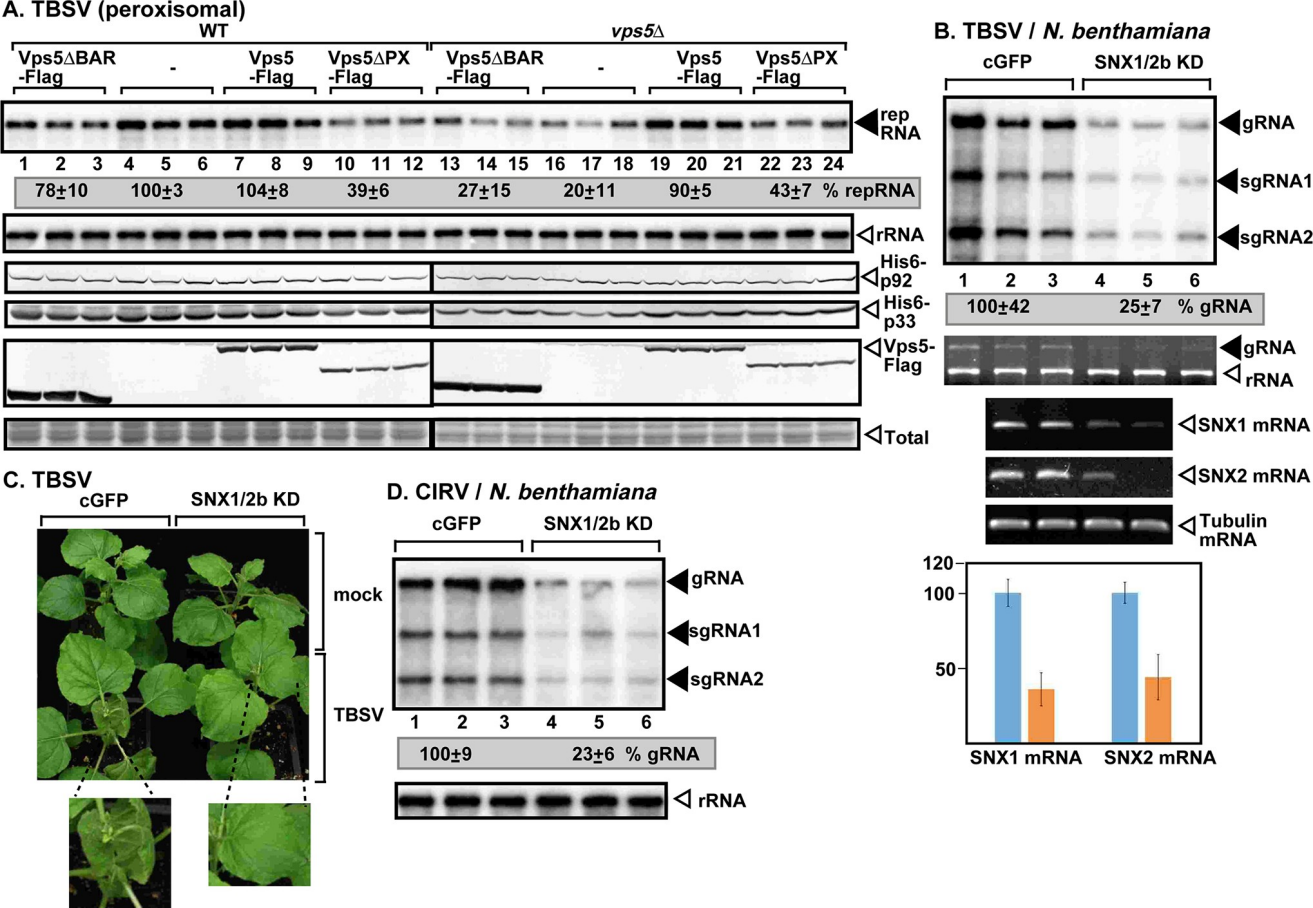
Pro-viral role of the endosomal SNX-BAR proteins in tombusvirus replication in yeast and plants. (A) Top image: Northern blot analysis shows decreased TBSV (+)repRNA accumulation in *vps5Δ* yeast strain. Vps5p and its deletion mutants were expressed from the constitutive *TEF1* promoter from a plasmid. The accumulation level of repRNA was normalized based on 18S rRNA levels (second panel). The accumulation of His_6_-p33, His_6_-p92 and Vps5-Flag is measured by western blotting and anti-His or anti-Flag antibodies. (B) VIGS-based knockdown (KD) of both Snx1 and Snx2b mRNA levels inhibits the accumulation of TBSV RNA in *N*. *benthamiana*. Top panel: The accumulation of TBSV gRNA and sgRNAs was measured using northern blot analysis of total RNA samples obtained from *N*. *benthamiana* leaves at 2 dpi. The upper, systemically-silenced leaves were inoculated with TBSV virions on the 12th day after VIGS. The control experiments included the TRV2-cGFP vector. Second panel: ethidium-bromide stained gels show ribosomal RNA level. Middle and bottom panels show the semi-quantitative RT-PCR and quantitative real-time PCR analyses of both Snx1 and Snx2b mRNA levels, whereas tubulin mRNA was used as a control in the VIGS plants. The Y-axis shows the relative levels of the Snx1 and Snx2b mRNAs with the control treatment (in blue) representing 100%. (C) Lack of phenotype in Snx1 and Snx2b knockdown *N*. *benthamiana*. The enlarged images show the delayed development of symptoms caused by TBSV infection in the silenced plants. The picture was taken 5 dpi. (D) VIGS-based knockdown of both Snx1 and Snx2b mRNA levels inhibits the accumulation of CIRV RNA in *N*. *benthamiana*. Samples for RNA extractions were taken 2.5 days post inoculation from the inoculated leaves. See further details in panel B. Each experiment was performed three times.

To test the putative role of the yeast PI(3)P-binding proteins in TBSV replication, we analyzed the accumulation of TBSV replicon (rep)RNA in yeast lacking one of fourteen known PI(3)P-effectors (S1 Table). We found that deletion of *VPS5* in haploid yeast showed the largest inhibitory effect on the accumulation of TBSV among the genes tested (S1 Table). The absence of Vps5p SNX-BAR protein, which is a PI(3)P-binding endosomal protein, resulted in ~5-fold inhibition of TBSV repRNA accumulation in *vps5Δ* yeast (Fig 1A, lanes 16–18 versus 4–6), confirming that Vps5p SNX-BAR is critical for TBSV replication in yeast. The closely-related CIRV, which, unlike the peroxisome-associated TBSV, replicates on the boundary membranes of mitochondria, was inhibited by ~6-fold in *vps5Δ* yeast (S1A Fig, lanes 16–18). Thus, Vps5p is required for tombusvirus replication in different subcellular environments.

Complementation through expression of the Flag-tagged Vps5p from a plasmid in *vps5Δ* yeast restored efficient replication of both TBSV and CIRV (Fig 1A, lanes 19–21 and S1A Fig). Over-expression of Vps5p in wt yeast did not increase TBSV or CIRV repRNA accumulation (Fig 1A, lanes 7–9 and S1A Fig). Expression of Vps5p lacking either the BAR domain or the PX domain could not complement the replication of the tombusvirus RNAs in *vps5Δ* yeast (Fig 1A, lanes 13–15 and 22–24, and S1A Fig), suggesting that tombusviruses need both domains of Vps5p for supporting viral replication. Since Vps5p forms either a homodimer or heterodimer with Vps17 [29], we tested TBSV replication in *vps17Δ* yeast, which supported ~3-fold less TBSV repRNA accumulation (S1B Fig, lanes 7–9). Thus, both Vps5p and Vps17p SNX-BAR proteins are important for tombusvirus replication in yeast.

To explore if tombusviruses depend on the Vps5p orthologs in plants, we knocked down both Snx1 and Snx2b levels via virus-induced gene-silencing (VIGS) in *Nicotiana benthamiana* plants. The sequence of Snx2a is very similar to Snx2b in *Arabidopsis* (S2, S3 and S4 Tables). Therefore, VIGS of Snx1/2b likely leads to silencing of both Snx2a (only incomplete sequence is available in *N*. *benthamiana*) and Snx2b in *N*. *benthamiana* plants. Knock-down of the SNX-BAR proteins led to ~4-fold reduced accumulation of TBSV genomic (g)RNA and CIRV gRNA, respectively (Fig 1B and 1D, lanes 4–6). Knocking down Snx1 and Snx2b levels ameliorated the necrotic symptoms caused by TBSV infection in *N*. *benthamiana* (Fig 1C). Similarly, protoplasts (separate cell wall-free plant cells) supported ~75% less replication of TBSV and CIRV gRNA when obtained from Snx1/Snx2b-silenced versus control *N*. *benthamiana* plants (S1C and S1D Fig). The replication of the more-distantly related turnip crinkle virus (TCV) was also reduced by ~3-fold in protoplasts obtained from Snx1/Snx2b-silenced versus control *N*. *benthamiana* plants (S1E Fig), suggesting that several plant viruses in the Tombusviridae family could take advantage of the SNX-BAR proteins during their replication. However, the pro-viral effects of the co-opted SNX-BAR proteins on tombusvirus replication seem to be specific, because deletion of *VPS5* in yeast did not have an adverse effect on the accumulation of the unrelated insect viruses, namely Nodamura virus (NoV) and Flock house virus (FHV) (S1G and S1H Fig).

In addition, we analyzed Snx1 and Snx2b mRNA levels in TBSV and CIRV-infected versus mock-treated *N*. *benthamiana* leaves. RT-PCR analysis showed the up-regulation of both Snx1 and Snx2b mRNA levels in tombusvirus-infected leaves (S1F Fig), suggesting that tombusvirus replication induces the expression of *SNX1* and *SNX2B* genes in plants.

### Tombusvirus replication proteins interact with and recruit the endosomal SNX-BAR proteins into VROs in yeast and plant cells

**Fig 2:**
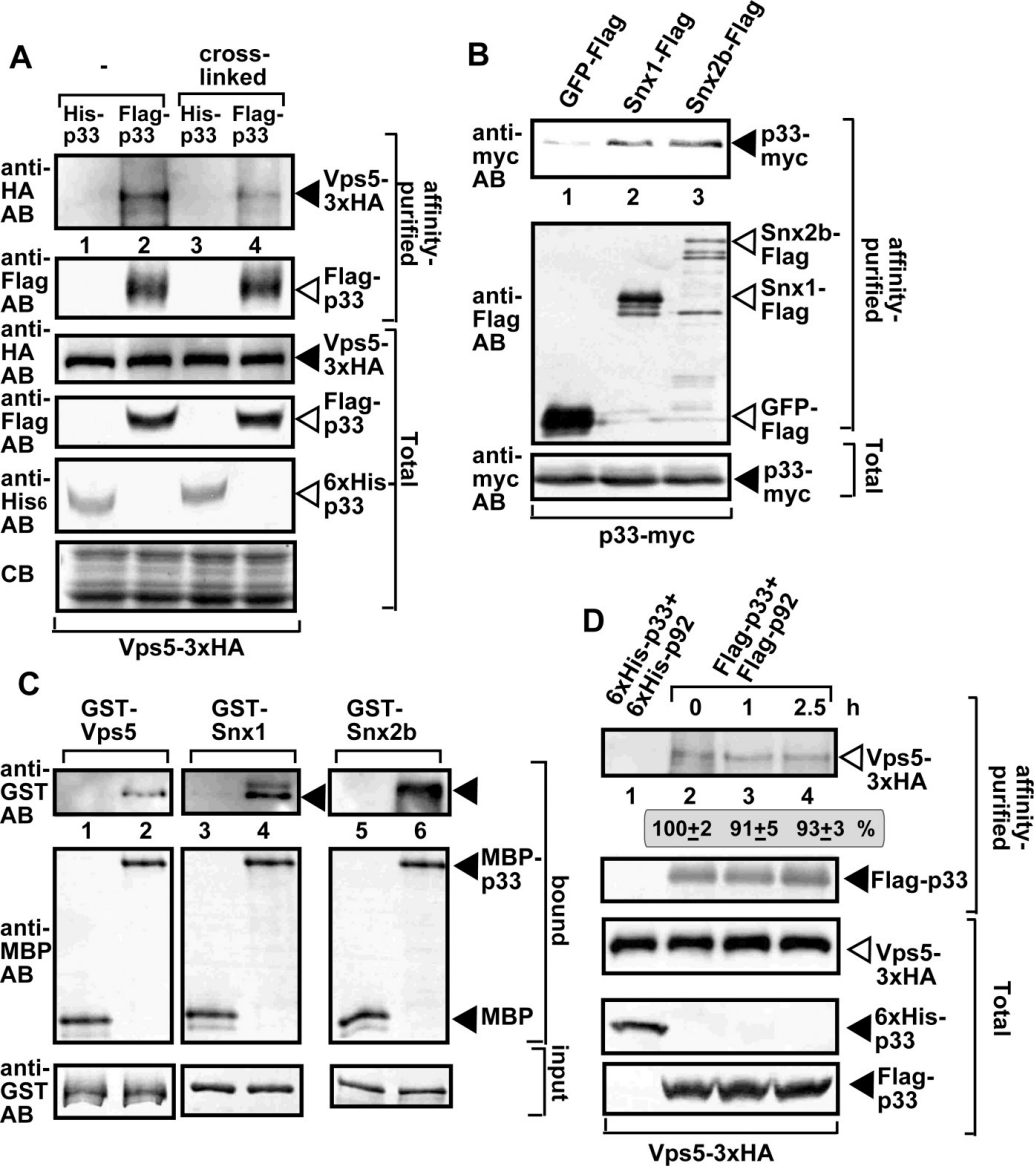
SNX-BAR proteins are present in the tombusviral replicase complex. (A) Co-purification of the yeast Vps5p with the TBSV VRCs. Top panel: Western blot analysis of co-purified 3xHA-tagged Vps5p (expressed from the native promoter in the original chromosomal location) with Flag-affinity purified p33 from membrane fraction of yeast. Vps5p was detected with anti-HA antibody. The negative control was His_6_-tagged p33 purified from yeast extracts using a Flag-affinity column (lanes 1 and 3). The yeast samples were either cross-linked with formaldehyde or not cross-linked. Second panel: Western blot of purified Flag-p33 detected with anti-Flag antibody. Bottom panels: Western blots of Vps5-3xHA, Flag-p33 and His_6_-p33 proteins in the total yeast extracts using anti-HA, anti-Flag and anti-His antibodies, respectively. (B) Co-purification of the myc-tagged p33 replication protein with the Flag-tagged Snx1 or Snx2b from detergent-solubilized membrane fraction of *N*. *benthamiana* cells. The proteins were co-expressed from plasmids based on agroinfiltration. The plants were also infected with TBSV. (C) A pull-down assay to test direct binding between the p33 replication protein and the shown SNX-BAR proteins expressed in *E*. *coli*. The MBP-tagged p33 was immobilized on beads, followed by addition of the affinity-purified GST-tagged SNX-BAR proteins. MBP was used as a negative control. Western blot analysis with the shown antibodies was used to detect the bound or input proteins. (D) Vps5p SNX-BAR is a permanent component of the TBSV replicase complex. Cycloheximide was used to block the formation of new VRCs in wt yeast via inhibition of cellular translation. Top panel: Western blot analysis shows the co-purified Vps5-3xHA with the viral replicase using Flag-based purification from the membrane fraction at the shown time points. Second panel: Western blot analysis of the purified Flag-p33 with anti-Flag antibody. Bottom panels: Western blot analysis of Vps5-3xHA, His_6_-p33 and Flag-p33 in the total yeast lysates with anti-HA, anti-His and anti-Flag antibodies, respectively. See further details in panel A. Each experiment was repeated three times.

To test if Vps5p interacts with the viral replication proteins, we performed co-purification experiments with Flag-tagged viral replication proteins from yeast membranes. The HA-tagged Vps5p was expressed in yeast from its native promoter and natural chromosomal location. Western blot analysis of the purified functional replicase complex identified the co-purified Vps5p (Fig 2A, lanes 2 and 4). Similarly, we were able to co-purify the myc-tagged TBSV p33 replication protein with either the Flag-tagged Snx1 or Snx2b from membrane fraction of plant cells (Fig 2B). Pull-down experiments with purified proteins from *E*. *coli* showed the direct binding of the p33 replication protein with the yeast Vps5p and the plant Snx1 and Snx2b in vitro (Fig 2C).

**Fig 3:**
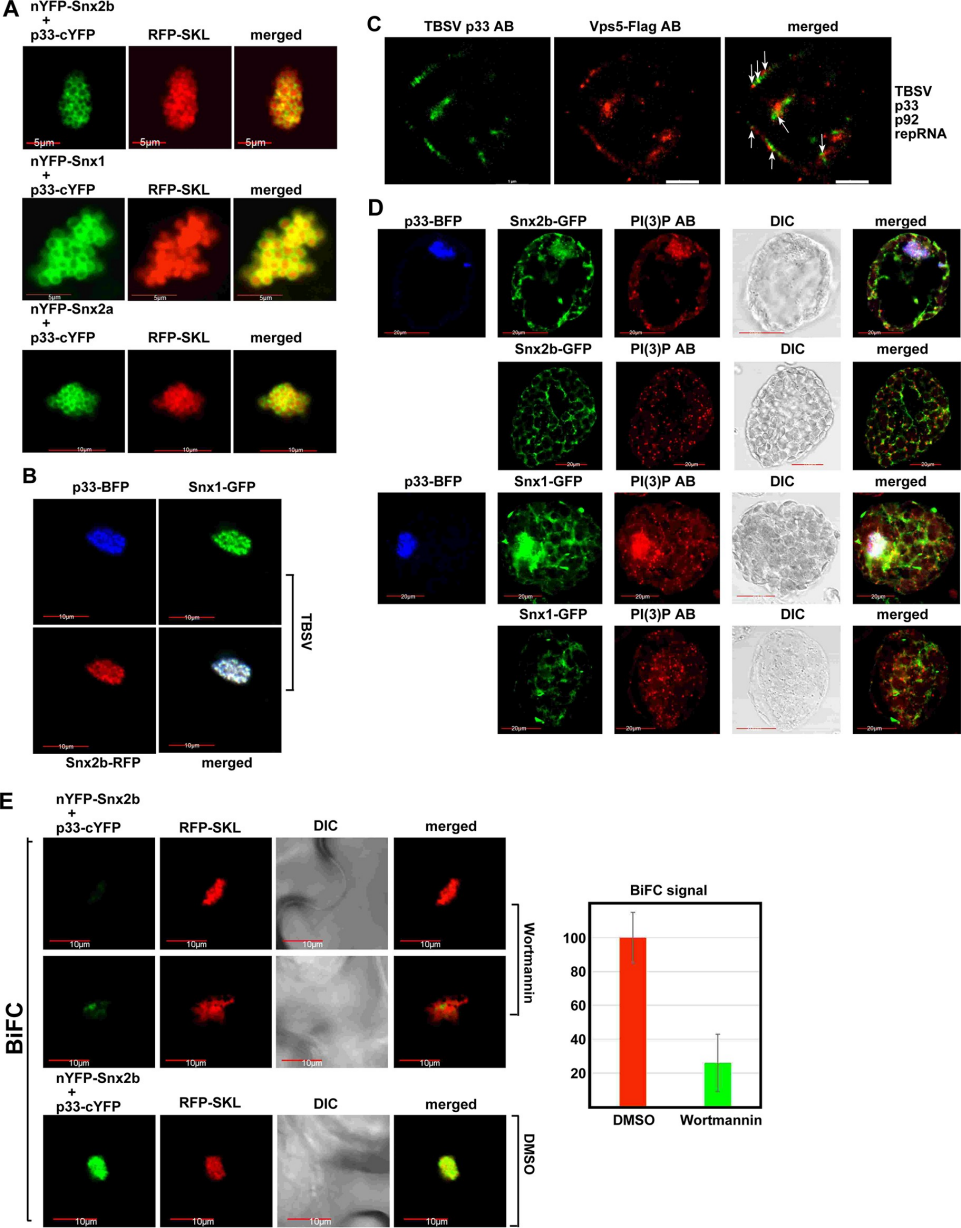
PI(3)P phosphoinositide is required for recruitment of the endosomal SNX-BAR proteins into the viral replicase. (A) BiFC assay to detect interaction between p33 replication protein and the SNX-BAR proteins *in planta*. TBSV p33-cYFP and nYFP-Snx2b, nYFP-Snx1 or nYFP-Snx2a proteins were co-expressed from the 35S promoter after co-agroinfiltration into *N*. *benthamiana* leaves. Note that the plants were infected with TBSV to induce VROs in cells. Co-localization of RFP-SKL (peroxisomal luminar marker) with the BiFC signal (see merged image) demonstrates that the interaction between p33 and SNX-BAR proteins occurs in VROs. Scale bars represent 5 μm (top two panels) and 10 μm (bottom panel). (B) Robust co-localization of TBSV p33-BFP with the GFP-tagged Snx1 and RFP-tagged Snx2b in *N*. *benthamiana* cells is detected by confocal laser microscopy. (C) Super-resolution laser microscopic images of yeast cells. The yeast cell replicating TBSV repRNA was imaged based on anti-TBSV p33 antibody and anti-Flag antibody for Vps5-Flag. The bars represent 1 μm. Arrows point at the co-localized Vps5p and p33 replication protein. The images were obtained by a Nikon N-STORM Super Resolution Microscope and image processing was performed using NIS-element software. Each experiment was repeated three times. (D) Co-localization of TBSV p33-BFP with the GFP-tagged Snx1 or GFP-tagged Snx2b and with PI(3)P in *N*. *benthamiana* protoplasts is detected by confocal microscopy. PI(3)P was detected with anti-PI(3)P antibody. Scale bars represent 20 μm. (E) BiFC assay shows the reduced level of interaction between p33 and the Snx2b protein *in N*. *benthamiana* treated with Vps34 PI3K inhibitor, Wortmannin or DMSO as a negative control. TBSV p33-cYFP and nYFP-Snx2b proteins were co-expressed from the 35S promoter after co-agroinfiltration into *N*. *benthamiana* leaves. RFP-SKL was expressed as a peroxisomal marker to identify VROs. Scale bars represent 10 μm. On the right, we show the quantitative evaluation of the BiFC signal with the Y-axis showing the relative BiFC signal levels with the control DMSO treatment (in red) representing 100%. The BiFC signals were quantified via Image J. Each experiment was repeated three times.

To gain insights into the subcellular site of interaction between Snx1, Snx2a and Snx2b with the replication proteins, we used bimolecular fluorescence complementation (BiFC) assay. This assay demonstrated that the p33 and p92^pol^ replication proteins interact with Snx1, Snx2a and Snx2b within the TBSV-induced aggregated peroxisomes, which represent the sites of TBSV replication (Figs 3A and S2A and S2B). The BiFC assay also showed that the CIRV p36 replication protein interacts with Snx1, Snx2a and Snx2b within the CIRV-induced aggregated mitochondria, which represent the sites of CIRV replication (S2C Fig). Confocal microscopy revealed that both Snx1 and Snx2b are recruited separately (S3A and S3B Fig versus S3C Fig), or simultaneously (Fig 3B) to the large TBSV VROs in plant cells and the yeast Vps5p in yeast cells (S3D and S3E Fig). Super-resolution microscopy showed the co-localization of Vps5 with the p33 and p92 replication proteins in yeast cells (Fig 3C). Based on these findings, we propose that TBSV co-opts the SNX-BAR proteins into the VROs through direct interaction with the viral replication proteins in yeast and plant cells.

Next, we tested if Vps5p is a permanent component of the viral replicase complex by purification of the viral replicase from yeast cells at various time points. New VRC formation was halted via blocking yeast translation through cycloheximide. Interestingly, the co-purified Vps5p level has not changed in comparison with Flag-p33 through 2.5 hours (Fig 2D). This finding indicates that Vps5p is a permanent resident in the membrane-bound viral replicase complex. Similar to Vps5p, we found that other co-opted cellular proteins, such as Hsp70s (Ssa1/2 in yeast), Vps4 ESCRT protein, and the DDX3-like Ded1 DEAD-box helicase, are also permanent residents in VRCs [21,38,39]. This is in contrast with other co-opted host factors, such as Vps34 PI3K, the glycolytic Pgk1, or Pex19, which are not present in the assembled VRCs [21,25,40].

### PI(3)P is required for the recruitment of the SNX-BAR proteins into VROs in yeast and plant cells

Confocal microscopy of plant protoplasts infected with TBSV revealed the efficient co-localization of PI(3)P (detected with anti-PI(3)P antibody or by using a RFP-2xFYVE biosensor, which specifically binds to PI(3)P in cells) [26,41], and Snx1 and Snx2b with the p33 replication protein at the replication sites (Figs 3D, S4A and S4B).

To test if PI(3)P affects the recruitment of SNX-BAR proteins into VROs, we performed BiFC assay with plant cells treated with Wortmannin, an inhibitor of Vps34 PI3K. The recruitment of both Snx1 (S5A–S5C Fig) and Snx2b (Figs 3E and S5C) into VROs was inhibited up to ~4-to-5 times by the reduced production of PI(3)P in plant cells treated with the PI3K inhibitor. Since PI(3)P is required for the endosomal localization of SNX-BAR proteins [42,43], and the TBSV p33 replication protein drives the recruitment of Rab5-positive endosomes into the TBSV VROs [24], the Wortmannin treatment likely inhibits the co-targeting of p33 and SNX-BAR proteins to the endosomes prior to final targeting to the VROs.

**Fig 4:**
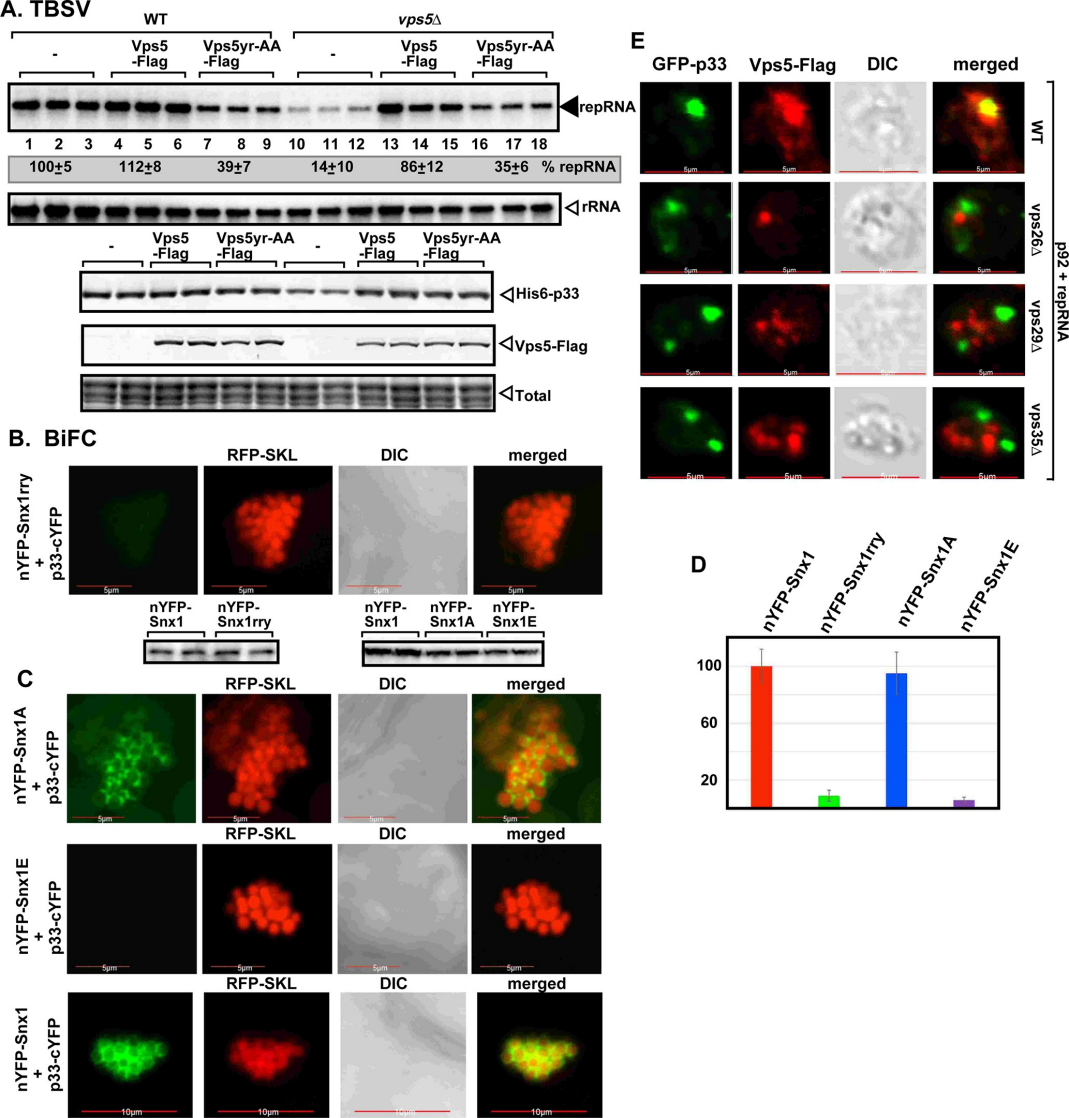
Mutations in the endosomal SNX-BAR proteins inhibit TBSV replication and their recruitment into VROs. (A) Vps5p mutant (Vps5^yr-AA^) deficient in PI(3)P-binding does not efficiently complement TBSV replication in vps5Δ yeast. Top panels: Northern blot analysis shows reduced TBSV (+)repRNA accumulation in wt yeast strain expressing Vps5^yr-AA^, and poor complementation of TBSV replication in vps5Δ yeast expressing Vps5^yr-AA^. Bottom images: Western blot analysis of the level of His_6_-p33 with anti-His antibody and Vps5-Flag with anti-Flag antibody, respectively. (B) Decreased interaction between TBSV p33-cYFP replication protein and the nYFP-Snx1^rry^ mutant protein, which is deficient in PI(3)P-binding, was detected by BiFC in *N*. *benthamiana* infected with TBSV. Scale bars represent 5 μm. Bottom image: The Western blots show the accumulation levels of the proteins used in these assays. (C-D) Interaction between TBSV p33-cYFP and the nYFP-Snx1 mutant proteins was detected by BiFC in *N*. *benthamiana* infcted with TBSV. Snx1^A^ is a non-phosphorylation mimicking mutant with high affinity for PI(3)P, whereas Snx1^E^ is a phosphorylation mimicking mutant with low affinity for PI(3)P. Scale bars represent 5, 5 and 10 μm. In panel D, we show the quantitative evaluation of the BiFC signals seen in panels B and C, with the Y-axis showing the relative BiFC signal levels with the wt Snx1 (in red) representing 100%. The BiFC signals were quantified via Image J. Each experiment was repeated three times. (E) Lack of recruitment of Vps5p into the p33-decorated VROs in yeast missing a retromer component. Vps5p was visualized with anti-Flag antibody. Scale bars represent 5 μm.

A mutagenesis approach was used to test if PI(3)P binding by SNX-BAR is important for TBSV replication. The conserved YR amino acids in the PI(3)P binding region (PX-domain) of the yeast Vps5p were mutated to AA to block PI(3)P binding [44]. Expression of Vps5^Y321A,R360A^ mutant in *vps5Δ* yeast poorly complemented TBSV replication (Fig 4A, lanes 16–18 versus 10–12 and S3D and S3E Fig), whereas Vps5^Y321A,R360A^ expression in WT yeast led to ~3-fold inhibition of TBSV replication (Fig 4A, lanes 7–9 versus 1–3), suggesting that Vps5^Y321A,R360A^ functions as a dominant negative mutant, likely as a component of a SNX-BAR dimer. Similar mutagenesis of the PI(3)P binding site in the plant Snx1 (Snx1^rry^) inhibited the recruitment into VROs by the TBSV p33 and the CIRV p36 replication proteins in a BiFC assay (Figs 4B and S5D). In addition, phosphorylation mimicking mutant of Snx1 [45], termed Snx1^E^, which has low binding affinity to PI(3)P, also interfered with the p33-driven recruitment of Snx1 into VROs, whereas nonphosphorylation mimicking mutant Snx1^A^, which has high binding affinity to PI(3)P, was recruited by TBSV similar to the case with wt Snx1 (Fig 4C and 4D). Pull-down experiments with purified recombinant proteins demonstrated that all the above Snx1 mutants still bound to p33 replication protein *in vitro* (S5E Fig). Therefore, the enrichment of PI(3)P is critical for TBSV to co-opt SNX-BAR proteins into VROs.

Since SNX-BAR proteins bind to PI(3)P and sense positive-membrane curvature, we wondered if it is possible to substitute the SNX-BAR proteins in the VRCs. We used the C-terminal domain of RavZ effector protein from *Legionella* as a fusion partner to p33 replication protein. RavZ^CT^ is known to bind to PI(3)P and senses positive-membrane curvature [46], similar to SNX-BAR proteins. Expression of RavZ^CT^-p33, however, strongly inhibited TBSV replication in yeast (S5F Fig). Deletion of the PI(3)P-binding module in RavZ^CT-P^-p33 resulted in fully functional replication protein (S5F and S5G Fig). These data suggest that the co-opted SNX-BAR protein—PI(3)P interaction is required for TBSV replication and this interaction cannot be replaced by p33—PI(3)P interaction in the form of RavZ^CT^-p33.

Next, we tested how Vps5p endosomal protein is delivered to the VROs in wt yeast. Vps5p functions together with the retromer complex, which consists of three conserved proteins, Vps26p, Vps29p and Vps35p. Vps5p and the retromer induce the formation of tubular transport carriers from the endosomes to recycle cargos to the Golgi and ER or to the plasma membrane [29,47]. An analysis of Vps5p distribution revealed that the p33-decorated VROs lacked Vps5p in vps35Δ, vps26Δ or vps29Δ yeasts in contrast with the wt yeast (Fig 4E). We also tested the recruitment of the Snx2b SNX-BAR protein, the plant homolog of the yeast Vps5, into VROs in *N*. *benthamiana* cells. We found co-localization of Snx2b and the TBSV p33 replication protein with Vps26, Vps29, and Vps35 retromer complex proteins in the large TBSV VROs (S6A Fig). Snx2b and the components of the retromer complex are also co-localized in the absence of TBSV infection in *N*. *benthamiana*, but they are not localized in large punctate structures (S6B Fig). Taken together, it seems that the retromer is involved in facilitating the p33-driven recruitment of the endosomal SNX-BAR membrane-shaping proteins into the tombusvirus VROs. The functional roles of the retromer in TBSV replication will be presented elsewhere.

### PI(3)P and the SNX-BAR proteins coordinate the protection of the viral RNAs from ribonucleases *in vitro*

Although previous works have shown that the ESCRT machinery is co-opted by TBSV to form spherules [21,22], the ESCRT factors might not be enough to render the spherules stable enough for an extended period to provide full protection for the dsRNA replication intermediate. Because local enrichment of PI(3)P results in positive membrane curvature and the SNX-BAR proteins sense, bind and reshape membranes into positive curvature (tube-like forms) [48], these host components are the best candidates to stabilize VRCs, including the neck structure of the tombusvirus VRCs (see model below). The narrow neck structure is proposed to serve as a gate of the VRCs, preventing ribonucleases to enter the VRCs, while allowing ribonucleotides entry and (+)RNA export out of VRCs during replication [4].

**Fig 5:**
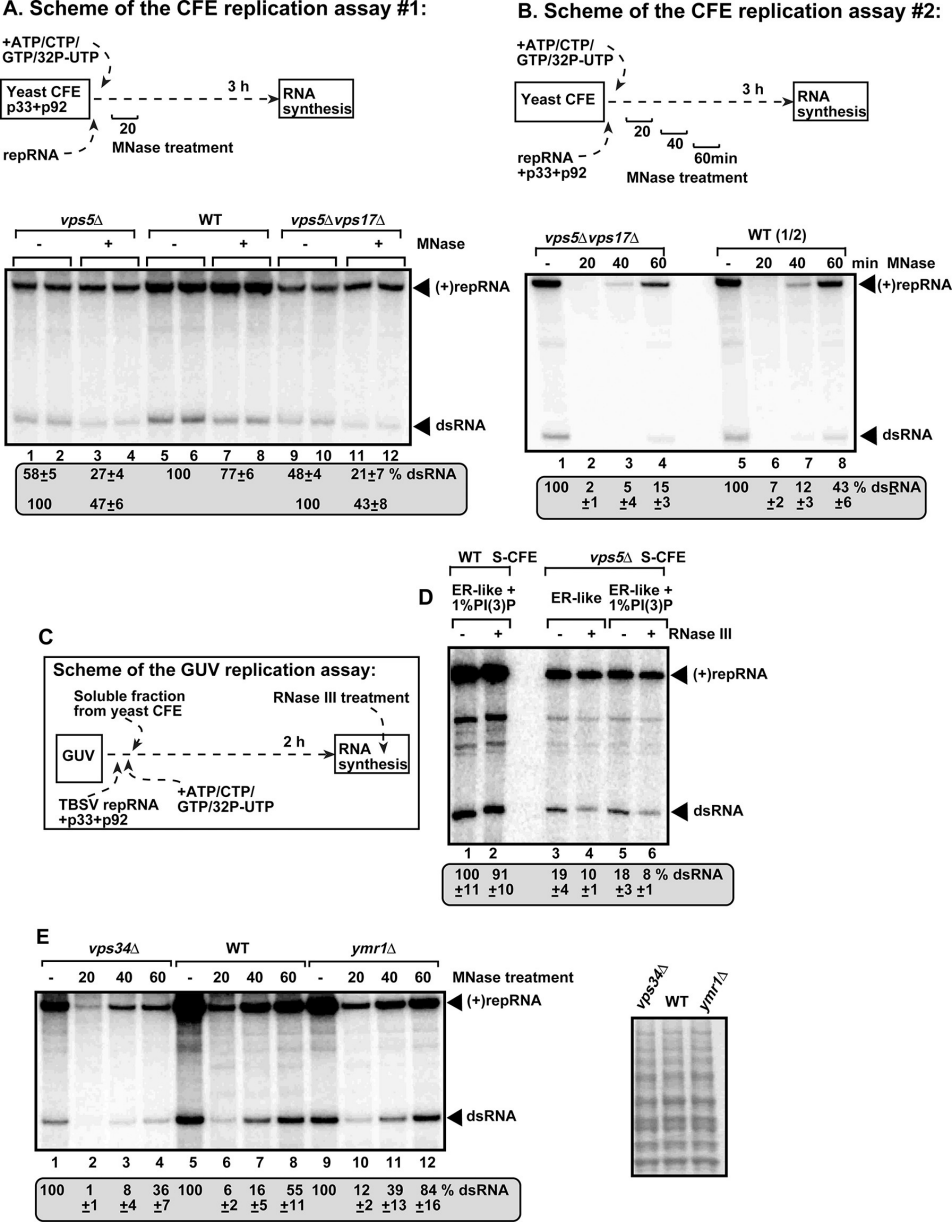
Vps5 SNX-BAR protein and PI(3)P are required for TBSV replication and for the protection of the viral dsRNA in replicase reconstitution assays. (A) Reduced dsRNA and (+)repRNA production and increased nuclease sensitivity by the tombusvirus replicase assembled *in vitro* in CFEs prepared from *vps5Δ* or *vps5Δvps17Δ* yeast strains. The yeast strains expressed the p33 and p92^pol^, allowing the pre-assembly of the replicase complex in yeast. (+)repRNA was used to program the CFEs, which were treated with MNase 20 min latter, followed by the inactivation of MNase with EGTA after 15 min. Non-denaturing PAGE analysis shows the ^32^P-labeled TBSV repRNA products, including the (+)repRNA progeny and the dsRNA replication intermediate, made by the reconstituted replicases. We also show the relative protection level of the dsRNA replication intermediate from the MNase treatment by adjusting the “no treatment” to 100% (see numbers in the bottom row). (B) *In vitro* reconstitution of the TBSV replicase using purified recombinant p33 and p92^pol^ and *in vitro* transcribed TBSV DI-72 (+)repRNA. The CFEs were prepared from the shown yeast strains. The MNase treatments, which lasted for 15 min, were done at three different time points as shown. Non-denaturing PAGE analysis was done as in panel A. Note that we loaded only half of the samples from the wt CFE-based assay. (C) The scheme of the novel GUV-based replicase reconstitution assay. (D) Both Vps5p SNX-BAR protein and PI(3)P are required to protect the TBSV dsRNA in a replicase reconstitution assay. GUVs were prepared with ER-like phospholipid composition (see also panel C). The soluble fraction of CFEs prepared from the shown yeast strains together with purified recombinant p33 and p92^pol^ and TBSV (+)repRNA were added to GUVs to reconstitute the replicase. The dsRNA-specific RNase III was added at the end of the reaction to destroy the unprotected TBSV dsRNA. Non-denaturing PAGE analysis was done as in panel A. (E) The Vps34 PI3K and Ymr1 PI(3)P phosphatase affect TBSV replication and the nuclease sensitivity of the viral dsRNA *in vitro*. The CFEs were prepared from wt, *vps34Δ* or *ymr1Δ* yeast strains to reconstitute the TBSV replicase as shown in panel B above. The MNase treatment and the RNA product analysis were as described in panel B. The panel on the right shows the comparable levels of yeast proteins in the obtained CFEs in an SDS-PAGE analysis.

To examine if the membranous VRCs in *vps5Δ* yeast indeed provide reduced protection to the viral RNAs against nucleases, we used several cell-free extract (CFE)-based replicase reconstitution assays, which can efficiently probe the exposure of the replicating viral RNAs to ribonucleases [49,50]. In assay #1, we expressed p33 and p92^pol^ replication proteins without the TBSV repRNA to allow the replicase pre-assembly step in *vps5Δ* yeast and in WT yeast as a control (Fig 5A) [50]. Then, we programmed the obtained CFEs with the TBSV (+)repRNA, followed by addition of the micrococcal nuclease (MNase) at the 20 min time point and inactivation of MNase 15 min latter. At the end of the assay, we measured the level of the TBSV dsRNA replication intermediate [produced by minus-strand synthesis on the (+)RNA template], which is always present in the VRCs [51]. The TBSV dsRNA was more sensitive to MNase when the CFEs were prepared from either *vps5Δ* or *vps5Δvps17Δ* yeast strains in comparison with the CFE from WT yeast (Fig 5A).

Additional assay to test the level of protection provided by the VRCs included *in vitro* replicase reconstitution with purified recombinant viral proteins and (+)repRNA transcripts as schematically shown in Fig 5B. This CFE-based assay supports a single full cycle of RNA replication, including both (-) and (+)RNA synthesis [49]. The MNase was added at different time points (as shown) for 15 min to destroy the unprotected viral RNAs, followed by MNase inactivation with EGTA and TBSV repRNA replication on the protected TBSV dsRNAs up to 3 hours (Fig 5B). The *in vitro* assembled VRC based on the CFE prepared from *vps5Δvps17Δ* yeast provided 3-times less protection at the 60 min time point in comparison with the CFE from WT yeast. In the third assay, we isolated the VRCs replicating TBSV repRNA from *vps5Δ* and WT yeasts, followed by *in vitro* replication assay in the presence or absence of MNase [52]. The activity of VRC preparation obtained from *vps5Δ* yeast was inhibited by the MNase by 2-fold more efficiently than the corresponding preparation from WT yeast (S7A and S7B Fig). Altogether, the results from three separate *in vitro* replication assays with VRCs lacking Vps5p showed that ribonucleases could access the TBSV dsRNA replication intermediate much more efficiently, likely due to incomplete or unstable VRCs. All these data support the model that the co-opted SNX-BAR protein—PI(3)P interaction helps stabilizing the VRCs and protection of the viral dsRNA during TBSV replication.

To obtain additional evidence for the protective role of Vps5p SNX-BAR protein in TBSV replication, we used a replicase reconstitution assay based on giant unilamellar vesicles (GUVs) [53]. The artificially made GUVs allow the selection of the particular lipid composition of membranes, whereas the soluble fraction of the CFE provides the host factors needed for TBSV replication (Fig 5C). Interestingly, in the absence of Vps5p, the reconstituted TBSV replicase could not efficiently protect the TBSV dsRNA replication intermediate from the dsRNA-specific RNase III (Fig 5D, lanes 4 and 6 versus 3 and 5). In contrast, GUVs in combination with the soluble fraction of the WT yeast CFE provided almost complete protection of TBSV dsRNA replication intermediate against the dsRNA-specific RNase III (Fig 5D, lanes 1–2). These experiments also illustrate the power of using GUV-based replicase reconstitution to study the effects of particular lipids in viral replication.

Similar *in vitro* replicase reconstitution using CFE preparations obtained from *vps34Δ* yeast, lacking PI3K, provided lesser protection of dsRNA against MNase than WT CFE did (Fig 5E). In contrast, *in vitro* replicase reconstitution using CFE preparations obtained from *ymr1Δ* yeast, lacking PI(3)P phosphatase, increased dsRNA protection against MNase in comparison with the WT CFE (Fig 5E). Moreover, WT CFE preparations programmed with purified recombinant viral proteins and (+)repRNA transcripts in the presence of purified recombinant Ymr1 PI(3)P phosphatase to reduce PI(3)P level in membranes by converting PI(3)P to PI [54] showed less protection of the dsRNA replication intermediate against MNase treatment (S7C and S7D Fig). Altogether, all these in vitro data obtained by probing the exposure of the viral dsRNA during viral replication strongly support the protective role of SNX-BAR protein and PI(3)P phosphoinositide to the TBSV dsRNA replication intermediate within VRCs.

### Co-opting the SNX-BAR proteins and enrichment of PI(3)P are needed to build RNAi-insensitive tombusvirus replication complexes

**Fig 6:**
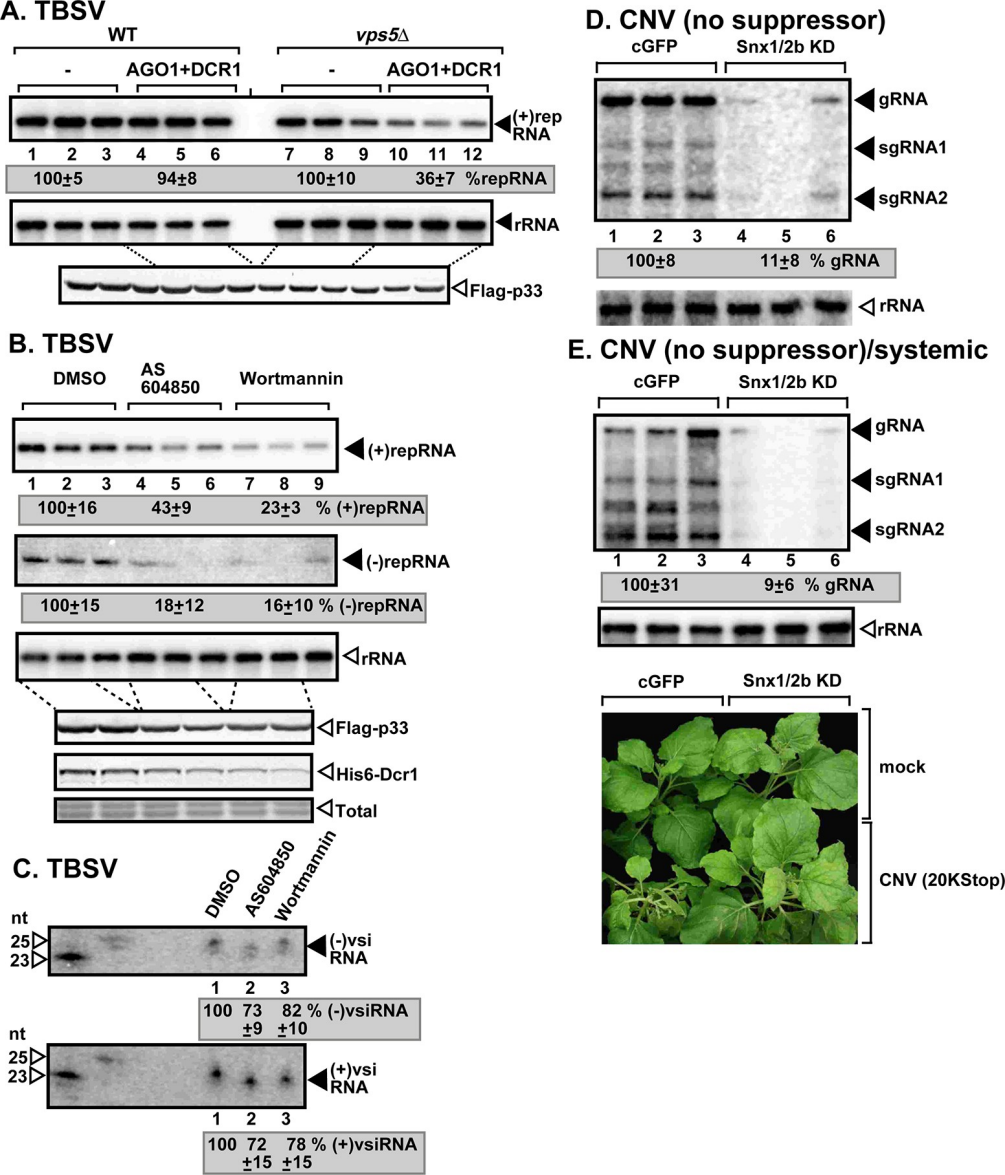
Depletion of SNX-BAR proteins or PI(3)P renders tombusvirus RNA sensitive to RNAi-based degradation in yeast and plants. (A) Co-expression of *S*. *castellii* Ago1 and Dcr1 proteins in *vps5Δ* yeast reduces TBSV repRNA accumulation to a larger extent than in wt yeast (BY4741). Replication of the TBSV repRNA was measured by northern blotting 16 h after initiation of TBSV replication. The accumulation level of repRNA was normalized based on the ribosomal (r)RNA. Each sample is obtained from different yeast colonies. Yeast strain not expressing RNAi components is chosen as 100%. Each experiment was repeated three times. (B) Inhibition of Vps34 PI3K with chemical inhibitors reduces TBSV (+)repRNA and (-)repRNA accumulation. Dcr1 was expressed in all yeast in these experiments. (C) Northern blot detection of vsiRNA(-) and vsiRNA(+) in the combined samples presented in panel B. The ^32^P-labeled TBSV DI-72 (+)RNA and (-)RNA, respectively, were used as probes. (D-E) VIGS-based knock-down of both Snx1 and Snx2b mRNA levels inhibits the accumulation of CNV^20Kstop^ RNA in *N*. *benthamiana*. The accumulation of CNV^20Kstop^ gRNA and sgRNAs was measured using northern blot analysis of total RNA samples obtained from *N*. *benthamiana* inoculated (3 dpi) (D) or systemically-infected (E) leaves (6 dpi). Note that CNV^20Kstop^ used for infection does not express p20 silencing suppressor protein in these samples, thus allowing the full effect of the RNAi response. See further details in Fig 1B.

To further test if Vps5p SNX-BAR protein is needed to protect the viral RNA in yeast, we have used the reconstituted RNAi machinery from *S*. *castellii* with the two-component *DCR1* and *AGO1* genes [55]. We measured TBSV accumulation when RNAi activity was induced [22] in WT as well as vps5Δ yeasts. Activation of RNAi in vps5Δ yeast led to ~3-fold less TBSV repRNA accumulation, suggesting poor protection of the TBSV RNA in comparison with vps5Δ yeast with suppressed RNAi machinery (Fig 6A, lanes 10–12 versus lanes 7–9). Induction of the RNAi machinery in WT yeast had a minor effect on TBSV accumulation (Fig 6A, lanes 4–6). Based on these data, we suggest that Vps5p SNX-BAR protein is involved in protecting the viral dsRNA replication intermediate within VRCs.

To test the role of PI(3)P phosphoinositide in the protection of the TBSV dsRNA replication intermediate, we measured vsiRNA abundance in yeast treated with Vps34p PI3K inhibitors in comparison with the DMSO-treated wt yeast expressing Dcr1 (Fig 6B). Interestingly, the abundance of vsiRNA(-) and vsiRNA(+), the products of Dcr1, did not change in yeast treated with PI3K inhibitors versus DMSO (Fig 6C lanes 2–3 versus 1). This observation is in contrast with the ~4-to-6-fold reduction of the target viral RNA level in yeasts treated with PI3K inhibitors versus DMSO (Fig 6B lanes 4–9 versus 1–3). Altogether, the data suggest that depletion of PI(3)P in yeast makes the TBSV VRCs highly accessible to Dcr1, resulting in efficient production of vsiRNAs.

To test if Snx1 and Snx2b proteins are needed to protect the viral RNA in plants, we knocked down Snx1 and Snx2b levels via VIGS and used CNV^20Kstop^ missing the p20 silencing suppressor to allow for efficient RNAi activity during infection. CNV^20Kstop^ accumulation was greatly suppressed in the inoculated leaves and CNV^20Kstop^ was close to undetectable in systemic leaves of *N*. *benthamiana* (Fig 6D and 6E). Knock down of the Snx1/Snx2b levels in *N*. *benthamiana* had a lesser inhibitory effect on other tombusviruses expressing the p19 suppressor of gene silencing (Fig 1B and 1D). Based on these data, we propose that tombusviruses require SNX-BAR proteins to protect their replicating RNAs against RNAi response in plants.

### The SNX-BAR proteins bind to the viral RNA in tombusvirus replication complexes

**Fig 7:**
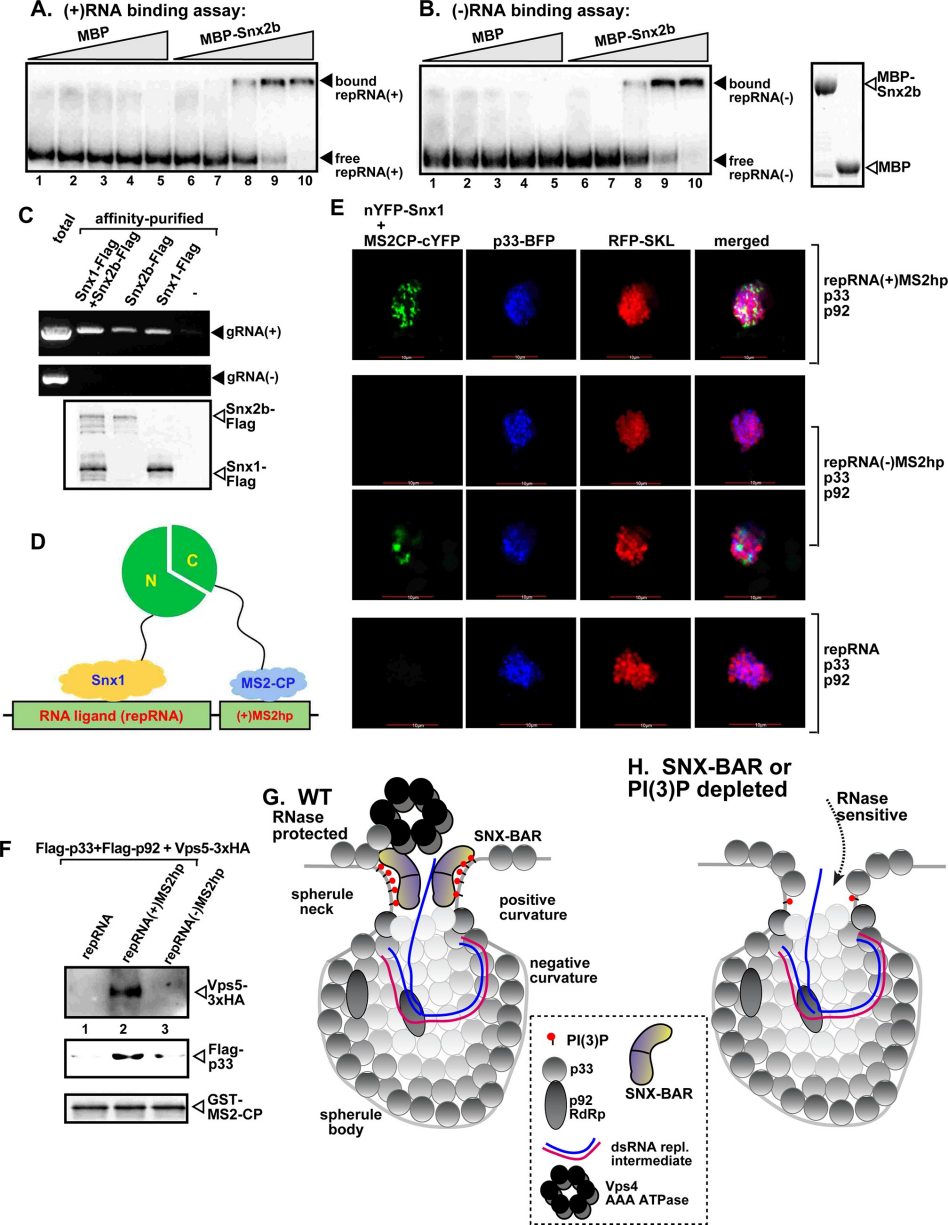
The co-opted SNX-BAR proteins bind to the TBSV (+)RNA. (A-B) *In vitro* RNA binding assay with purified Snx2b using ^32^P-labeled ssRNA templates. The assay contained the (-) or (+) DI-72 repRNA (~0.1 pmol) plus increasing amounts (1, 3, 6, 15, and 30 pMol) of purified recombinant MBP-tagged Snx2b or MBP as a negative control. RNA gel shift analysis was conducted on nondenaturing 5% PAGE that separated the free or Snx2b-bound ssRNAs. (C) Co-purification of the TBSV (+)gRNA with Snx1 or Snx2b from *N*. *benthamiana* replicating TBSV. The membrane fraction of plants expressing Flag-tagged Snx1 or Snx2b was solubilized with Triton-X100, followed by Flag-affinity purification. The agarose gel image shows the RT-PCR analysis of the co-purified (+)gRNA (top panel) or (-) gRNA (middle panel). Bottom panel: Western blot analysis of the Flag-affinity purified proteins from the samples shown above using anti-Flag antibody. (D) Scheme of the trimolecular fluorescence complementation (TriFC)-based Snx1-repRNA interaction. The repRNA [repRNA(+)MS2hp] carries the MS2 hairpin sequence that is specifically recognized by MS2-CP-cYFP. Binding of nYFP-Snx1 to the repRNA and simultaneous binding of MS2-CP-cYFP to the MS2-hairpins present in the repRNA is expected to generate TriFC signals as shown schematically. (E) TriFC analysis of Snx1 binding to the repRNA(+)MS2hp or repRNA(-)MS2hp (the MS2 hairpin folds on the minus-stranded repRNA). Bottom panel: repRNA lacking the MS2 hairpin sequence is used as a negative control. (F) Co-purification of Vps5p SNX-BAR protein with the help of repRNA(+)MS2hp serving as an RNA bridge. Purified recombinant GST-tagged MS2-CP was immobilized on the GST resin. Then, the detergent-solubilized yeast lysates containing Flag-p33, Flag-p92 and Vps5-3xHA and the shown repRNA derivatives were applied to the column. Please find the scheme for this assay in S7G Fig. Western blot analysis was used to document the captured Vps5-3xHA and Flag-p33, respectively. (G) A model showing the proposed roles of PI(3)P and the SNX-BAR proteins in stabilizing the neck structure of a spherule harboring the TBSV VRC. Enrichment of PI(3)P by the co-opted Vps34 PI3K in coordination with binding of the SNX-BAR dimer reshapes the membrane area into positive curvature. The body of the spherule is formed with the help of the co-opted ESCRT proteins. The viral (+)RNA is likely contacted by the SNX-BAR proteins during the exit of the viral RNA out of the spherule. (H) A model showing the increased ribonuclease sensitivity of the VRC if the neck structure is not stabilized with the contribution of PI(3)P and the SNX-BAR proteins.

We hypothesized that SNX-BAR proteins would likely contact the viral (+)RNA, while the RNA passes through the tight neck structure during exit from the spherule/VRC. Therefore, we tested if SNX-BAR proteins could bind to the viral RNA. In the first test, we used ^32^P-labeled (+)repRNA and (-)repRNA of TBSV and purified yeast Vps5p, Snx1 (S7E and S7F Fig) and Snx2b (Fig 7A and 7B) in a gel mobility shift assay. All three SNX-BAR proteins bound to the (+)RNA and (-)RNA *in vitro*, indicating that these host proteins are indeed RNA-binding proteins.

To demonstrate if similar interaction could occur in plant cells, we cross-linked plant samples replicating the TBSV gRNA, followed by purification of Flag-tagged Snx1 and/or Snx2b. RT-PCR analysis of the RNAs isolated from purified Snx1, Snx2b and Snx1/Snx2b preparations with strand-specific primers showed the presence of the co-purified TBSV (+)gRNA, but not the (-)RNA (Fig 7C). In the second test, we used a three-component TriFC-based assay (Fig 7D). The RNA component was a modified repRNA carrying an ssRNA sensor consisting of six repeats of a hairpin RNA (MS2hp) from MS2 bacteriophage, which is specifically recognized by the MS2 coat protein (MS2-CP) (Fig 7D) [56]. Co-expression of the repRNA(+)MS2hp and MS2-CP-cYFP and nYFP-Snx1 with TBSV p33 and p92^pol^ replication proteins leads to replication of the repRNA. If nYFP-Snx1 could bind to the repRNA, whereas MS2-CP-cYFP could bind to the MS2-hairpins present in the repRNA, then we expect TriFC signal as shown schematically in Fig 7D. Interestingly, we found many plant cells (93%) with the TriFC signals, where p33-BFP marked the sites of TBSV replication (Fig 7E, top panel). In contrast, it was difficult to find TriFC signals when the MS2 hairpins formed on the (-)repRNA, although a few cells (only 9%) had weak TriFC signals as shown in Fig 7E. In the control experiments, the TBSV repRNA replaced repRNA(+)MS2hp and there was no TriFC signal within the plant cell compartments marked by p33-BFP (Fig 7E, bottom panel). Therefore, we conclude that repRNA(+)MS2hp was required as an “RNA bridge” to allow the proximal positioning of MS2-CP-cYFP and nYFP-Snx1 within the membranous TBSV VROs marked by p33-BFP. Therefore, we suggest that either Snx1 is located in the proximity of the TBSV (+)repRNA or Snx1 binds to the viral (+)RNA during viral replication.

To confirm that the yeast Vps5p also interacts with the viral RNA *in vivo*, we co-expressed repRNA(+)MS2hp with p33 and p92^pol^ replication proteins (to support replication) and HA-tagged Vps5p in yeast. This was followed by capturing repRNA(+)MS2hp from yeast with the GST-tagged MS2-CP (S7G Fig). Interestingly, we detected both Vps5p and the p33 replication protein in the eluate from the GST resin (Fig 7F, lane 2). When we expressed repRNA(-)MS2hp under the above conditions, then the eluate from the GST resin with the bound GST-MS2-CP contained only p33, albeit to a lesser amount than that obtained with repRNA(+)MS2hp (Fig 7F lanes 3 versus 2). In the control experiments, expression of the replication proteins with the TBSV repRNA, lacking the (+)MS2hp sequence, did not result in the co-purification of Vps5p and the p33 replication protein with the GST-MS2-CP (Fig 7F, lane 1). Based on these results, we suggest that Vps5p binds to the viral (+)RNA within the VRCs, either directly or in complex with the p33 replication protein, during viral replication in yeast.

## Discussion

Many (+)RNA viruses, including TBSV, induce the formation of large VROs harboring numerous membranous spherules, small vesicle-like structures during their replication [6–8]. These spherules contain the VRCs, which produce vast amounts of progeny viruses in a short time period in infected cells [2,4,8,10]. The emerging theme is that the formation of viral spherules is a complex process that requires viral replication factors, co-opted host factors, and altered lipid composition of the targeted membranes. However, the actual host proteins and lipids within individual VRCs/spherules are not completely known.

In this paper, we have identified the critical roles of endosomal SNX-BAR proteins and PI(3)P phosphoinositide in the formation and function of TBSV VRCs. The yeast Vps5p and the plant Snx1 and Snx2b SNX-BAR proteins are efficiently co-opted and retargeted into the large TBSV-induced VROs consisting of aggregated peroxisomes or the CIRV-induced aggregated mitochondria. The recruitment of SNX-BAR proteins to the sites of viral replication required PI(3)P, which is produced by the co-opted Vps34p PI3K in yeast and plants, as we have shown previously [25]. Vps5p SNX-BAR protein is a permanent component of the TBSV VRCs, suggesting that SNX-BAR proteins might have structural functions within the VRCs. This is also supported by the observations that functionally deficient mutants of SNX-BAR protein could act as dominant negative during TBSV replication. What could be the structural roles of SNX-BAR proteins and PI(3)P in the TBSV VRCs?

The spherules consist of two different membranous subdomains: the body, which harbors the VRC and shows negative curvature, and likely enriched with PE (a negative membrane curvature-inducing lipid) and sterols (Fig 7G). The second subdomain is the tiny “neck” structure, which is proposed to operate as a restrictive gate, allowing the entry of ribonucleotides and the exit of the newly made (+)RNA progeny. However, it keeps the VRC and the dsRNA replication intermediate inside the spherule body structure. This neck structure is assumed to provide protection against the host antiviral surveillance apparatus and prevent destruction of the viral dsRNA by host ribonucleases. TBSV-induced spherule formation depends on the co-opted ESCRT machinery, which bends the target membrane-domain inward towards the lumen of the peroxisomes in case of TBSV and CNV, whereas the ESCRT machinery remodel the outer membrane-domain of mitochondria toward the matrix in case of CIRV [21,22,57]. In the absence of the ESCRT proteins, tombusviruses could only induce the formation of open, “deep dish-like” structures. Because the neck structure consists of a membrane subdomain with high positive curvature, based on our data, we propose that the neck subdomain is highly enriched with PI(3)P phosphoinositide, which is known to induce positive curvature in membranes [58]. The presence of PI(3)P would allow the binding of the SNX-BAR proteins via their PX domain. Also, the BAR domain in the SNX-BAR proteins could sense, induce and/or stabilize the positive curvature within the neck structure (Fig 7G and 7H). Accordingly, both domains of the Vps5p SNX-BAR protein were required to for efficient TBSV replication (Fig 1A). The critical nature of the co-opted SNX-BAR—PI(3)P interaction is supported by the strong inhibitory effect of the expression of RavZ^CT^-p33, but not RavZ^CT-P^-p33 lacking the PI(3)P-binding domain. The dominant inhibitory effect of RavZ^CT^-p33 is likely due to competition with Vps5p SNX-BAR to bind to PI(3)P within VRCs.

Importantly, SNX-BAR—PI(3)P interaction might stabilize VRCs, possibly the neck structure, which could be important to maintain continuous viral (+)RNA synthesis for several hours. This model is supported by the *in vitro* and *in vivo* data based on probing the extent of viral RNA protection by VRCs. For example, *in vitro* reconstitution of the TBSV replicase led to protective structure for the viral dsRNA against ribonucleases if wt yeast CFE was used. The reconstituted TBSV replicase was less protective to the dsRNA (i) in the absence of Vps5p SNX-BAR protein; (ii) when the amount of PI(3)P in the CFE was reduced by the addition of Ymr1 PI(3)P phosphatase; (iii) or in the absence of Vps34p PI3K. In contrast, the reconstituted TBSV replicase was more protective to the dsRNA in the absence of Ymr1 PI(3)P phosphatase. Moreover, yeast lacking Vps5p SNX-BAR protein provided lesser protection to the TBSV repRNA than wt yeast when a reconstituted RNAi pathway was activated. In comparison with the repRNA, the relative amount of vsiRNAs, produced by Dcr1 on dsRNA template, was increased when Vps34p PI3K was inhibited via chemical inhibitors. Moreover, knock down of the Snx1/Snx2b levels in *N*. *benthamiana* had a more dramatic inhibitory effect on a tombusvirus lacking the suppressor of gene silencing (Fig 6D and 6E) than on other tombusviruses expressing the p19 suppressor of gene silencing (Fig 1B–1D). All these data suggest that the SNX-BAR proteins in connection with PI(3)P are co-opted by tombusviruses, which then lead to efficient protection of the viral RNAs against cellular nucleases during viral replication. Altogether, we propose that the SNX-BAR proteins and PI(3)P could be hijacked by tombusviruses to stabilize VRCs, and possibly the neck structure within the TBSV-induced spherules. In the absence of either the SNX-BAR proteins or PI(3)P, the VRC and its neck structure might become more dynamic and possibly less stable and provide lesser protection of the viral dsRNAs against cellular nucleases during TBSV replication (Fig 7H).

The neck structure within the TBSV-induced spherules is rather narrow, ~10 nm in diameter [6,21]. The association of SNX-BAR proteins with the neck structure would make the actual opening even narrower, thus, placing the SNX-BAR proteins in the proximity of the exiting (+)RNA from the spherule (Fig 7G). Indeed, we were able to co-purify the viral (+)RNA, but not the (-)RNA, with Snx1 and Snx2b SNX-BAR proteins from plants. Moreover, capturing the viral (+)RNA also led the co-purification of both Vps5p and the p33 replication protein from yeast (Fig 7). Also, a novel TriFC-based method detected the association of the plant Snx1 with the viral RNA in plant cells (Fig 7). Direct binding of the plant Snx1/2b to the viral RNA was also observed *in vitro*. All these observations support the model that the co-opted SNX-BAR proteins are in close association with the viral (+)RNA, possibly during the temporal passing of the viral (+)RNA through the tight neck structure in VRCs. Future, ultrahigh-resolution structural studies will be needed to confirm this model.

The advantage to co-opt the endosomal SNX-BAR proteins to the replicase complex is that TBSV co-opts the endosomal Rab5-containing vesicles, which are PE-rich, and the endosomal Vps34 PI3K into VROs [24,25,28]. These early endosomal components contribute to the biogenesis of the large VROs in yeast and plant cells. Therefore, the endosomal SNX-BAR proteins should be readily accessible for subversion by the TBSV p33 replication protein.

The use of membrane curvature-sensing proteins during viral infections might be common among (+)RNA viruses. Accordingly, alphaviruses, semliki forest virus and Sindbis virus, co-opt the cellular amphiphysins for replication [59]. Brome mosaic virus and several flaviviruses hijack the ER-resident membrane curvature-sensing reticulons into VRCs [60,61]. Hepatitis C virus usurps the cellular PSTPIP2 membrane-deforming protein for VRO formation [62], whereas, HIV subverts PACSIN2 BAR-domain protein for cell-to-cell spread [63].

In conclusion, we have determined the critical roles of endosomal SNX-BAR proteins and PI(3)P phosphoinositide in the formation and function of TBSV VRCs. By co-opting the SNX-BAR proteins and enrichment of PI(3)P in VRCs, tombusviruses create a nuclease- and RNAi-protective microenvironment for viral replication.

## Materials and methods

Some of the Materials and Methods are presented in the S1 Text.

### Yeast stains

Parental yeast strain BY4741 (MATa his3Δ1 leu2Δ0 met15Δ0 ura3Δ0), and single knock out strains vps5Δ, vps17Δ, vps34Δ, vps26Δ, vps29Δ, vps35Δ and ymr1Δ were purchased from Open Biosystems. SC1 (MATa his3Δ1 leu2Δ trp1Δ289 uraΔ52) yeast strain was purchased from Invitrogen. vps5Δvps17Δ double deletion strain and yeast strain (BY4741: Vps5-3xHA) with chromosomal HA tagging of Vps5 was created by using yeast toolbox plasmids [64].

### Plant and yeast expression plasmids

Plasmids and primers used in this study are described in S5 Table.

### Analysis of viral replication in yeast and plant

To identify the function of yeast Vps5 and Vps17 SNX-BAR proteins in the replication of TBSV and CIRV, yeast strains BY4741, vps5Δ and vps17Δ were transformed with plasmids pESC-His-p33/DI72, pYES-His-p92 and pRS315-cFlag for TBSV replication, pESC-Strep-p36/DI72, pYES-Strep-p95 and pRS315-cFlag for CIRV replication. The transformed yeast cells were pre-grown in synthetic complete medium lacking uracil, leucine and histidine (ULH^-^) supplemented with 2% glucose at 29°C for overnight, then tombusviral repRNA replication was induced by transferring the yeast to synthetic complete medium (ULH^-^) supplemented with 2% galactose at 23°C for 24 h for TBSV or 30 h for CIRV. Yeast total RNA and total protein were isolated and analyzed by Northern blotting and Western blotting, respectively [14,25]. The functions of yeast Vps5 mutants in tombusviral replication were tested by using the same methods.

To measure the effect of plant SNX-BAR proteins on virus replication, NbSnx1 and NbSnx2b gene expression were silenced using tobacco rattle virus (TRV)-mediated virus-induced gene silencing (VIGS) method in *N*. *benthamiana* [65]. The NbSnx1 and NbSnx2b fragments were ligated together into the pTRV2 vector. The upper NbSnx1/2b-silenced leaves were inoculated with TBSV, CIRV or CNV^20KStop^ sap 12 day post agroinfiltration. The control plants were treated the same way, except using TRV-cGFP (The C terminus of GFP ORF was inserted into pTRV2 vector to prepare this control plasmid). Plant leaf discs from inoculated leaves and systemic leaves were collected for total RNA extraction and viral RNA detection by Northern blotting [66].

Plant protoplasts were isolated from NbSnx1/2b-silenced or cGFP-control *N*. *benthamiana* leaves [67]. About 5 x 10^5^ protoplasts were transformed with *in vitro* transcribed full-length TBSV, CIRV and TCV genomic RNAs, incubated in 35 x 10 mm petri dishes in dark at room temperature for 24 h. Total RNAs were extracted from these protoplasts, viral RNA accumulation was detected by Northern blotting [68].

### Confocal microscopy analysis of plant and yeast cells

To examine the subcellular localization of plant SNX-BAR proteins upon virus infection, *N*. *benthamiana* leaves were co-infiltrated with agrobacterium carrying plasmids pGD-AtSnx1-GFP (0.3 OD_600_) or pGD-AtSnx2b-GFP (0.3 OD_600_), pGD-RFP-SKL (0.2 OD_600_) together with pGD-p33-BFP (0.3 OD_600_) and p19 (0.1 OD_600_). Alternatively, pGD-p33-BFP, pGD-AtSnx1-GFP and pGD-AtSnx2b-RFP were co-agroinfiltrated into *N*. *benthamiana*. The agroinfiltrated leaves were inoculated with TBSV sap at 14 h post agroinfiltration. After 2 dpi, the agroinfiltrated leaves were subjected to confocal microscopy (FV1200 confocal laser scanning microscope, Olympus) using 405 nm laser for BFP, 488 nm laser for GFP and 559 nm for RFP. Images were captured successively and merged using the FLUOVIEW software [25].

To examine the co-localization of plant sorting nexins with the retromer complex upon virus infection, *N*. *benthamiana* leaves were co-infiltrated with agrobacterium carrying plasmids pGD-AtSnx2b-RFP (0.3 OD_600_), pGD-p33-BFP (0.3 OD_600_) and p19 (0.1 OD_600_) together with pGD-AtVps26-GFP(0.3 OD_600_) or pGD-AtVps29-GFP(0.3 OD_600_) or pGD-AtVps35-GFP(0.3 OD_600_). The agroinfiltrated leaves were subjected to confocal microscopy as above.

To analyze the subcellular localization of Vps5 in yeast cells upon tombusviral replication, pYes-His-p92, pEsc-GFP-p33/DI72 and pRS315-Vps5-Flag plasmids were transformed into BY4741 yeast. The transformed yeast cells were pre-grown in synthetic complete medium (ULH^-^) supplemented with 2% glucose at 29°C overnight. Tombusviral repRNA replication was induced by changing the yeasts to synthetic complete medium (ULH^-^) supplemented with 2% galactose for 21 h at 23°C. Yeast cells were collected to isolate spheroplasts for immunofluorescence [25]. Vps5-Flag was visualized with anti-Flag mouse antibody (Sigma-Aldrich, Cat#F1804), and Alexa 568 secondary antibody (Thermo Fisher Scientific, Cat#A11031). Confocal microscopy analysis using 488 nm laser for GFP and 559 nm for Alexa 568 fluorescent dye in an Olympus FV1200 confocal laser scanning microscope.

To analyze the subcellular localization of Vps5p in yeast retromer deletion stains upon tombusviral replication, pYes-His-p92, pEsc-GFP-p33/DI72 and pRS315-Vps5-Flag were transformed into vps26Δ, vps29Δ and vps35Δ yeast. Immunofluorescence analysis was conducted as above.

Yeast strain BY4741 was transformed with pESC-His-p33/DI72, pYES-His-p92 and pRS315-Vps5-Flag plasmids. Co-localization between p33 and Vps5 was analyzed by super-resolution microscopy (N-STORM Super Resolution Microscopy, Nikon). Anti-p33 monoclonal mouse antibody and anti-Flag rabbit antibody (Sigma-Aldrich, Cat#F7435) were used to visualize p33 and Vps5, respectively [69].

To identify the interaction between SNX-BAR proteins and tombusviral replication proteins *in vivo*, bimolecular fluorescence complementation (BiFC) assay was performed in *N*. *benthamiana* [24]. The plasmids pGD-p33-cYFP or pGD-p36-cYFP or pGD-p92-cYFP, pGD-nYFP-GST, pGD-RFP-SKL and pGD-CoxIV-RFP were transformed to *Agrobacterium* strain C58C1, as well as pGD-nYFP-AtSnx1, pGD-nYFP-AtSnx2b and SNX-BAR protein mutants. The obtained *Agrobacterium* transformants were co-infiltrated (0.3 OD_600_, each) into the leaves of four weeks-old *N*. *benthamiana* plants. Agroinfiltrated leaves were inoculated with TBSV or CIRV 14 h after agroinfiltration. Plant samples were subjected to confocal laser microscopy at 48 h post virus inoculation.

To observe the distribution and association between cellular PI(3)P and SNX-BAR proteins upon tombusvirus replication in plant cells, pGD-AtSnx1-GFP (0.3 OD_600_) or pGD-AtSnx2b-GFP (0.3 OD_600_), together with pGD-p33-BFP (0.3 OD_600_) were agroinfiltrated into *N*. *benthamiana* leaves. The agroinfiltrated leaves were inoculated with TBSV sap at 12 h post agroinfiltration. Protoplasts were isolated from the infiltrated leaves with enzyme solution containing 1.5% (wt/vol) Cellulase R10 (Yakult Pharmaceutical Ind. Co., Ltd., Japan) and 0.4% (wt/vol) Macerozyme R10 (Yakult Pharmaceutical Ind. Co., Ltd., Japan) 48 h post virus inoculation for immunofluorescence detection of PI(3)P [25]. The permeabilized cells were incubated with purified anti-PI(3)P mouse antibody (Echelon Biosciences Inc. Cat#Z-P003), and after washing steps, incubated with anti-mouse secondary antibody conjugated with Alexa Fluor 568 (Thermo Fisher Scientific, Cat#A11031). The images were captured with Olympus FV1200 confocal laser scanning microscope. RFP-2xFYVE was used as a PI(3)P biosensor to visualize PI(3)P distribution upon virus replication in plant protoplast system [25].

To investigate the role of PI(3)P in the recruitment of plant SNX-BAR proteins into viral replication compartments, the plasmids pGD-nYFP-AtSnx1, pGD-nYFP-AtSnx2b, pGD-p33-cYFP and pGD-RFP-SKL were separately transformed into *Agrobacterium* strain C58C1. The obtained *Agrobacterium* transformants were co-infiltrated (OD_600_ 0.3 for each) into the leaves of four week-old *N*. *benthamiana* plants. Agroinfiltrated leaves were treated with 66 μM Wortmannin (an Vps34 PI3K inhibitor) 15 h after agroinfiltration. Plant samples were subjected to confocal laser microscopy at 31 h post inhibitor treatment.

### Protein-protein interaction between SNX-BAR proteins and viral replication protein p33 by co-purification and pull down assays

To test the interaction between p33 and SNX-BAR proteins in plant, TBSV p33-myc was co-expressed with AtSnx1-Flag or AtSnx2b-Flag or GFP-Flag in plant by agroinfiltration (0.3 OD_600_, each), followed by TBSV sap inoculation 12 h post agroinfiltration. Membrane fraction was isolated from the leaves 2 dpi and solubilized with 1% Triton X-100 followed by purification of AtSnx1-Flag or AtSnx2b-Flag with anti-Flag M2 agarose (Sigma-Aldrich, Cat#A2220). The co-purified p33-myc protein was detected by anti-myc antibody [25].

To dissect whether Vps5 SNX-BAR protein is a permanent component of the tombusvirus replicase complex, pEsc-URA-Vps5-3xHA was transformed into yeasts with pGAD-Cup1-Flag-p92 and pGBK-Cup1-Flag-p33-Gal1-DI72. pGAD-Cup1-His-p92 and pGBK-Cup1-His-p33-Gal1-DI72 were transformed into yeast as the negative control. The transformed yeast cells were pre-grown in synthetic complete medium lacking uracil, leucine and histidine (ULH^-^) supplemented with 2% glucose and 100 μM BCS at 29°C overnight, followed by transferring the yeasts to synthetic complete medium (ULH^-^) supplemented with 2% galactose and 100 μM BCS at 29°C for 24 h. Tombusviral repRNA replication was induced by changing the media to synthetic complete medium (ULH^-^) supplemented with 2% galactose and 50 μM CuSO_4_ for 3 h at 23°C, 100 μg/ml cycloheximide was added to the culture to inhibit protein translation [21]. Yeast samples were collected at 0 h, 1 h and 2.5 h after addition of cycloheximide. Cross-linking of yeast cells was conducted by suspending yeast pellets with 1x PBS buffer containing 1% formaldehyde and incubation for 1 h on ice. Then, glycine (to 0.1 M) was added to quench the extra formaldehyde and the yeasts were washed and collected by centrifugation. Yeast cells were broken with glass beads, membrane fraction was solubilized with 1% Triton X-100 followed by purification of Flag-p33 with anti-Flag M2 agarose (Sigma-Aldrich, Cat#A2220). The co-purified protein was detected with anti-HA antibody.

We also performed the co-purification assay using yeast expressing Vps5-3xHA from its natural promoter and the original chromosomal location in wt yeast. Yeast expressing Vps5-3xHA was transformed with pGAD-Cup1-Flag-p92 and pGBK-Cup1-Flag-p33-Gal1-DI72. pGAD-Cup1-His-p92 and pGBK-Cup1-His-p33-Gal1-DI72 were transformed into yeast as the negative control. The procedure of yeast culture and purification was the same as above.

To investigate whether the association between p33 and SNX-BAR proteins is direct or indirect, MBP-p33, GST-Vps5, GST-AtSnx1 and GST-AtSnx2b were obtained from *E*.*coli* (BL21 DE3 Codon Plus cells) [70]. First, we incubated 2 μg MBP-p33 with amylose resin (New England Biolabs, Cat#E8021L) at 4°C for 2 h. The same amount of MBP protein was used as the control. The unbound proteins were removed by repeated washing, followed by adding 1 μg GST-Vps5, GST-AtSnx1 and GST-AtSnx2b, respectively, onto amylose resin and rotating at 4°C for 4 h. The bound proteins were eluted with maltose elution buffer, and the co-purified proteins were detected by Western blotting [71].

### Analysis of viral replication in yeast expressing Ago1 and Dcr1

To measure if Vps5 contributes to the protection of the viral RNA, we co-expressed Dcr1 and Ago1 proteins from *S*. *castellii* as an intracellular RNAi probe in yeast as described [22]. Details can be found in S1 Text.

### *In vitro* cell-free extract-based replicase reconstitution assays

CFEs were prepared from untransformed BY4741, vps5Δ, vps5Δvps17Δ, vps34Δ and ymr1Δ yeast strains as described previously [15,22,49]. Reaction mixture for the *in vitro* replication contained 2 μl of CFE, 0.15 μg DI-72 (+)RNA, 400 ng affinity-purified MBP-p33, 400 ng affinity-purified MBP-p92^pol^ in 20 μl total volume. 0.1 U/μl micrococcal nuclease (MNase, Amersham Biosciences, Cat#E70196Y) was added to the reactions at different time points as shown in Figures, followed by incubation for 15 min at 25°C, then, 2.5 mM EGTA was added to the samples to inactivate the MNase. The CFE reactions were further incubated for a total of 3 h at 25°C.

The second *in vitro* replicase reconstitution assay utilized the CFE from wild-type yeast was pre-treated with purified Ymr1 PI(3)P phosphatase at 25°C for 50 min [25], then 0.15 μg DI-72 (+)RNA, 400 ng affinity-purified MBP-p33, 400 ng affinity-purified MBP-p92^pol^ were added to the reaction mixture in 20 μl total volume, 0.1 U/μl MNase was added to the reactions at different time points as shown in Figures, the reaction mixtures were incubated for 15 min at 25°C, then, 2.5 mM EGTA was added to the samples to inactivate the MNase. The CFE reactions were further incubated for a total of 3 h at 25°C.

The third *in vitro* replicase reconstitution assay utilized BY4741, vps5Δ and vps5Δvps17Δ yeast strains, which were transformed with pGAD-CUP1-His-p92 and pGBK-CUP1-His-p33. Expression of p33 and p92^pol^ was induced by adding 50 μM CuSO_4_ for 40 min to the medium. CFEs were separately prepared from the above yeasts. The CFE-based reaction mixtures were programmed with 0.5 μg DI-72 (+)RNA transcripts as described previously [22,49]. 0.1 U/μl MNase was added to the reactions at 20 min post incubation. The reaction mixtures were incubated for 15 min at 25°C, then, 2.5 mM EGTA was added to the samples to inactivate the MNase. The CFE-based replication mixtures were incubated at 25°C for a total of 3 h.

The fourth *in vitro* assay was based on yeast strains BY4741 and vps5Δ, which were transformed with pGAD-CUP1-His-p92 and pGBK-CUP1-His-p33-GAL1-DI72. CFEs were separately prepared from the above yeasts. MNase treatment was performed as describe above. The CFE-based replication mixtures were incubated at 25°C for a total of 3 h.

The total RNAs were isolated from the CFE-based reactions, the ^32^P-labeled RNA products were separated in 5% semi-denaturing polyacrylamide gel containing 8 M urea [22,49].

### Protein-RNA interaction between SNX-BAR proteins and viral RNA in plant and yeast cells

To investigate the interaction between Vps5 SNX-BAR protein and viral RNA within viral replicase complex in yeast, MS2-tagged RNA affinity purification assay was performed [72]. pGAD-Cup1-Flagp92, pGBK-Cup1-Flagp33, pESC-URA-Vps5-3xHA with pESC-TRP-repRNA or pESC-TRP-repRNA(+)MS2hp or pESC-TRP-repRNA(-)MS2hp were transformed into yeast SC1 strain. The transformed yeasts were pre-grown in synthetic complete medium (ULHT^-^) supplemented with 2% glucose and 100 μM BCS at 29°C overnight. This was followed by transferring the yeasts to synthetic complete medium (ULHT^-^) supplemented with 2% galactose and 100 μM BCS at 29°C for 24 h. Tombusviral repRNA replication was induced by changing the media to synthetic complete medium (ULHT^-^) supplemented with 2% galactose and 50 μM CuSO_4_ for 12 h at 23°C. The tombusviral repRNA replication in yeasts was confirmed by Northern blotting. To do the RNA-based purification, yeast cells were broken with glass beads, membrane fraction was solubilized with 1% Triton X-100 followed by incubation with GST resin coated with GST-MS2-CP (purified from *E*. *coli*), and then, the GST resins were washed five times. The co-purified proteins were detected by anti-HA and anti-Flag antibody for Vps5-3xHA and Flag-p33, respectively.

To test if SNX-BAR proteins associate with viral RNAs within the viral replicase complex in plants, trimolecular fluorescence complementation (TriFC) was conducted in *N*. *benthamiana* [73,74]. MS2-CP binding RNA hairpin sequence (MS2hp) was fused with viral repRNA to get the pGD-35S-repRNA(+)MS2hp and pGD-35S-repRNA(-)MS2hp constructs [75]. MS2-CP ORF was inserted into pGD-cYFP to make pGD-MS2-CP-cYFP plasmid. All the plasmids were transformed into *Agrobacteria* C58C1 strain. pGD-MS2-CP-cYFP (0.1 OD_600_), pGD-nYFP-Snx1 (0.1 OD_600_), pGD-p33-BFP (0.3 OD_600_), pGD-RFP-SKL (0.2 OD_600_), pGD-p33 (0.3 OD_600_), pGD-p92 (0.3 OD_600_) and pGD-repRNA (0.3 OD_600_) or pGD-35S-repRNA(+)MS2hp (0.3 OD_600_) or pGD-35S-repRNA(-)MS2hp (0.3 OD_600_) were co-agroinfiltrated into *N*. *benthamiana*. The plant leaves were studied by confocal microscopy 60 h post agroinfiltration, using confocal laser scanning microscope.

An RNA-based immuno-precipitation method was used to confirm the interaction between SNX-BAR proteins and viral RNA within the viral replicase complex *in planta* [76,77]. Agrobacterium harboring pGD-AtSnx1-Flag (0.3 OD_600_) or pGD-AtSnx2b-Flag (0.3 OD_600_) were infiltrated into in *N*. *benthamiana* leaves, and in parallel, pGD-AtSnx1-Flag (0.3 OD_600_) and pGD-AtSnx2b-Flag (0.3 OD_600_) were co-agroinfiltrated into plant leaves. pGD empty vector served as the negative control. TBSV sap inoculation was done 12 h post agroinfiltration. The leaf strips from the agroinfiltrated leaves (2 dpi) were cross-linked with 1% formaldehyde at 23°C for 1 h, then formaldehyde was quenched by adding 0.1 M glycine. Afterwards, the plant leaf strips were washed in 1xPBS buffer. The membrane fraction was isolated and solubilized with 1% Triton X-100. Flag-based purification of AtSnx1-Flag or AtSnx2b-Flag was performed with anti-Flag M2 agarose. The purified protein was detected with Western blot using anti-Flag antibody. Total RNAs were extracted from the purified protein-RNA complexes by phenol/chloroform, and then, cDNAs were synthesized for the detection of the co-purified viral genomic RNAs by RT-PCR. The PCR reactions were conducted with the same amount of samples and using the same number of PCR cycles for detecting the viral (+)gRNA and (-)gRNA, respectively [21].

## Supporting information

S1 Text Supplementary material and methods. (DOCX)

S1 Table Yeast PI3P binding proteins screened in this study. (DOCX)

S2 Table Amino acid sequence comparison of the BAR-domains of SNX-BAR proteins. (PDF)

S3 Table Amino acid sequence comparison of the PX-domains of SNX-BAR proteins. (PDF)

S4 Table Nucleotide sequence comparison of AtSnx2a and AtSnx2b. (DOCX)

S5 Table List of primers and plasmid constructs used in this study. (DOCX)

S1 Fig Pro-viral roles of the endosomal SNX-BAR proteins in tombusvirus replication in yeast and plant protoplasts. (A) Top image: Northern blot analysis shows decreased CIRV (+)repRNA accumulation in *vps5Δ* yeast strain. Vps5p and its deletion mutants were expressed from the constitutive *TEF1* promoter from a plasmid. The accumulation level of repRNA was normalized based on 18S rRNA levels (second panel). The accumulation of Strep-p36, Strep-p95 and Vps5-Flag is measured by western blotting and anti-Strep or anti-Flag antibodies. See further details in Fig 1A. (B) Top image: Northern blot analysis shows decreased TBSV (+)repRNA accumulation in *vps17Δ* yeast strain. Vps17p SNX-BAR protein was expressed from the *TEF1* promoter from a plasmid. The accumulation level of repRNA was normalized based on 18S rRNA levels (second panel). (C-D-E) VIGS-based knock-down of both SNX1 and SNX2b mRNA levels inhibits the accumulation of TBSV, CIRV and the related TCV RNAs in *N*. *benthamiana* protoplasts. Top panel: The accumulation of TBSV, CIRV and TCV gRNA and sgRNAs was measured using Northern blot analysis of total RNA samples obtained from *N*. *benthamiana* protoplasts. Second panel: ethidium-bromide stained gels show ribosomal RNA level. Protoplasts were isolated from the upper leaves of SNX1/SNX2b-silenced *N*. *benthamiana* on the 12th day, followed by transformation of TBSV, CIRV and TCV RNA, respectively. Twenty-four hours later, total RNA was analyzed by Northern blotting. (F) Up-regulation of Snx1 and Snx2b expression in TBSV and CIRV-infected *N*. *benthamiana* leaves. The mRNA levels for the SNX-BAR proteins were estimated by semi-quantitative RT-PCR in total RNA samples obtained from TBSV or CIRV-infected versus mock-infected *N*. *benthamiana* leaves. Tubulin mRNA and ribosomal RNA were used as controls (bottom panels). (G-H) The endosomal Vps5p SNX-BAR protein is not required for nodavirus replication in yeast. Top image: Northern blot analysis shows the accumulation level of NoV and FHV (+)RNA1 and the subgenomic RNA3 in *vps5Δ* yeast strain. The accumulation level of repRNA was normalized based on 18S rRNA levels (second panel). Bottom panel: The flag-tagged Protein A replication protein expression was measured with western blotting using anti-Flag antibody.
(TIF)

S2 Fig The TBSV and CIRV replication proteins interact with the endosomal SNX-BAR proteins in plant cells. (A) Interaction between TBSV p92^pol^ replication protein and the SNX-BAR proteins was detected by BiFC assay *in planta*. TBSV p92-cYFP and the nYFP-Snx2b, nYFP-Snx2a or nYFP-Snx1 proteins were co-expressed from the 35S promoter after co-agroinfiltration into *N*. *benthamiana* leaves. Bottom three panels: negative control was GST-cYFP and the SNX-BAR proteins analyzed by BiFC assay *in planta*. Note that the plants were infected with TBSV to induce the viral replication compartments in cells. Co-localization of RFP-SKL peroxisomal luminar marker with the BiFC signals demonstrates that the interaction between p92^pol^ and SNX-BAR proteins occurs in the viral replication compartments. Scale bars represent 10 μm. (B) Western blots show the accumulation levels of the proteins used in the BiFC assays. Top panel for S2A Fig, bottom panels for Fig 3A. (C) Interactions between CIRV p36 replication protein and the SNX-BAR proteins were detected by BiFC assay *in planta*. CIRV p36-cYFP and nYFP-Snx2b, nYFP-Snx2a or nYFP-Snx1 proteins were co-expressed from the 35S promoter after co-agroinfiltration into *N*. *benthamiana* leaves. nYFP-GST was co-expressed with p36-cYFP to serve as the negative control for BiFC assay. Note that the plants were infected with CIRV to induce the viral replication compartments in cells. Scale bars represent 10 μm.
(TIF)

S3 Fig Recruitment of the endosomal SNX-BAR proteins into the viral replication compartments by tombusviral replication proteins in yeast and plant cells. (A-B) Co-localization of TBSV p33-BFP with the GFP-tagged Snx1 and GFP-tagged Snx2b in *N*. *benthamiana* cells is detected by confocal laser microscopy. Scale bars represent 5 μm. (C) Localization pattern of Snx1-GFP in the absence of viral components in *N*. *benthamiana* cells is detected by confocal laser microscopy. (D) Co-localization of TBSV GFP-p33 and CIRV GFP-p36, respectively, with Vps5-Flag SNX-BAR protein in yeast cells replicating repRNA. Vps5-Flag was detected with anti-Flag antibody. Each experiment was repeated three times. (E) Absent of excessive co-localization of TBSV GFP-p33 with Vps5yr-AA-Flag mutant protein in yeast cells replicating repRNA.
(TIF)

S4 Fig Both PI(3)P phosphoinositide and endosomal SNX-BAR proteins are co-opted into the viral replication compartments in plant cells. (A) Co-localization of TBSV p33-BFP with the GFP-tagged Snx1 and with RFP-2xFYVE protein in *N*. *benthamiana* leaf tissues and protoplasts is detected by confocal laser microscopy. RFP-2xFYVE protein binds PI(3)P selectively. Top two panels: Co-localization of TBSV p33-BFP with the GFP-tagged Snx1 and with RFP-2xFYVE protein in *N*. *benthamiana* leaves. Middle panel represents images in the absence of viral components. Scale bars represent 10 μm. Bottom panel shows the co-localization of TBSV p33-BFP with the GFP-tagged Snx1 and with RFP-2xFYVE protein in *N*. *benthamiana* protoplasts. Scale bars represent 20 μm. (B) Co-localization of TBSV p33-BFP with the GFP-tagged Snx2b and with RFP-2xFYVE protein in *N*. *benthamiana* leaf tissues and protoplasts is detected by confocal laser microscopy. Top panel: Co-localization of TBSV p33-BFP with the GFP-tagged Snx1 and with RFP-2xFYVE protein in *N*. *benthamiana* leaves. Second panel represents images in the absence of viral components. Scale bars represent 10 μm. Third panel shows the co-localization of TBSV p33-BFP with the GFP-tagged Snx2b and with RFP-2xFYVE protein in *N*. *benthamiana* protoplasts. Bottom panel represents images in the absence of viral components. Scale bars represent 20 μm.
(TIF)

S5 Fig PI(3)P phosphoinositide is required for recruitment of Snx1 protein into the viral replication compartments in plant cells. (A-B) BiFC assay shows the reduced level of interaction between p33 replication protein and the Snx1 protein in *N*. *benthamiana* treated with Wortmannin, a Vps34 PI3K inhibitor, or with DMSO as a negative control. TBSV p33-cYFP and nYFP-Snx1 proteins were co-expressed from the 35S promoter after co-agroinfiltration into *N*. *benthamiana* leaves. RFP-SKL was expressed as a peroxisomal marker to identify the viral replication compartments. Scale bars represent 10 μm. The BiFC signals were quantified via Image J. Each experiment was repeated. (C) Western blot analysis shows the accumulation level of nYFP-Snx1 and nYFP-Snx2b proteins and p33-cYFP in Wortmannin or DMSO-treated *N*. *benthamiana* leaves. (D) BiFC assay shows that nYFP-Snx1^rry^ protein was not recruited by CIRV p36-cYFP replication protein into the viral replication compartment in *N*. *benthamiana* infected with CIRV. The CIRV replication compartment was decorated with CoxIV-RFP mitochondria marker protein. Scale bars represent 10 μm. (E) Pull-down assay shows direct interaction of TBSV p33 replication protein with the shown Snx1 mutants *in vitro*. Top panel: Western blot analysis of the captured GST-Snx1 mutants with the MBP-affinity purified p33 (lanes 1, 3, 5 and 7). The negative control was MBP (lanes 2, 4, 6 and 8). Bottom panel: The captured MBP-p33 and MBP were detected with anti-MBP antibody. Note that equal amount of each GST fusion protein was incubated with MBP-p33 or MBP. (F) Expression of RavZ^CT^-p33 fusion protein inhibits TBSV replication in yeast. Reduced repRNA accumulation by expression of RavZ^CT^-p33 fusion protein in comparison with p33 replication protein in wt yeast replicating TBSV repRNA. RavZ^CT^-p33 fusion protein lacking the PI(3)P-binding domain (i.e., RavZ^CT-P^) loses the strong inhibitory effect on TBSV replication. Top panel shows the northern blot analysis of (+)repRNA accumulation. Middle panel: the 18S rRNA level. Accumulation of p33 and the fusion proteins was shown in bottom panel. (G) Co-expression of RavZ^CT^ or RavZ^CT-P^ with viral p33, p92 and repRNA does not affect TBSV replication in wt yeast. The accumulation of repRNA was normalized based on 18S rRNA levels (second panel). The protein level of RavZ^CT^ or RavZ^CT-P^ was shown in bottom panel.
(TIF)

S6 Fig Recruitment of retromer proteins and the endosomal SNX-BAR proteins into VROs by tombusviral replication proteins in plant cells. (A) Co-localization of TBSV p33-BFP with the GFP-tagged retromer proteins (Vps26, Vps35 and Vps29) and RFP-tagged Snx2b in *N*. *benthamiana* cells is detected by confocal laser microscopy. The plants were infected with TBSV. Scale bars represent 10 μm. (B) Different co-localization pattern of the GFP-tagged retromer proteins (Vps26, Vps35 and Vps29) and RFP-tagged Snx2b in the absence of viral components in *N*. *benthamiana* cells is detected by confocal laser microscopy. Scale bars represent 10 μm.
(TIF)

S7 Fig Vps5 SNX-BAR protein and PI(3)P are required for TBSV replication and protection of the viral dsRNA in vitro. (A-B) Reduced repRNA production by the tombusvirus replicase assembled *in vps5Δ* yeast strain. The yeast strains expressed the p33 and p92^pol^ replication proteins and (+)repRNA, allowing for the assembly of the viral replicase complex in yeast peroxisomal membranes. The CFEs were treated with MNase 20 min latter, followed by the inactivation of MNase after 15 min with EGTA. Non-denaturing PAGE analysis shows the ^32^P-labeled TBSV repRNA products from the *in vitro* assay. (C-D) *In vitro* reconstitution of the TBSV replicase using purified recombinant p33 and p92^pol^ replication proteins and TBSV (+)repRNA. The CFEs were prepared from wt yeast and were pre-incubated with purified recombinant Flag-tagged Ymr1 PI(3)P phosphatase to reduce the PI(3)P level in the CFE. The MNase treatments, which lasted for 15 min, were done at three different time points as shown. Non-denaturing PAGE analysis was done as in Fig 5. (E-F) In vitro association of Vps5 or Snx1 SNX-BAR proteins with the viral RNAs. In vitro RNA gel mobility shift assay shows that GST-Vps5 or GST-Snx1 bind to the ^32^P-labeled (+)repRNA and (-)repRNA, respectively. Purified GST-Vps5, GST-Snx1 or GST was added in increasing amounts (1, 3, 6, 15 and 30 pMol for GST, 1, 3 and 6 pmol for GST-Vps5, and 6, 15 and 30 pmol for GST-Snx1) to the assays. The Vps5-repRNA or Snx1-repRNA complex was analyzed on non-denaturing 5% polyacrylamide gels. Each experiment was repeated. (G) Scheme of the MS2-CP-based RNA purification assay. Flag-p33, Flag-p92 and Vps5-3xHA and the repRNA derivatives were expressed in yeast to allow the formation of RNA-protein complexes. This scheme is to explain the experiments presented in Fig 7.
(TIF)

## References

[1] Fernandez de Castro I, Tenorio R, Risco C (2016) Virus assembly factories in a lipid world. Curr Opin Virol 18: 20–26. 10.1016/j.coviro.2016.02.009 26985879

[2] Wang A (2015) Dissecting the molecular network of virus-plant interactions: the complex roles of host factors. Annu Rev Phytopathol 53: 45–66. 10.1146/annurev-phyto-080614-120001 25938276

[3] Paul D, Bartenschlager R (2015) Flaviviridae Replication Organelles: Oh, What a Tangled Web We Weave. Annu Rev Virol 2: 289–310. 10.1146/annurev-virology-100114-055007 26958917

[4] Nagy PD, Pogany J (2012) The dependence of viral RNA replication on co-opted host factors. Nature Reviews Microbiology 10: 137–149. 10.1038/nrmicro2692 PMC7097227 22183253

[5] Shulla A, Randall G (2016) (+) RNA virus replication compartments: a safe home for (most) viral replication. Curr Opin Microbiol 32: 82–88. 10.1016/j.mib.2016.05.003 27253151 PMC4983521

[6] Fernandez de Castro I, Fernandez JJ, Barajas D, Nagy PD, Risco C (2017) Three-dimensional imaging of the intracellular assembly of a functional viral RNA replicase complex. J Cell Sci 130: 260–268. 10.1242/jcs.181586 27026525

[7] Harak C, Lohmann V (2015) Ultrastructure of the replication sites of positive-strand RNA viruses. Virology 479–480: 418–433. 10.1016/j.virol.2015.02.029 25746936 PMC7111692

[8] Ertel KJ, Benefield D, Castano-Diez D, Pennington JG, Horswill M, den Boon JA, et al (2017) Cryo-electron tomography reveals novel features of a viral RNA replication compartment. Elife 6 10.7554/eLife.25940 28653620 PMC5515581

[9] Nagy PD (2017) Exploitation of a surrogate host, Saccharomyces cerevisiae, to identify cellular targets and develop novel antiviral approaches. Curr Opin Virol 26: 132–140. 10.1016/j.coviro.2017.07.031 28843111

[10] Nagy PD (2016) Tombusvirus-Host Interactions: Co-Opted Evolutionarily Conserved Host Factors Take Center Court. Annu Rev Virol 3: 491–515. 10.1146/annurev-virology-110615-042312 27578441

[11] White KA, Nagy PD (2004) Advances in the molecular biology of tombusviruses: gene expression, genome replication, and recombination. Prog Nucleic Acid Res Mol Biol 78: 187–226. 10.1016/S0079-6603(04)78005-8 15210331

[12] Stork J, Kovalev N, Sasvari Z, Nagy PD (2011) RNA chaperone activity of the tombusviral p33 replication protein facilitates initiation of RNA synthesis by the viral RdRp in vitro. Virology 409: 338–347. 10.1016/j.virol.2010.10.015 21071052 PMC7173327

[13] Pogany J, White KA, Nagy PD (2005) Specific binding of tombusvirus replication protein p33 to an internal replication element in the viral RNA is essential for replication. J Virol 79: 4859–4869. 10.1128/JVI.79.8.4859-4869.2005 15795271 PMC1069559

[14] Panavas T, Nagy PD (2003) Yeast as a model host to study replication and recombination of defective interfering RNA of Tomato bushy stunt virus. Virology 314: 315–325. 10.1016/s0042-6822(03)00436-7 14517084

[15] Nagy PD, Pogany J, Xu K (2016) Cell-Free and Cell-Based Approaches to Explore the Roles of Host Membranes and Lipids in the Formation of Viral Replication Compartment Induced by Tombusviruses. Viruses 8 10.3390/v8030068 26950140 PMC4810258

[16] Sasvari Z, Lin W, Inaba JI, Xu K, Kovalev N, Nagy PD (2020) Co-opted Cellular Sac1 Lipid Phosphatase and PI(4)P Phosphoinositide Are Key Host Factors during the Biogenesis of the Tombusvirus Replication Compartment. J Virol 94 10.1128/JVI.01979-19 32269127 PMC7307105

[17] Sasvari Z, Kovalev N, Gonzalez PA, Xu K, Nagy PD (2018) Assembly-hub function of ER-localized SNARE proteins in biogenesis of tombusvirus replication compartment. PLoS Pathog 14: e1007028 10.1371/journal.ppat.1007028 29746582 PMC5963807

[18] Inaba JI, Xu K, Kovalev N, Ramanathan H, Roy CR, Lindenbach BD et al (2019) Screening Legionella effectors for antiviral effects reveals Rab1 GTPase as a proviral factor coopted for tombusvirus replication. Proc Natl Acad Sci U S A 116: 21739–21747. 10.1073/pnas.1911108116 31591191 PMC6815150

[19] Jonczyk M, Pathak KB, Sharma M, Nagy PD (2007) Exploiting alternative subcellular location for replication: tombusvirus replication switches to the endoplasmic reticulum in the absence of peroxisomes. Virology 362: 320–330. 10.1016/j.virol.2007.01.004 17292435

[20] Rochon D, Singh B, Reade R, Theilmann J, Ghoshal K, Alam SB et al (2014) The p33 auxiliary replicase protein of Cucumber necrosis virus targets peroxisomes and infection induces de novo peroxisome formation from the endoplasmic reticulum. Virology 452–453: 133–142. 10.1016/j.virol.2013.12.035 24606690

[21] Barajas D, Martin IF, Pogany J, Risco C, Nagy PD (2014) Noncanonical role for the host Vps4 AAA+ ATPase ESCRT protein in the formation of Tomato bushy stunt virus replicase. PLoS Pathog 10: e1004087 10.1371/journal.ppat.1004087 24763736 PMC3999190

[22] Kovalev N, Inaba JI, Li Z, Nagy PD (2017) The role of co-opted ESCRT proteins and lipid factors in protection of tombusviral double-stranded RNA replication intermediate against reconstituted RNAi in yeast. PLoS Pathog 13: e1006520 10.1371/journal.ppat.1006520 28759634 PMC5552349

[23] Barajas D, Xu K, de Castro Martin IF, Sasvari Z, Brandizzi F, Risco C et al (2014) Co-opted Oxysterol-Binding ORP and VAP Proteins Channel Sterols to RNA Virus Replication Sites via Membrane Contact Sites. PLoS Pathog 10: e1004388 10.1371/journal.ppat.1004388 25329172 PMC4199759

[24] Xu K, Nagy PD (2016) Enrichment of Phosphatidylethanolamine in Viral Replication Compartments via Co-opting the Endosomal Rab5 Small GTPase by a Positive-Strand RNA Virus. PLoS Biol 14: e2000128 10.1371/journal.pbio.2000128 27760128 PMC5070881

[25] Feng Z, Xu K, Kovalev N, Nagy PD (2019) Recruitment of Vps34 PI3K and enrichment of PI3P phosphoinositide in the viral replication compartment is crucial for replication of a positive-strand RNA virus. PLoS Pathog 15: e1007530 10.1371/journal.ppat.1007530 30625229 PMC6342326

[26] Balla T (2013) Phosphoinositides: tiny lipids with giant impact on cell regulation. Physiol Rev 93: 1019–1137. 10.1152/physrev.00028.2012 23899561 PMC3962547

[27] Jiang RH, Stahelin RV, Bhattacharjee S, Haldar K (2013) Eukaryotic virulence determinants utilize phosphoinositides at the ER and host cell surface. Trends Microbiol 21: 145–156. 10.1016/j.tim.2012.12.004 23375057 PMC3595378

[28] Xu K, Nagy PD (2015) RNA virus replication depends on enrichment of phosphatidylethanolamine at replication sites in subcellular membranes. Proc Natl Acad Sci U S A 112: E1782–E1791. 10.1073/pnas.1418971112 25810252 PMC4394249

[29] Kovtun O, Leneva N, Bykov YS, Ariotti N, Teasdale RD, Schaffer M et al (2018) Structure of the membrane-assembled retromer coat determined by cryo-electron tomography. Nature 561: 561–564. 10.1038/s41586-018-0526-z 30224749 PMC6173284

[30] Chandra M, Collins BM (2018) The Phox Homology (PX) Domain. Adv Exp Med Biol. 10.1007/5584_2018_185 29569114

[31] van Weering JR, Cullen PJ (2014) Membrane-associated cargo recycling by tubule-based endosomal sorting. Semin Cell Dev Biol 31: 40–47. 10.1016/j.semcdb.2014.03.015 24641888

[32] Wang J, Fedoseienko A, Chen B, Burstein E, Jia D, Billadeau DD (2018) Endosomal receptor trafficking: Retromer and beyond. Traffic 19: 578–590. 10.1111/tra.12574 29667289 PMC6043395

[33] Cullen PJ, Steinberg F (2018) To degrade or not to degrade: mechanisms and significance of endocytic recycling. Nat Rev Mol Cell Biol 19: 679–696. 10.1038/s41580-018-0053-7 30194414

[34] Reitz C (2018) Retromer Dysfunction and Neurodegenerative Disease. Curr Genomics 19: 279–288. 10.2174/1389202919666171024122809 29755290 PMC5930449

[35] Maschkowitz G, Gartner S, Hofmann-Winkler H, Fickenscher H, Winkler M (2018) Interaction of Human Cytomegalovirus Tegument Proteins ppUL35 and ppUL35A with Sorting Nexin 5 Regulates Glycoprotein B (gpUL55) Localization. J Virol 92 10.1128/JVI.00013-18 29444945 PMC5899209

[36] Bergant Marusic M, Ozbun MA, Campos SK, Myers MP, Banks L (2012) Human papillomavirus L2 facilitates viral escape from late endosomes via sorting nexin 17. Traffic 13: 455–467. 10.1111/j.1600-0854.2011.01320.x 22151726 PMC3276720

[37] Wei J, Lian H, Guo W, Chen YD, Zhang XN, Zang R et al (2018) SNX8 modulates innate immune response to DNA virus by mediating trafficking and activation of MITA. PLoS Pathog 14: e1007336 10.1371/journal.ppat.1007336 30321235 PMC6188873

[38] Chuang C, Prasanth KR, Nagy PD (2015) Coordinated function of cellular DEAD-box helicases in suppression of viral RNA recombination and maintenance of viral genome integrity. PLoS Pathog 11: e1004680 10.1371/journal.ppat.1004680 25693185 PMC4333740

[39] Serva S, Nagy PD (2006) Proteomics analysis of the tombusvirus replicase: Hsp70 molecular chaperone is associated with the replicase and enhances viral RNA replication. J Virol 80: 2162–2169. 10.1128/JVI.80.5.2162-2169.2006 16474124 PMC1395393

[40] Prasanth KR, Chuang C, Nagy PD (2017) Co-opting ATP-generating glycolytic enzyme PGK1 phosphoglycerate kinase facilitates the assembly of viral replicase complexes. PLoS Pathog 13: e1006689 10.1371/journal.ppat.1006689 29059239 PMC5695612

[41] Le Floc'h A, Tanaka Y, Bantilan NS, Voisinne G, Altan-Bonnet G, Fukui Y et al (2013) Annular PIP3 accumulation controls actin architecture and modulates cytotoxicity at the immunological synapse. J Exp Med 210: 2721–2737. 10.1084/jem.20131324 24190432 PMC3832928

[42] Heucken N, Ivanov R (2018) The retromer, sorting nexins and the plant endomembrane protein trafficking. J Cell Sci 131 10.1242/jcs.203695 29061884

[43] Gallon M, Cullen PJ (2015) Retromer and sorting nexins in endosomal sorting. Biochem Soc Trans 43: 33–47. 10.1042/BST20140290 25619244

[44] Burda P, Padilla SM, Sarkar S, Emr SD (2002) Retromer function in endosome-to-Golgi retrograde transport is regulated by the yeast Vps34 PtdIns 3-kinase. J Cell Sci 115: 3889–3900. 10.1242/jcs.00090 12244127

[45] Lenoir M, Ustunel C, Rajesh S, Kaur J, Moreau D, Gruenberg J et al (2018) Phosphorylation of conserved phosphoinositide binding pocket regulates sorting nexin membrane targeting. Nat Commun 9: 993 10.1038/s41467-018-03370-1 29520003 PMC5843628

[46] Horenkamp FA, Kauffman KJ, Kohler LJ, Sherwood RK, Krueger KP, Shteyn V et al (2015) The Legionella Anti-autophagy Effector RavZ Targets the Autophagosome via PI3P- and Curvature-Sensing Motifs. Dev Cell 34: 569–576. 10.1016/j.devcel.2015.08.010 26343456 PMC4594837

[47] Burd C, Cullen PJ (2014) Retromer: a master conductor of endosome sorting. Cold Spring Harb Perspect Biol 6 10.1101/cshperspect.a016774 24492709 PMC3941235

[48] van Weering JR, Verkade P, Cullen PJ (2012) SNX-BAR-mediated endosome tubulation is co-ordinated with endosome maturation. Traffic 13: 94–107. 10.1111/j.1600-0854.2011.01297.x 21973056

[49] Pogany J, Stork J, Li Z, Nagy PD (2008) In vitro assembly of the Tomato bushy stunt virus replicase requires the host Heat shock protein 70. Proc Natl Acad Sci U S A 105: 19956–19961. 10.1073/pnas.0810851105 19060219 PMC2604936

[50] Pogany J, Nagy PD (2008) Authentic replication and recombination of Tomato bushy stunt virus RNA in a cell-free extract from yeast. J Virol 82: 5967–5980. 10.1128/JVI.02737-07 18417594 PMC2395147

[51] Kovalev N, Pogany J, Nagy PD (2014) Template role of double-stranded RNA in tombusvirus replication. J Virol 88: 5638–5651. 10.1128/JVI.03842-13 24600009 PMC4019106

[52] Xu K, Huang TS, Nagy PD (2012) Authentic in vitro replication of two tombusviruses in isolated mitochondrial and endoplasmic reticulum membranes. J Virol 86: 12779–12794. 10.1128/JVI.00973-12 22973028 PMC3497632

[53] Kovalev N, Pogany J, Nagy PD (2020) Reconstitution of an RNA virus replicase in artificial giant unilamellar vesicles supports full replication and provides protection for the dsRNA replication intermediate. J Virol. 10.1128/JVI.00267-20 32641477 PMC7459549

[54] Parrish WR, Stefan CJ, Emr SD (2004) Essential role for the myotubularin-related phosphatase Ymr1p and the synaptojanin-like phosphatases Sjl2p and Sjl3p in regulation of phosphatidylinositol 3-phosphate in yeast. Mol Biol Cell 15: 3567–3579. 10.1091/mbc.e04-03-0209 15169871 PMC491819

[55] Drinnenberg IA, Weinberg DE, Xie KT, Mower JP, Wolfe KH, Fink GR et al (2009) RNAi in budding yeast. Science 326: 544–550. 10.1126/science.1176945 19745116 PMC3786161

[56] Bertrand E, Chartrand P, Schaefer M, Shenoy SM, Singer RH, Long RM (1998) Localization of ASH1 mRNA particles in living yeast. Mol Cell 2: 437–445. 10.1016/s1097-2765(00)80143-4 9809065

[57] Richardson LG, Clendening EA, Sheen H, Gidda SK, White KA, Mullen RT (2014) A unique N-terminal sequence in the Carnation Italian ringspot virus p36 replicase-associated protein interacts with the host cell ESCRT-I component Vps23. J Virol 88: 6329–6344. 10.1128/JVI.03840-13 24672030 PMC4093892

[58] Zhang Z, He G, Filipowicz NA, Randall G, Belov GA, Kopek BG et al (2019) Host Lipids in Positive-Strand RNA Virus Genome Replication. Front Microbiol 10: 286 10.3389/fmicb.2019.00286 30863375 PMC6399474

[59] Neuvonen M, Kazlauskas A, Martikainen M, Hinkkanen A, Ahola T, Saksela K (2011) SH3 domain-mediated recruitment of host cell amphiphysins by alphavirus nsP3 promotes viral RNA replication. PLoS Pathog 7: e1002383 10.1371/journal.ppat.1002383 22114558 PMC3219718

[60] Diaz A, Wang X, Ahlquist P (2010) Membrane-shaping host reticulon proteins play crucial roles in viral RNA replication compartment formation and function. Proc Natl Acad Sci U S A 107: 16291–16296. 10.1073/pnas.1011105107 20805477 PMC2941330

[61] Aktepe TE, Liebscher S, Prier JE, Simmons CP, Mackenzie JM (2017) The Host Protein Reticulon 3.1A Is Utilized by Flaviviruses to Facilitate Membrane Remodelling. Cell Rep 21: 1639–1654. 10.1016/j.celrep.2017.10.055 29117567

[62] Chao TC, Su WC, Huang JY, Chen YC, Jeng KS, Wang HD et al (2012) Proline-serine-threonine phosphatase-interacting protein 2 (PSTPIP2), a host membrane-deforming protein, is critical for membranous web formation in hepatitis C virus replication. J Virol 86: 1739–1749. 10.1128/JVI.06001-11 22130530 PMC3264356

[63] Popov S, Popova E, Inoue M, Wu Y, Gottlinger H (2018) HIV-1 gag recruits PACSIN2 to promote virus spreading. Proc Natl Acad Sci U S A 115: 7093–7098. 10.1073/pnas.1801849115 29891700 PMC6142272

[64] Janke C, Magiera MM, Rathfelder N, Taxis C, Reber S, Maekawa H et al (2004) A versatile toolbox for PCR-based tagging of yeast genes: new fluorescent proteins, more markers and promoter substitution cassettes. Yeast 21: 947–962. 10.1002/yea.1142 15334558

[65] Bachan S, Dinesh-Kumar SP (2012) Tobacco rattle virus (TRV)-based virus-induced gene silencing. Methods Mol Biol 894: 83–92. 10.1007/978-1-61779-882-5_6 22678574

[66] Jaag HM, Nagy PD (2009) Silencing of Nicotiana benthamiana Xrn4p exoribonuclease promotes tombusvirus RNA accumulation and recombination. Virology 386: 344–352. 10.1016/j.virol.2009.01.015 19232421

[67] Yoo SD, Cho YH, Sheen J (2007) Arabidopsis mesophyll protoplasts: a versatile cell system for transient gene expression analysis. Nat Protoc 2: 1565–1572. 10.1038/nprot.2007.199 17585298

[68] Panaviene Z, Baker JM, Nagy PD (2003) The overlapping RNA-binding domains of p33 and p92 replicase proteins are essential for tombusvirus replication. Virology 308: 191–205. 10.1016/s0042-6822(02)00132-0 12706102

[69] Nawaz-Ul-Rehman MS, Prasanth KR, Xu K, Sasvari Z, Kovalev N, de Castro Martin IF et al (2016) Viral Replication Protein Inhibits Cellular Cofilin Actin Depolymerization Factor to Regulate the Actin Network and Promote Viral Replicase Assembly. PLoS Pathog 12: e1005440 10.1371/journal.ppat.1005440 26863541 PMC4749184

[70] Rajendran KS, Nagy PD (2003) Characterization of the RNA-binding domains in the replicase proteins of tomato bushy stunt virus. J Virol 77: 9244–9258. 10.1128/jvi.77.17.9244-9258.2003 12915540 PMC187376

[71] Chuang C, Prasanth KR, Nagy PD (2017) The Glycolytic Pyruvate Kinase Is Recruited Directly into the Viral Replicase Complex to Generate ATP for RNA Synthesis. Cell Host Microbe 22: 639–652 e637. 10.1016/j.chom.2017.10.004 29107644

[72] Yoon JH, Gorospe M (2016) Identification of mRNA-Interacting Factors by MS2-TRAP (MS2-Tagged RNA Affinity Purification). Methods Mol Biol 1421: 15–22. 10.1007/978-1-4939-3591-8_2 26965253 PMC5140279

[73] Han Y, Wang S, Zhang Z, Ma X, Li W, Zhang X et al (2014) In vivo imaging of protein-protein and RNA-protein interactions using novel far-red fluorescence complementation systems. Nucleic Acids Res 42: e103 10.1093/nar/gku408 24813442 PMC4117741

[74] Seo JS, Sun HX, Park BS, Huang CH, Yeh SD, Jung C et al (2017) ELF18-INDUCED LONG-NONCODING RNA Associates with Mediator to Enhance Expression of Innate Immune Response Genes in Arabidopsis. Plant Cell 29: 1024–1038. 10.1105/tpc.16.00886 28400491 PMC5466027

[75] Wu CY, Nagy PD (2019) Blocking tombusvirus replication through the antiviral functions of DDX17-like RH30 DEAD-box helicase. PLoS Pathog 15: e1007771 10.1371/journal.ppat.1007771 31136641 PMC6555533

[76] Selth LA, Gilbert C, Svejstrup JQ (2009) RNA immunoprecipitation to determine RNA-protein associations in vivo. Cold Spring Harb Protoc 2009: pdb prot5234. 10.1101/pdb.prot5234 20147192

[77] Terzi LC, Simpson GG (2009) Arabidopsis RNA immunoprecipitation. Plant J 59: 163–168. 10.1111/j.1365-313X.2009.03859.x 19419533

