# Supplementary material for: Key interplay between the co-opted sorting nexin-BAR proteins and PI3P phosphoinositide in the formation of the tombusvirus replicase
Source: PLoS Pathog. 2020 Dec 28;16(12):e1009120. doi: 10.1371/journal.ppat.1009120 (PMC7833164; doi:10.1371/journal.ppat.1009120)
Supplement: S1 Text — (DOCX) [file ppat.1009120.s001.docx]

**S1 Text. Supplementary material and methods**

**Recombinant Protein Purification from *E. coli***

Recombinant MBP-tagged AtSnx2b was expressed in *E. coli* and purified as described previously [1,2]. Briefly, the expression plasmid pMAL-MBP-AtSnx2b was transformed into *E. coli* strain BL21 (DE3) CodonPlus. Protein expression was induced by adding 0.3 mM IPTG to the culture at 16°C for 12 h. After collection of the cultures by centrifugation at 4,000 g for 5 min, the cells were re-suspended and sonicated in reduced-salt column buffer (25 mM NaCl, 30 mM HEPES-KOH pH 7.4, 1 mM EDTA, 10 mM β-mercaptoethanol, 0.25 mg/ml RNase A). The lysate was centrifuged at 10,000 rpm for 15 min to remove cell debris. Then, supernatant was incubated with amylose resin (New England Biolabs, Cat#E8021L) at 4°C for 1 h. After washing the resin with 60 ml reduced-salt column buffer, the recombinant proteins were eluted with 10 mM maltose elution buffer. The affinity-purified proteins were analyzed by SDS-PAGE gel.

Recombinant GST-tagged Vps5, AtSnx1, AtSnx2b or MS2-CP was expressed in *E.coli* strain BL21 (DE3) CodonPlus. Protein expression was induced by adding 0.3 mM IPTG to the culture at 16°C for 12 h. After induction, cells were centrifuged and sonicated, centrifuged and the supernatant was incubated with GST resin (Merck Millipore, Cat#70541) at 4°C for 3 h. The GST-fusion proteins were eluted with 10 mM reduced glutathione at 4°C for 1 h.

**Western blot analysis**

Isolation of plant proteins and yeast proteins has been described previously [3]. Protein samples were loaded onto SDS-PAGE gel, followed by electrophoresis, transferred the protein to PVDF membranes. The membranes were incubated with primary anti-HA, anti-HIS, anti-FLAG or anti-MYC antibody, respectively. After the washing steps, membranes were incubated with secondary antibody. Finally, the membranes were developed using BCIP/NBT color development substrates or ECL western blotting substrate (GE Healthcare, Cat#RPN2232) [3].

**Preparation of GUVs and reconstitution of the TBSV replicase**

GUVs were prepared [4] using 1 mg/ml solutions of phospholipids in chloroform (Avanti, USA) using Vesicle Prep Pro (Nanion, Germany) workstation by electroformation. The protocol used was as follows [4]: Freq: 005.0, Ampl: 03.00, Temp: 050.0, Rise time: 05:00, Main time: 999:00, Fall time: 05:00. At the end of the protocol, sorbitol solution with GUVs was placed into an eppendorf tube and stored at 4°C for 1-2 weeks.

For the GUV-based CFE reaction, the procedure [5,6] was modified to remove the internal membranes from the CFEs (called S-CFE) via centrifugation for 20 min at 42,000 g at 4°C for the separation of the “soluble” (S-CFE, supernatant) and “membrane” (pellet) fractions. S-CFEs were prepared from BY4741 and vps5Δ yeast strains. The S-CFEs were added to the reaction mixtures containing 0.25 µg DI-72 (+)repRNA transcript, 200 ng purified recombinant MBP-p33, 200 ng purified recombinant MBP-p92^pol^, 30 mM HEPES-KOH, pH 7.4, 150 mM potassium acetate, 5 mM magnesium acetate, 0.13 M sorbitol, 0.4 µl actinomycin D (5 mg/ml), 2 µl of 150 mM creatine phosphate, 0.2 µl of 10 mg/ml creatine kinase, 0.2 µl of RNase inhibitor, 0.2 µl of 1 M dithiothreitol (DTT), 2 µl of rNTP mixture (10 mM ATP, CTP, and GTP and 0.25 mM UTP and ^32^P-UTP) and aliquoted GUVs. The reaction was performed at 25°C for 2.5 hours. To test the protection of dsRNA products by the above reconstituted viral replicases, 0.2 U of RNase III (NEB) was added to the reaction mixture 15 min prior to the extraction of RNA samples with phenol-chloroform and precipitation [7] The ^32^P-labeled RNA products synthesized in replication assay were loaded without heat treatment onto the 5% PAGE containing 8 M urea and separated by electrophoresis in 0.5x Tris-borate-EDTA (TBE) buffer.

**Electrophoretic mobility shift assay**

To test the interaction between SNX-BAR proteins and viral RNAs *in vitro*, GST-Vps5, GST-Snx1 and MBP-Snx2b were purified from *E. coli*, whereas viral full-length (+)repRNA and (-)repRNA were synthesized by *in vitro* T7 RNA polymerase-based transcription. Electrophoretic mobility shift assay was performed with different amount of purified proteins as shown in the legend for Figures. The incubation with viral repRNAs was in the RNA binding buffer [10 mM HEPES pH 7.4, 50 mM NaCl , 1 mM DTT, 1 mM EDTA, 5% Glycerol] at 25°C for 15 min [8]. Non-denaturing PAGE gel with 5% acrylamide was used to separate the protein-RNA complex from free RNAs.

**Analysis of nodavirus replication in yeast**

To test the effect of yeast Vps5 SNX-BAR protein in the replication of Flock House virus (FHV), yeast strains BY4741 and vps5Δ were transformed with plasmids pESC-His-Cup1-FHV-RNA1/frameshift/TRSV-RZ, pGAD-Leu-Cup1-FHVproteinA-HA-Flag and pESC-Ura. The transformed yeast cells were pre-grown in synthetic complete medium (ULH^-^) supplemented with 2% glucose and 100 μM BCS at 29°C for overnight, then FHV RNA replication was induced by transferring the yeast to synthetic complete medium (ULH^-^) supplemented with 2% galactose and 50 μM CuSO_4_ at 23°C for 48 h. Yeast total RNA and total protein were isolated and analyzed by northern blotting and western blotting with anti-Flag antibody, respectively [3,9].

To test the effect of yeast Vps5 protein in the replication of Nodamura virus (NoV), yeast strains BY4741 and vps5Δ were transformed with plasmids pESC-His-Cup1-NoV-RNA1/frameshift/TRSV-RZ, pESC-Leu-Cup1-NoVproteinA-HA-Flag and pESC-Ura. The transformed yeast cells were pre-grown in synthetic complete medium (ULH^-^) supplemented with 2% glucose and 100 μM BCS at 29°C for overnight, then NoV RNA replication was induced by transferring the yeast to synthetic complete medium (ULH^-^) supplemented with 2% galactose and 50 μM CuSO_4_ at 29°C for 48 h. Yeast total RNA and total protein were isolated and analyzed by northern blotting and western blotting with anti-Flag antibody, respectively [3,9].

**Analysis of viral replication in yeast expressing Ago1 and Dcr1**

Dcr1 and Ago1 proteins from *S. castellii* were expressed as an intracellular RNAi probe in yeast as described [7]. Yeast strains BY4741 and vps5Δ were transformed with plasmids pGAD-Cup1-Flag-p92, pGBK-Cup1-Flag-p33-Gal1-DI72 and pESC-Ura-Gal1-HisAgo1-Gal10-HisDcr1 or pESC-Ura empty. Transformed yeast were pre-grown at 29°C overnight in synthetic complete medium (ULH^-^) supplemented with 2% glucose and 100 μM BCS. Then, the yeasts were transferred into synthetic complete medium (ULH^-^) supplemented with 2% galactose and 100 μM BCS at 23°C for 24 h, after that, the yeasts were washed and incubated with synthetic complete medium (ULH^-^) supplemented with 2% galactose and 50 μM CuSO_4_, followed by culturing yeast cells at 23°C for 16 h. Total RNA and total protein were isolated and analyzed as described previously [7].

To dissect the role of PI(3)P phosphoinositide in the formation of viral replicase complex, PI3K inhibitors Wortmannin (Alfa Aesar, Cat#AAJ63983) and AS604850 (Selleck Chemicals, Cat#S2681) were used to inhibit cellular PI(3)P biogenesis [3]. Dcr1 was expressed in yeast as an intracellular probe to measure if PI(3)P contributes to the protection of the viral RNA [7]. DCR1 could produce vsiRNAs if the VRCs do not provide satisfactory protection to the dsRNA replication intermediate. Wild-type yeast was transformed with plasmids pGAD-Cup1-Flag-p92, pGBK-Cup1-Flag-p33-Gal1-DI72 and pESC-Ura-Gal10-HisDcr1. Then, the transformed yeasts were pre-grown at 29°C for overnight in synthetic complete medium (ULH^-^) supplemented with 2% glucose and 100 μM BCS. Then, the yeasts were transferred into synthetic complete medium (ULH^-^) supplemented with 2% galactose and 100 μM BCS at 23°C for 24 h, after that, the yeasts were washed and incubated with synthetic complete medium (ULH^-^) supplemented with 2% galactose and 50 μM CuSO_4_, in combination with 15 μM Wortmannin or 15 μM AS604850, followed by culturing yeast cells at 23°C for 10 h. Viral repRNA and protein accumulation were determined as above. For viral small interfering RNAs (vsiRNAs) detection, the small RNAs were isolated using PEG8000 solution as previously described [7,10].

**References**

1. Kovalev N, Nagy PD (2013) Cyclophilin a binds to the viral RNA and replication proteins, resulting in inhibition of tombusviral replicase assembly. J Virol 87: 13330-13342.

2. Rajendran KS, Nagy PD (2003) Characterization of the RNA-binding domains in the replicase proteins of tomato bushy stunt virus. J Virol 77: 9244-9258.

3. Feng Z, Xu K, Kovalev N, Nagy PD (2019) Recruitment of Vps34 PI3K and enrichment of PI3P phosphoinositide in the viral replication compartment is crucial for replication of a positive-strand RNA virus. PLoS Pathog 15: e1007530.

4. Kovalev N, Pogany J, Nagy PD (2020) Reconstitution of an RNA virus replicase in artificial giant unilamellar vesicles supports full replication and provides protection for the dsRNA replication intermediate. J Virol.

5. Pogany J, Stork J, Li Z, Nagy PD (2008) In vitro assembly of the Tomato bushy stunt virus replicase requires the host Heat shock protein 70. Proc Natl Acad Sci U S A 105: 19956-19961.

6. Pogany J, Nagy PD (2008) Authentic replication and recombination of Tomato bushy stunt virus RNA in a cell-free extract from yeast. J Virol 82: 5967-5980.

7. Kovalev N, Inaba JI, Li Z, Nagy PD (2017) The role of co-opted ESCRT proteins and lipid factors in protection of tombusviral double-stranded RNA replication intermediate against reconstituted RNAi in yeast. PLoS Pathog 13: e1006520.

8. Kovalev N, Pogany J, Nagy PD (2012) A Co-Opted DEAD-Box RNA Helicase Enhances Tombusvirus Plus-Strand Synthesis. PLoS Pathog 8: e1002537.

9. Panavas T, Nagy PD (2003) Yeast as a model host to study replication and recombination of defective interfering RNA of Tomato bushy stunt virus. Virology 314: 315-325.

10. Pall GS, Hamilton AJ (2008) Improved northern blot method for enhanced detection of small RNA. Nat Protoc 3: 1077-1084.
