# Supplementary material for: Key interplay between the co-opted sorting nexin-BAR proteins and PI3P phosphoinositide in the formation of the tombusvirus replicase
Source: PLoS Pathog. 2020 Dec 28;16(12):e1009120. doi: 10.1371/journal.ppat.1009120 (PMC7833164; doi:10.1371/journal.ppat.1009120)
Supplement: S1 Table — (DOCX) [file ppat.1009120.s002.docx]

**S1 Table: Yeast PI3P binding proteins screened in this study**

| **#** | **Gene name** | **Cellular function** | **repRNA accumulation** |
| --- | --- | --- | --- |
| 1 | Snx4/Atg24 | Sorting nexin; involved in retrieval of late-Golgi SNAREs from post-Golgi endosomes to the trans-Golgi network and in cytoplasm to vacuole transport. | 110 ±22 |
| 2 | Rgd1 | Subunit beta1 of the nascent polypeptide-associated complex (NAC); involved in protein targeting, associated with cytoplasmic ribosomes. | 87±5 |
| 3 | Egd2 | Alpha subunit of the nascent polypeptide-associated complex (NAC); involved in protein sorting and translocation; associated with cytoplasmic ribosomes | 210±9 |
| 4 | Pib1 | RING-type ubiquitin ligase of the endosomal and vacuolar membranes. | 105±6 |
| 5 | Bem1 | Involved in establishing cell polarity and morphogenesis; functions as a scaffold protein for complexes that include Cdc24p, Ste5p, Ste20p, and Rsr1p. | 108±3 |
| 6 | Bem3 | Rho GTPase activating protein (RhoGAP); involved in control of the cytoskeleton organization; targets the essential Rho-GTPase Cdc42p, which controls establishment and maintenance of cell polarity, including bud-site assembly. | 78±6 |
| 7 | Nvj3 | Protein with a potential role in tethering ER and vacuoles; localizes to nucleus-vacuole junctions in an Mdm1p-dependent manner. | 90±7 |
| 8 | Ent3 | Involved in clathrin recruitment and traffic between the Golgi and endosomes. | 110±28 |
| 9 | Snx3 | Sorting nexin for late-Golgi enzymes; required to maintain late-Golgi resident enzymes in their proper location by recycling molecules from the prevacuolar compartment. | 81±4 |
| 10 | Snx41 | Sorting nexin; involved in the retrieval of late-Golgi SNAREs from the post-Golgi endosome to the trans-Golgi network; interacts with Snx4p. | 115±12 |
| 11 | Mvp1 | Protein required for sorting proteins to the vacuole; Mvp1p and Vps1p act in concert to promote membrane traffic to the vacuole; participates in transcription initiation and/or early elongation of specific genes. | 99±5 |
| 12 | Vps36 | Component of the ESCRT-II complex; contains the GLUE (GRAM Like Ubiquitin binding in EAP45) domain which is involved in interactions with ESCRT-I and ubiquitin-dependent sorting of proteins into the endosome. | 20±8 |
| 13 | Vps5 | Nexin-1 homolog; required for localizing membrane proteins from a prevacuolar/late endosomal compartment back to late Golgi; structural component of retromer membrane coat complex; forms a retromer subcomplex with Vps17p; required for recruiting the retromer complex to the endosome membranes. | 18±12 |
| 14 | Vps17 | Subunit of the membrane-associated retromer complex; essential for endosome-to-Golgi retrograde protein transport; peripheral membrane protein that assembles onto the membrane with Vps5p to promote vesicle formation; required for recruiting the retromer complex to the endosome membranes. | 33±10 |

1. Yeast deletion strains were used in this study.

2. The annotation of gene function is edited and provided from *Saccharomyces* Genome Database (https://www.yeastgenome.org/).
