# Supplementary material for: Key interplay between the co-opted sorting nexin-BAR proteins and PI3P phosphoinositide in the formation of the tombusvirus replicase
Source: PLoS Pathog. 2020 Dec 28;16(12):e1009120. doi: 10.1371/journal.ppat.1009120 (PMC7833164; doi:10.1371/journal.ppat.1009120)
Supplement: S2 Table — (PDF) [file ppat.1009120.s003.pdf]

**S2 Table. Amino acid sequence comparison of the BAR-domains of SNX-BAR proteins**

| | | |
|---|---|---|
| AtSnx1 BAR | -fRDVQSKVSDAVLGK-EKPVEETTADYEKLKHYIFELENHLTEAQKHAYRLVKRHRELGQSLLDFGKAVKLLGACEG-- | 76 |
| NbSnx1 BAR | ----VQSKMSDVVLGK-EKPVEESTPEYEKMKSYIFELEDHLAEAQKHAYRLVKRHRELGESLSEFGKAVKLLGTCED-- | 73 |
| AtSnx2a BAR | lfKELRQSVSNDWGGS-KPPVVEEDKEFLEKKEKMHDLEQQIINASQQAESLVKAQQDMGETMGELGLAFIKLTKFENEE | 79 |
| Vps5 BAR | --DAESHKGFMSISFSsLPKYNEADEFFIEKKQKIDELEDNLKKLSKSLEMVDTSRNTLAASTEEFSSMVETLASLNVS- | 77 |
| NbSnx2b BAR | -----------------------------------------------QAEALVKAQQDIGETMGQMGLAFVKLTKFETEQ | 33 |
| AtSnx2b BAR | ---------------------------------------------------LVKAQQDMGETMGELGLAFIKLTKFENEE | 29 |
| | | |
| AtSnx1 BAR | ------EPTGKAFSDLGTKSELLSIKLQKEAQQVLMNFEEPLKDYVRYVQSIKATIAERGTAFKQHCELSETTKLKEINL | 150 |
| NbSnx1 BAR | ------DALGKAFSELGAKSEIISIKLQKEAHHLLMNFEEPLKDYVRAVQSIKATITERANAFKQQCELAETIKFKEIDL | 147 |
| AtSnx2a BAR | AVCNPQRTRANDMKNLATAAVKASRFYRELNSQTVKHL-DTLHEYLGMMMAVQGAFADRSSALLTVQTLLSELPSLQTRV | 158 |
| Vps5 BAR | ------EPNSELLNNFADVHKSIKSSLERSSLQETLTMGVMLDDYIRSLASVKAIFNQRSKLGYFLVVIENDMNKKHSQL | 151 |
| NbSnx2b BAR | AVYDSQRTRAADMKNVATAAVKASRLYRELNAQTVKHL-DKLHEYLGVMLAVNNAFSDRSSALLTVQTLLSELSSLNSRI | 112 |
| AtSnx2b BAR | AVFNSQRARANDMKNLATSAVKASRFYRELNSQTVKHL-DTLHDYLGLMMAVQGAFADRSSALLTVQTLLSELSSLEARA | 108 |
| | | |
| AtSnx1 BAR | DKLMLT----------RSDKVGEAEIEYREIKAESEEATRRFERIVKRMEDEIVRFQEQKTEEMGVAFHQFAKGQARLAN | 220 |
| NbSnx1 BAR | NKYRLT----------RSDKLSEAEREYEMLKAEGEEASRRFDRIVRLMNEEIVRFQEQKTLDMGLAFHEFAKGQARLAN | 217 |
| AtSnx2a BAR | EKLEAASSKVFGGDKSRIRKIEELKETIKVTEDAKNVAIKGYERIKENNRSEVERLDRERRADFMNMMKGFVVNQVGYAE | 238 |
| Vps5 BAR | GKLGQNI---------HSEKFREMRKEFQTLERRYNLTKKQWQAVGDKIKDEFQGFSTDKIREFRNGMEISLEAAIESQK | 222 |
| NbSnx2b BAR | EKLEAASSKIFGGDRSRIRKIEELKETLRVTEDAKSTAVREYERIKENNKNELERFEKERHDDFLGMLRGFIVNQAGYAE | 192 |
| AtSnx2b BAR | EKLEVASSKVFGGDKSRIKKIEELKETIKVTEDSKNVAIREYEQIKENNWSEVERLDRERRADFLNMMKGFVANQVGYAE | 188 |
| | | |
| AtSnx1 BAR | SVADAWRSLLPKLEA--- | 235 |
| NbSnx1 BAR | GIADGWRSLLPKLEAfss | 235 |
| AtSnx2a BAR | KMGNVWAKVAEETSQ--- | 253 |
| Vps5 BAR | ECIELWETFYQT------ | 234 |
| NbSnx2b BAR | KMANVWETVAEETSG--- | 207 |
| AtSnx2b BAR | KIANVWTKVAEETRQ--- | 203 |
