# Supplementary material for: Key interplay between the co-opted sorting nexin-BAR proteins and PI3P phosphoinositide in the formation of the tombusvirus replicase
Source: PLoS Pathog. 2020 Dec 28;16(12):e1009120. doi: 10.1371/journal.ppat.1009120 (PMC7833164; doi:10.1371/journal.ppat.1009120)
Supplement: S3 Table — (PDF) [file ppat.1009120.s004.pdf]

S3 Table. **Amino acid sequence comparison of the PX-domains of SNX-BAR proteins**

Vps5 PX	kaVAFKVEVKDPVK-------VGELTSIHVEYTVISESSLLELK---YAQVSRRYRDFRWLYRQLQNNHWGKVIPPPPEK	70
AtSnx1 PX	-hPYLSVSVTDPVK-------LGNGVQAYISYRVITKTNLPEYQG-PEKIVIRRYSDFVWLRDRLFEKYKGIFIPPLPEK	71
NbSnx1 PX	--PFLSVSVTDPAK-------MGNGVQAYISYKVITKTNLPEYQG-HEKIVIRRYSDFVWLRDRLFEKYKGIFIPPLPEK	70
AtSnx2b PX	---------SNPQKEQEATNSMIPGGSTYITYQITTRTNLSDYGG-SEFSVRRRFRDIVTLADRLAESYRGFCIPPRPDK	70
AtSnx2a PX	--DYIKITVSNPQKEQEISNSIV-GGNTYITYQITTRTNLPDFGGpSEFSVRRRFRDVVTLADRLAETYRGFCIPPRPDK	77
NbSnx2b PX	----------DPLKEQELANSLVPGGSNYVTYLITTRTNLPEFDG-TEFSVRRRFRDVVTLSDRLAESYRGFFIPLRPDK	69

Vps5 PX	QSVGS---FKENFIENRRFQMESMLKKICQDPVLQKDKDFLLFLTSD----	114
AtSnx1 PX	SAVEK-FRFSAEFIEMRRAALDIFVNRIALHPELQQSEDLRTFLQAD----	117
NbSnx1 PX	STVEK-FRFSAEFIEMRRQALDVFVNRIASHHELRQSDDLRTFLQADeqtm	120
AtSnx2b PX	SIVESQVMQKQEFVEQRRVALEKYLRRLVAHPVIRNSDELKVFLQAQ----	117
AtSnx2a PX	SVVESQVMQKQEFVEQRRVALEKYLRRLSAHPVIRNSDELKVFLQVQ----	124
NbSnx2b PX	SVVESQVMQKQEFLEQRRAALEKYLRRLAAHPLIRRSEELRMFLEAN----	116
