# Supplementary material for: Key interplay between the co-opted sorting nexin-BAR proteins and PI3P phosphoinositide in the formation of the tombusvirus replicase
Source: PLoS Pathog. 2020 Dec 28;16(12):e1009120. doi: 10.1371/journal.ppat.1009120 (PMC7833164; doi:10.1371/journal.ppat.1009120)
Supplement: S4 Table — (DOCX) [file ppat.1009120.s005.docx]

**S4 Table. Nucleotide sequence comparison of AtSnx2a and AtSnx2b**

Percent Identity: 79.77

AtSnx2a atgatgggctcagagaatgccgatggattcgaagaaactaatctcaatgcgcaaagggat 60

AtSnx2b atgatgggctcagagaatgacgaagagt---------cccatctccactcatccaaagaa 51

******************* *** * * * ***** * * * **

AtSnx2a gatatggagaatctcgacctaggagtagatggtggtgaccatcctctgaagataagcgat 120

AtSnx2b gagatggagaaactctt------tctccgcgaagatggcgatcccttgaccaaaagcaat 105

** ******** *** * * * ** * **** *** * **** **

AtSnx2a gtcaacggtgatacatcgaactctggttaccgtagcgccatgtcaacactctccaacgtc 180

AtSnx2b gtcaacggcgataaatccaactctaattatcgcagcgctatgtcaactctcttcgactcg 165

******** **** *** ****** *** ** ***** ******** **** * **

AtSnx2a cgtgacccgctctcgccgccaccgaccgttatgattccagctgattctgatccactcctt 240

AtSnx2b cgtcacccgtc------------gattgtcgtaactccagctgattccgatccacttttt 213

*** ***** ** ** * * ************ ******** **

AtSnx2a gcgccatcctcctacgaagatttccgcagcagc------ttcagctctaagccgatcagc 294

AtSnx2b gcgccaccgtcttactacagcgaatctcggagtcctcgttctaaaccaaacggcggcgac 273

****** * ** *** * * ** * * * ** * *

AtSnx2a agcgataactcctacatcgagcctccgtcgtatgcagatgttatcttcagtccctttgat 354

AtSnx2b agagtcagctcttacctcgagcctccgtcttacgcggatgttatcttcagccctttcgat 333

** * * *** *** ************* ** ** ************** ** ** ***

AtSnx2a gagaactcagatagcgagattaacggtaccgaggataatagccttcatagtcaattctca 414

AtSnx2b gatatctccgagatcaatgg------------ctcagaagatggtcatagtcagtcgtca 381

** * *** ** * * * * ********* * ***

AtSnx2a gattcactatcgagatctccttcttcttcaagctctgattacatcaagatcactgtatct 474

AtSnx2b gattcattatctagatctccgtcttctttgagctctgattacatcaagatcactgtttct 441

****** **** ******** ******* ************************** ***

AtSnx2a aaccctcagaaagagcaggagatttcaaattccattgtt---ggaggaaacacttacatc 531

AtSnx2b aaccctcagaaagagcaagaggccacaaattcaatgattcctggaggaagtacttacatc 501

***************** *** ******* ** ** ******* *********

AtSnx2a acctaccaaattacgaccagaacgaatctccctgactttggcggcccatcggagtttagc 591

AtSnx2b acttaccagattacaactagaacgaatctctctgactac---ggtggatcggagtttagc 558

** ***** ***** ** ************ ****** ** *************

AtSnx2a gtgcgaagaagattcagagatgttgtcacattggctgatcgattggctgagacgtataga 651

AtSnx2b gtgaggagaagattcagagacattgttacattggctgatagattagctgagtcttataga 618

*** * ************** **** ************ **** ****** * ******

AtSnx2a gggttctgcataccaccacggccagataagagcgtcgtagagagccaagtaatgcagaaa 711

AtSnx2b ggtttctgcattccaccgaggccagataagagtatagttgagagtcaagtgatgcaaaag 678

** ******** ***** ************* * ** ***** ***** ***** **

AtSnx2a caagagttcgtggagcagagaagagttgcattggagaaatacttgcgcaggctaagtgca 771

AtSnx2b caagagtttgtggagcagagaagagttgcattggagaagtatttgcgtaggcttgttgca 738

******** ***************************** ** ***** ***** ****

AtSnx2a caccctgttattaggaacagtgacgagttgaaagtgtttcttcaggtgcaagggaagttg 831

AtSnx2b catcctgtgatcaggaacagtgatgaattgaaggtttttcttcaagcgcaagggaagtta 798

** ***** ** *********** ** ***** ** ******** * ************

AtSnx2a ccactaccaatgagtactgatgtggcctctcggatgttggatggggcggtgaagctccca 891

AtSnx2b ccgcttgctacgagcacagatgtggcttctaggatgttggatggtgctgtgaagctgccg 858

** ** * * *** ** ******** *** ************* ** ******** **

AtSnx2a aaacagttgttcggtgaaggcggagcctctgcagtgccggtgactgaggtgggtcagcct 951

AtSnx2b aaacagttgtttggtgaaggcggtggagctt------cttcggttgaggtggttcagccg 912

*********** *********** * ** * * ******** ******

AtSnx2a gcaagaggaggtagggatctgctgagattattcaaggagctgaggcagtcagtttctaat 1011

AtSnx2b ggtagaggaggtagagattttctgagaatgtttaaagaacttagacagtcagtttctaat 972

* *********** *** * ****** * ** ** ** ** ** ***************

AtSnx2a gactggggtggatcaaaacctcccgttgttgaagaagataaagagttcttggaaaagaag 1071

AtSnx2b gactggggtggatcaaaaccacctgttgtggaagaagataaagagtttttggagaagaag 1032

******************** ** ***** ***************** ***** ******

AtSnx2a gagaagatgcatgatcttgagcagcaaattatcaatgcctcacagcaggcggaatccctt 1131

AtSnx2b gagaaaatgtatgatcttgagcaacagatcattaatgcttcacagcaggctgaatccctc 1092

***** *** ************* ** ** ** ***** *********** ********

AtSnx2a gtgaaagcacagcaagacatgggtgaaaccatgggtgaactgggattagcattcattaag 1191

AtSnx2b gtgaaggcacagcaagacatgggggagactatgggggaattgggattagcattcattaaa 1152

***** ***************** ** ** ***** *** *******************

AtSnx2a ctgactaagttcgagaacgaggaagctgtctgcaacccccaaagaactcgtgctaatgat 1251

AtSnx2b ctgacgaaattcgagaacgaagaagctgtcttcaattctcaaagagctcgtgccaatgat 1212

***** ** *********** ********** *** * ****** ******* ******

AtSnx2a atgaagaatttagccactgctgctgtaaaagcaagcagattttatagggagttgaattcc 1311

AtSnx2b atgaagaatttagccacttcagctgtgaaagcaagcagattttacagagagttgaattcc 1272

****************** * ***** ***************** ** ************

AtSnx2a caaactgtcaaacacttggacacactccatgagtaccttggcatgatgatggctgtccaa 1371

AtSnx2b cagacggtcaagcatttggacacactccacgattaccttggcctaatgatggcagtccag 1332

** ** ***** ** ************** ** ********* * ******** *****

AtSnx2a ggcgcatttgcagatagatctagtgctttactgacagttcagacgcttctatcagagctt 1431

AtSnx2b ggcgcctttgcagatagatctagtgctttattgacagtgcagacgcttctatctgaactt 1392

***** ************************ ******* ************** ** ***

AtSnx2a ccttctctgcaaactagagttgagaagctagaggctgcatcatcgaaggtatttggtggt 1491

AtSnx2b tcttcactggaagcaagagcagaaaagctagaagttgcatcatcaaaggtctttggcggt 1452

**** *** ** * **** ** ******** * ********* ***** ***** ***

AtSnx2a gacaaatcaaggatccgaaaaatagaagagttaaaagaaacaatcaaggtcactgaggat 1551

AtSnx2b gacaaatcaaggattaagaagatagaagagttaaaagaaaccatcaaggtcactgaagac 1512

************** ** ******************** ************** **

AtSnx2a gcaaaaaatgttgccatcaaagggtatgagcgaatcaaggaaaacaaccgatctgaggtt 1611

AtSnx2b tctaaaaatgttgccatcagggagtacgagcagatcaaggaaaataactggagtgaggtt 1572

* **************** * *** **** *********** *** * *******

AtSnx2a gagaggttggacagagaaaggcgtgcagacttcatgaacatgatgaagggttttgttgtt 1671

AtSnx2b gaaaggctggatagggaaaggcgtgcagacttcttgaatatgatgaaagggtttgttgct 1632

** *** **** ** ****************** **** ******** ** ******* *

AtSnx2a aaccaggttggatacgcagagaaaatgggaaacgtctgggcaaaggttgcagaagagacc 1731

AtSnx2b aatcaggttggatatgcagagaagatcgccaatgtgtggacaaaggttgctgaggaaaca 1692

** *********** ******** ** * ** ** *** ********** ** ** **

AtSnx2a agccaatacgatagagagaagcagagcagctaa 1764

AtSnx2b aggcaatacgatagagagagctcttaa------ 1719

** ****************
