# Supplementary material for: Key interplay between the co-opted sorting nexin-BAR proteins and PI3P phosphoinositide in the formation of the tombusvirus replicase
Source: PLoS Pathog. 2020 Dec 28;16(12):e1009120. doi: 10.1371/journal.ppat.1009120 (PMC7833164; doi:10.1371/journal.ppat.1009120)
Supplement: S5 Table — (DOCX) [file ppat.1009120.s006.docx]

**S5 Table. List of primers and plasmid constructs used in this study**

| **Clone** | **Primer** | | **Primer sequence (from 5' to 3')** | **Procedure** |
| --- | --- | --- | --- | --- |
| pEsc-URA-Vps5-3xHA | F | 7064 | ATAAGAATGCGGCCGCATGGACTACGAGGATAATCTAGAAGC | Vps5 fragment was digested with NotI&BglII, inserted into pEsc-URA-3xHA cut with NotI&BglII. |
|  | R | 7065 | GAAGATCTCTGCAGAAGATTGGTTTGGTAGAATGTCTC |  |
| pRS315-Vps5-Flag | F | 6894 | ggaagatctATGGACTACGAGGATAATCTAGAAG | Vps5 fragment was digested with BglII&PstI, inserted into pRS315-cFlag cut with BamHI&PstI. |
|  | R | 7065 | GAAGATCTCTGCAGAAGATTGGTTTGGTAGAATGTCTC |  |
| pRS315-Vps5ΔPX-Flag | F | 6894 | ggaagatctATGGACTACGAGGATAATCTAGAAG | Vps5ΔPX fragment was digested with BglII&PstI, inserted into pRS315-cFlag cut with BamHI&PstI. |
|  | R | 7065 | GAAGATCTCTGCAGAAGATTGGTTTGGTAGAATGTCTC |  |
|  | F | 7015 | CAAAGGTGCCACCAACCGAAGACTTTAGTTCAGAGTCTAAAAAAAGGG |  |
|  | R | 7014 | TTCGGTTGGTGGCACCTTT |  |
| pRS315-Vps5ΔBAR-Flag | F | 6894 | ggaagatctATGGACTACGAGGATAATCTAGAAG | Vps5ΔBAR fragment was digested with BglII&PstI, inserted into pRS315-cFlag cut with BamHI&PstI. |
|  | R | 7690 | CCGCTCGAGctaTGGCTGCAGGTCGATACCCCCATTTTTTAAGAC |  |
| pRS315-Vps5yr-AA-Flag | F | 6894 | ggaagatctATGGACTACGAGGATAATCTAGAAG | Vps5yr-AA fragment was digested with BglII&PstI, inserted into pRS315-cFlag cut with BamHI&PstI. |
|  | R | 7065 | GAAGATCTCTGCAGAAGATTGGTTTGGTAGAATGTCTC |  |
|  | F | 7057 | TGCACAGGTAAGCAGGCGTgcCAGAGATTTCAGGTGGCTATATCGT |  |
|  | R | 7056 | ACGCCTGCTTACCTGTGCA |  |
|  | F | 7059 | AGTTTTAAGGAAAATTTCATTGAAAATgcAAGATTTCAAATGGAAAGCATGTTA |  |
|  | R | 7058 | ATTTTCAATGAAATTTTCCTTAAAACT |  |
| pRS315-Vps17-Flag | F | 7491 | CGGGATCCATGACTTCGGCTGTACCTTATG | Vps17 fragment was digested with BamHI&SmaI, inserted into pRS315-Flag cut with BamHI&SmaI. |
|  | R | 7492 | TCCCCCGGGTTTAGTGGACATGCCCAAAA |  |
| pTRV-NbSnx1/2b | F | 7426 | CCGCTCGAGTTACTTACCTAATTACTACGAGGACGAAC | Overlapping fragment of NbSnx1 and NbSnx2b was digested with BamHI&XhoI, inserted into pTRV2 cut with BamHI&XhoI. |
|  | R | 7427 | CAGTAACTCCTTGTCCTCCTCTACTAC |  |
|  | F | 7428 | GTAGTAGAGGAGGACAAGGAGTTACTGTGGATCAGGGTCATTACAGAG |  |
|  | R | 7375 | CGGGATCCGTCTCCTGGAACCTCGCTCT |  |
| pGD-AtSnx1-GFP  pGD-AtSnx1-Flag | F | 7004 | CGGGATCCATGGAGAGCACGGAGCAGC | AtSnx1fragment was digested with BamHI&SmaI, inserted into pGD-35S-GFP or pGD-35S-Flag cut with BamHI&SmaI. |
|  | R | 7006 | TCCCCCGGGGACAGAATAAGAAGCTTCAAGTTTGG |  |
| pGD-AtSnx2b-GFP  pGD-AtSnx2b-RFP  pGD-AtSnx2b-Flag | F | 6899 | CgcggatccATGATGGGCTCAGAGAATGACGAAG | AtSnx2b fragment was digested with BamHI&PstI, inserted into pGD-35S-GFP, pGD-35S-RFP or pGD-35S-Flag cut with BamHI&PstI. |
|  | R | 6983 | TTGGCTGCAGAGAGCTCTCTCTATCGTATTGCCTTGTTTC |  |
| pGD-p33myc | F | 4000 | ccagagatctatggagaccatcaagagaatg | TBSV p33 fragment was digested with BglII&PstI, inserted into pGD-35S cut with BamHI&PstI. |
|  | R | 7547 | AActgcagCTACAGATCCTCTTCTGAGATGAGTTTTTGTTCTTTGACACCCAGGGACTCC |  |
| pGD-nYFP-AtSnx1 | F | 7004 | CGGGATCCATGGAGAGCACGGAGCAGC | AtSnx1 fragment was digested with BamHI&XhoI, inserted into pGD-35S-nYFP cut with BamHI&SalI. |
|  | R | 7005 | CCGctcgagTTAGACAGAATAAGAAGCTTCAAGTTTGG |  |
| pGD-nYFP-AtSnx2b | F | 6899 | CgcggatccATGATGGGCTCAGAGAATGACGAAG | AtSnx2b fragment was digested with BamHI&SalI, inserted into pGD-35S-nYFP cut with BamHI&SalI. |
|  | R | 6994 | CGACGTCGACTTAAGAGCTCTCTCTATCGTATTGCCTTGT |  |
| pGD-nYFP-AtSnx1rry | F | 7004 | CGGGATCCATGGAGAGCACGGAGCAGC | AtSnx1rry fragment was digested with BamHI&XhoI, inserted into pGD-35S-nYFP cut with BamHI&SalI. |
|  | R | 7005 | CCGctcgagTTAGACAGAATAAGAAGCTTCAAGTTTGG |  |
|  | F | 7557 | aggacccgagaagattgttattGCaGCaGCcagtgatttcgtctggttacg |  |
|  | R | 7556 | AATAACAATCTTCTCGGGTCCT |  |
| pGD-nYFP-AtSnx2bΔPX | F | 6899 | CgcggatccATGATGGGCTCAGAGAATGACGAAG | AtSnx2bΔPX fragment was digested with BamHI&SalI, inserted into pGD-35S-nYFP cut with BamHI&SalI. |
|  | R | 6994 | CGACGTCGACTTAAGAGCTCTCTCTATCGTATTGCCTTGT |  |
|  | F | 7003 | CGTCTTCTTTGAGCTCTGATTACATCGGGAAGTTACCGCTTGCTAC |  |
|  | R | 7002 | GATGTAATCAGAGCTCAAAGAAGACG |  |
| pGD-nYFP-AtSnx2bΔBAR | F | 6899 | cgcggatccATGATGGGCTCAGAGAATGACGAAG | AtSnx2bΔBAR fragment was digested with BamHI&SalI, inserted into pGD-35S-nYFP cut with BamHI&SalI. |
|  | R | 7625 | GACGTCGACttaTTTCTCCTTCTTCTCCAAAAACT |  |
| pGD-nYFP-AtSnx1A | F | 7004 | CGGGATCCATGGAGAGCACGGAGCAGC | AtSnx1A fragment was digested with BamHI&XhoI, inserted into pGD-35S-nYFP cut with BamHI&SalI. |
|  | R | 7005 | CCGctcgagTTAGACAGAATAAGAAGCTTCAAGTTTGG |  |
|  | F | 7875 | cgagaagattgttattagacgatacGCAgatttcgtctggttacggg |  |
|  | R | 7876 | Gtatcgtctaataacaatcttctcg |  |
| pGD-nYFP-AtSnx1E | F | 7004 | CGGGATCCATGGAGAGCACGGAGCAGC | AtSnx1E fragment was digested with BamHI&XhoI, inserted into pGD-35S-nYFP cut with BamHI&SalI. |
|  | R | 7005 | CCGctcgagTTAGACAGAATAAGAAGCTTCAAGTTTGG |  |
|  | F | 7874 | cgagaagattgttattagacgatacGAAgatttcgtctggttacggg |  |
|  | R | 7876 | gtatcgtctaataacaatcttctcg |  |
| pGEX-GST-AtSnx1 | F | 7004 | CGGGATCCATGGAGAGCACGGAGCAGC | AtSnx1 fragment was digested with BamHI&XhoI, inserted into pGEX-GST cut with BamHI&  XhoI. |
|  | R | 7005 | CCGctcgagTTAGACAGAATAAGAAGCTTCAAGTTTGG |  |
| pGEX-GST-AtSnx2b | F | 6899 | cgcggatccATGATGGGCTCAGAGAATGACGAAG | AtSnx2b fragment was digested with BamHI&SalI, inserted into pGEX-GST cut withBamHI&  XhoI. |
|  | R | 6994 | CGACGTCGACTTAAGAGCTCTCTCTATCGTATTGCCTTGT |  |
| pGEX-GST-Vps5 | F | 6894 | ggaagatctATGGACTACGAGGATAATCTAGAAG | Vps5 fragment was digested with BglII&XhoI, inserted into pGEX-GST cut with BamHI&XhoI. |
|  | R | 6895 | CcgctcgagCTAAAGATTGGTTTGGTAGAATGTCTCC |  |
| pMAL-MBP-AtSnx2b | F | 6899 | cgcggatccATGATGGGCTCAGAGAATGACGAAG | AtSnx2b fragment was digested with BamHI&SalI, inserted into pMAL-MBP cut withBamHI&  SalI. |
|  | R | 6994 | CGACGTCGACTTAAGAGCTCTCTCTATCGTATTGCCTTGT |  |
| pGEX-GST-MS2-CP | F | 8008 | CGGGATCCatggcttctaactttactcagtt | MS2-CP fragment was digested with BamHI&XhoI, inserted into pGEX-GST cut withBamHI&  XhoI. |
|  | R | 8009 | CCGCTCGAGttagtagatgccggagtttg |  |
| pGD-MS2-CP-nYFP | F | 8008 | CGGGATCCatggcttctaactttactcagtt | MS2CP fragment was digested with BamHI&PstI, inserted into pGD-35S-cYFP cut with BamHI&PstI. |
|  | R | 7996 | TGGTTCTGCAGGTAGATGCCGGAGTTT |  |
| pESC-TRP-repRNA(+)MS2hp  pESC-TRP-  repRNA(-)MS2hp  pESC-TRP-repRNA | F | 1398 | gcccGAATTCggAAATTCTCCAggATTTCTC | repRNA(+)MS2hp, repRNA(-)MS2hp or repRNA fragment was digested with EcoRI&SacI, inserted into pESC-TRP cut with EcoRI  &SacI. |
|  | R | 1069 | ccggtcgagctcTACCAGGTAATATACCACAACGTGTGT |  |
| RT-PCR | F | 7376 | AGGACGTGCAATCTAAAATGAG | To check NbSnx1 mRNA level |
|  | R | 7377 | AGCCTCTTTCTGCAGCTTAAT |  |
| RT-PCR | F | 7193 | GCCGGACAAGAGTGTGGTT | To check NbSnx2b mRNA level |
|  | R | 7194 | GTCACAGATTGCTTCAACTCCTTA |  |
| BY4741: Vps5-3xHA | F | 7963 | GAGGAACGTGACACATAAAGTTATTGTATACAGATCATTTAATCGATGAATTCGAGCTCG | For Vps5 chromosomal tagging |
|  | R | 7964 | GAATGCATCGAGCTTTGGGAGACATTCTACCAAACCAATCTTCGTACGCTGCAGGTCGAC |  |
| vps5Δvps17Δ | F | 6915 | GGATTTTATAAACTTTCATACATCCTGCAATAACAAGCCATGcgtacgctgcaggtcgac | For Vps5 and Vps17 double deletion |
|  | R | 6916 | GAGGAACGTGACACATAAAGTTATTGTATACAGATCATCTAatcgatgaattcgagctcg |  |
|  | F | 7992 | CGTACTGTACCCTTAGTCAATCCATCTATCCTCTGAACAATGcgtacgctgcaggtcgac |  |
|  | R | 7993 | CTTGTTCAAAGGTATGAATTTTCTACTTTATATACGTATTAatcgatgaattcgagctcg |  |
| pESC-Ura-Gal1-HisAgo1-Gal10-HisDcr1 | [1] | | | |
| pESC-Ura-Gal10-HisDcr1 |  |  |  |  |
| pGD-35S-repRNA(+)MS2hp | [2] | | | |
| pGD-35S-repRNA(-)MS2hp |  |  |  |  |
| pGD-L-p33 / pGD-L-p92 | [3] | | | |
| pGD-35S-repRNA |  |  |  |  |
| pGD-p33-cYFP | [4] | | | |
| pGD-p36-cYFP |  |  |  |  |
| pGD-p92-cYFP |  |  |  |  |

**References**

1. Kovalev N, Inaba JI, Li Z, Nagy PD (2017) The role of co-opted ESCRT proteins and lipid factors in protection of tombusviral double-stranded RNA replication intermediate against reconstituted RNAi in yeast. PLoS Pathog 13: e1006520.

2. Wu CY, Nagy PD (2019) Blocking tombusvirus replication through the antiviral functions of DDX17-like RH30 DEAD-box helicase. PLoS Pathog 15: e1007771.

3. Barajas D, Jiang Y, Nagy PD (2009) A unique role for the host ESCRT proteins in replication of Tomato bushy stunt virus. PLoS Pathog 5: e1000705.

4. Xu K, Nagy PD (2016) Enrichment of Phosphatidylethanolamine in Viral Replication Compartments via Co-opting the Endosomal Rab5 Small GTPase by a Positive-Strand RNA Virus. PLoS Biol 14: e2000128.
