# Supplementary figures and images for: Key interplay between the co-opted sorting nexin-BAR proteins and PI3P phosphoinositide in the formation of the tombusvirus replicase
Source: PLoS Pathog. 2020 Dec 28;16(12):e1009120. doi: 10.1371/journal.ppat.1009120 (PMC7833164; doi:10.1371/journal.ppat.1009120)

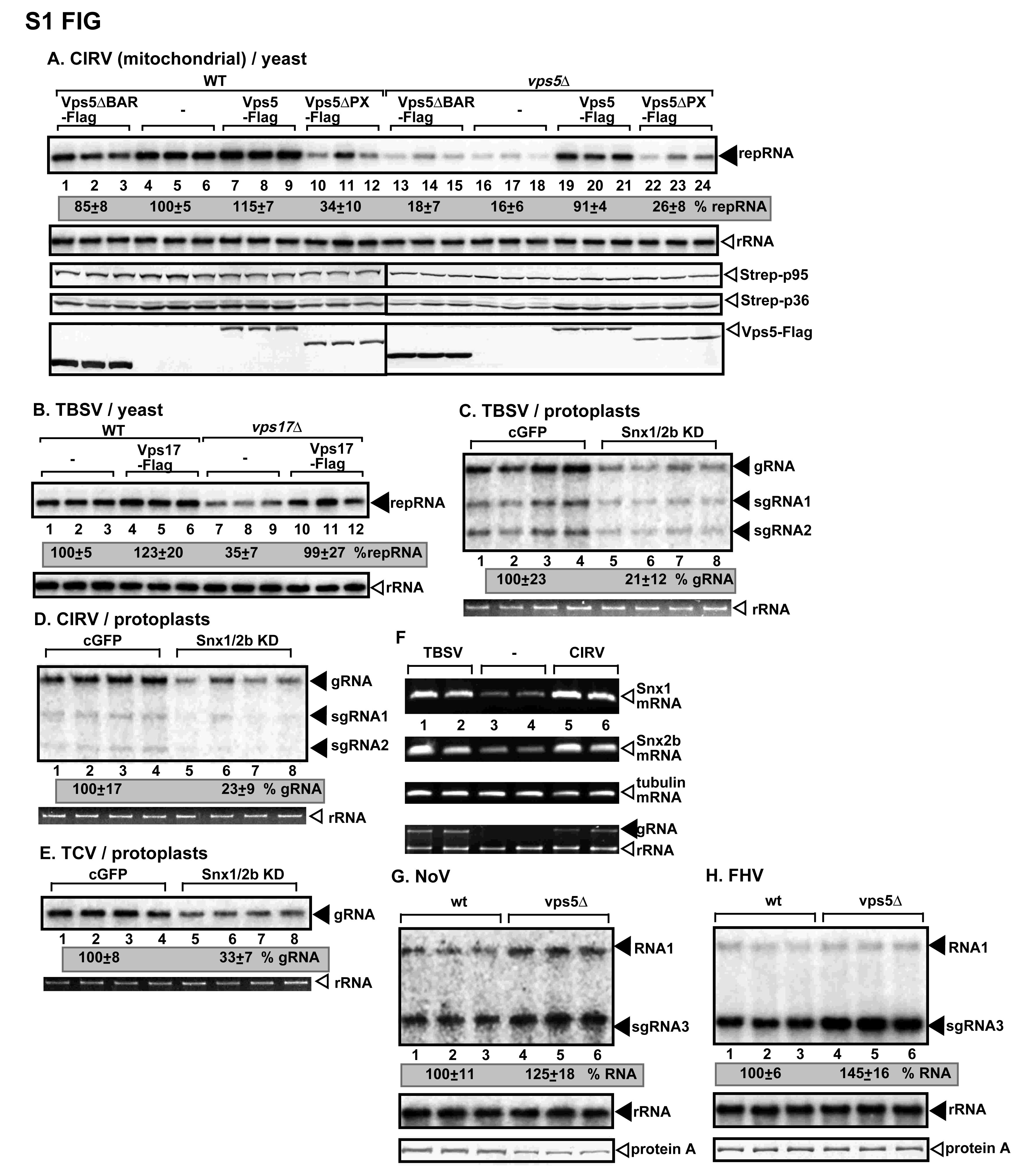

Supplement: S1 Fig — (A) Top image: Northern blot analysis shows decreased CIRV (+)repRNA accumulation in vps5Δ yeast strain. Vps5p and its deletion mutants were expressed from the constitutive TEF1 promoter from a plasmid. The accumulation level of repRNA was normalized based on 18S rRNA levels (second panel). The accumulation of Strep-p36, Strep-p95 and Vps5-Flag is measured by western blotting and anti-Strep or anti-Flag antibodies. See further details in Fig 1A. (B) Top image: Northern blot analysis shows decreased TBSV (+)repRNA accumulation in vps17Δ yeast strain. Vps17p SNX-BAR protein was expressed from the TEF1 promoter from a plasmid. The accumulation level of repRNA was normalized based on 18S rRNA levels (second panel). (C-D-E) VIGS-based knock-down of both SNX1 and SNX2b mRNA levels inhibits the accumulation of TBSV, CIRV and the related TCV RNAs in N. benthamiana protoplasts. Top panel: The accumulation of TBSV, CIRV and TCV gRNA and sgRNAs was measured using Northern blot analysis of total RNA samples obtained from N. benthamiana protoplasts. Second panel: ethidium-bromide stained gels show ribosomal RNA level. Protoplasts were isolated from the upper leaves of SNX1/SNX2b-silenced N. benthamiana on the 12th day, followed by transformation of TBSV, CIRV and TCV RNA, respectively. Twenty-four hours later, total RNA was analyzed by Northern blotting. (F) Up-regulation of Snx1 and Snx2b expression in TBSV and CIRV-infected N. benthamiana leaves. The mRNA levels for the SNX-BAR proteins were estimated by semi-quantitative RT-PCR in total RNA samples obtained from TBSV or CIRV-infected versus mock-infected N. benthamiana leaves. Tubulin mRNA and ribosomal RNA were used as controls (bottom panels). (G-H) The endosomal Vps5p SNX-BAR protein is not required for nodavirus replication in yeast. Top image: Northern blot analysis shows the accumulation level of NoV and FHV (+)RNA1 and the subgenomic RNA3 in vps5Δ yeast strain. The accumulation level of repRNA was normalized based on 1 [file ppat.1009120.s007.tif]

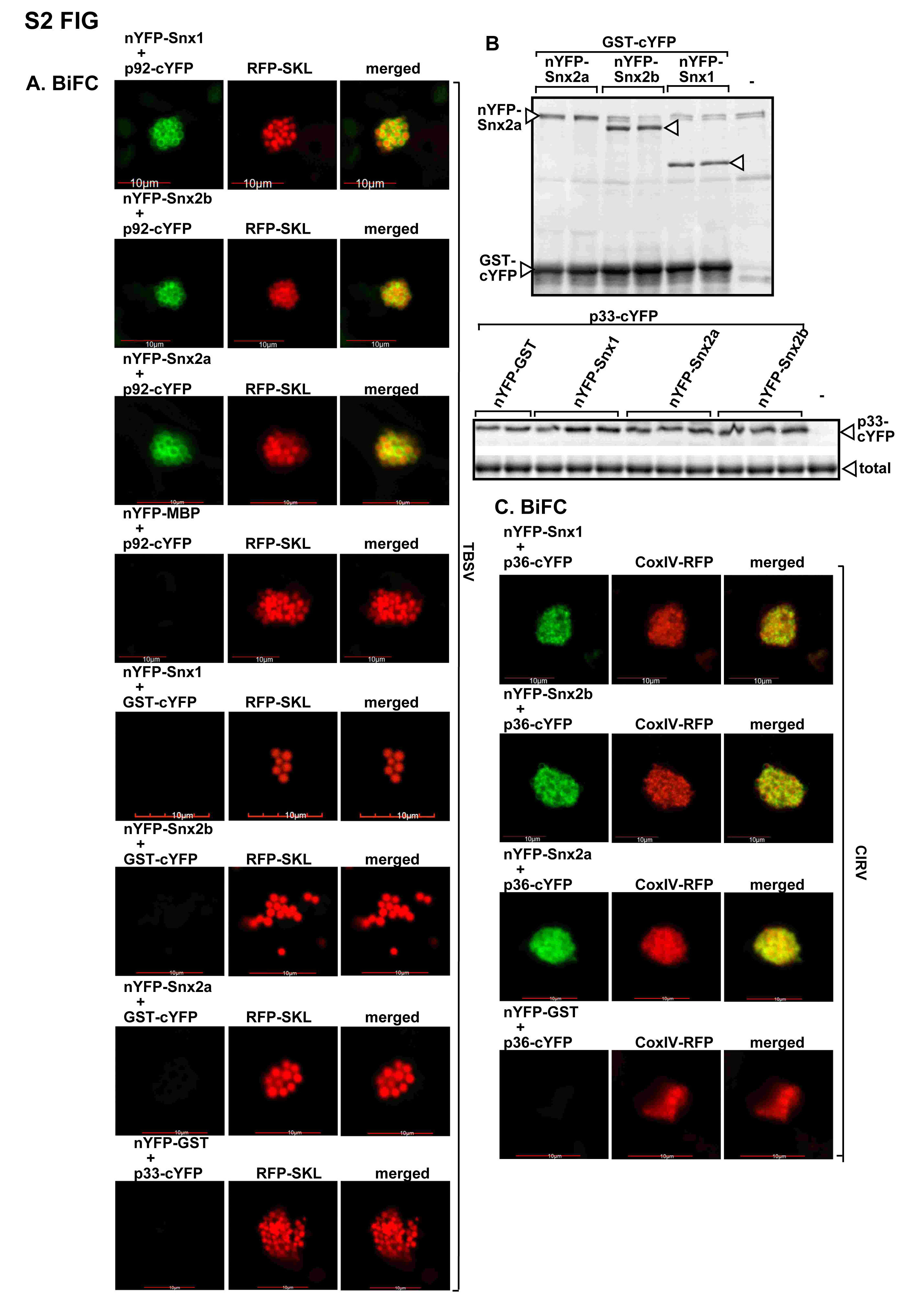

Supplement: S2 Fig — (A) Interaction between TBSV p92pol replication protein and the SNX-BAR proteins was detected by BiFC assay in planta. TBSV p92-cYFP and the nYFP-Snx2b, nYFP-Snx2a or nYFP-Snx1 proteins were co-expressed from the 35S promoter after co-agroinfiltration into N. benthamiana leaves. Bottom three panels: negative control was GST-cYFP and the SNX-BAR proteins analyzed by BiFC assay in planta. Note that the plants were infected with TBSV to induce the viral replication compartments in cells. Co-localization of RFP-SKL peroxisomal luminar marker with the BiFC signals demonstrates that the interaction between p92pol and SNX-BAR proteins occurs in the viral replication compartments. Scale bars represent 10 μm. (B) Western blots show the accumulation levels of the proteins used in the BiFC assays. Top panel for S2A Fig, bottom panels for Fig 3A. (C) Interactions between CIRV p36 replication protein and the SNX-BAR proteins were detected by BiFC assay in planta. CIRV p36-cYFP and nYFP-Snx2b, nYFP-Snx2a or nYFP-Snx1 proteins were co-expressed from the 35S promoter after co-agroinfiltration into N. benthamiana leaves. nYFP-GST was co-expressed with p36-cYFP to serve as the negative control for BiFC assay. Note that the plants were infected with CIRV to induce the viral replication compartments in cells. Scale bars represent 10 μm. (TIF) [file ppat.1009120.s008.tif]

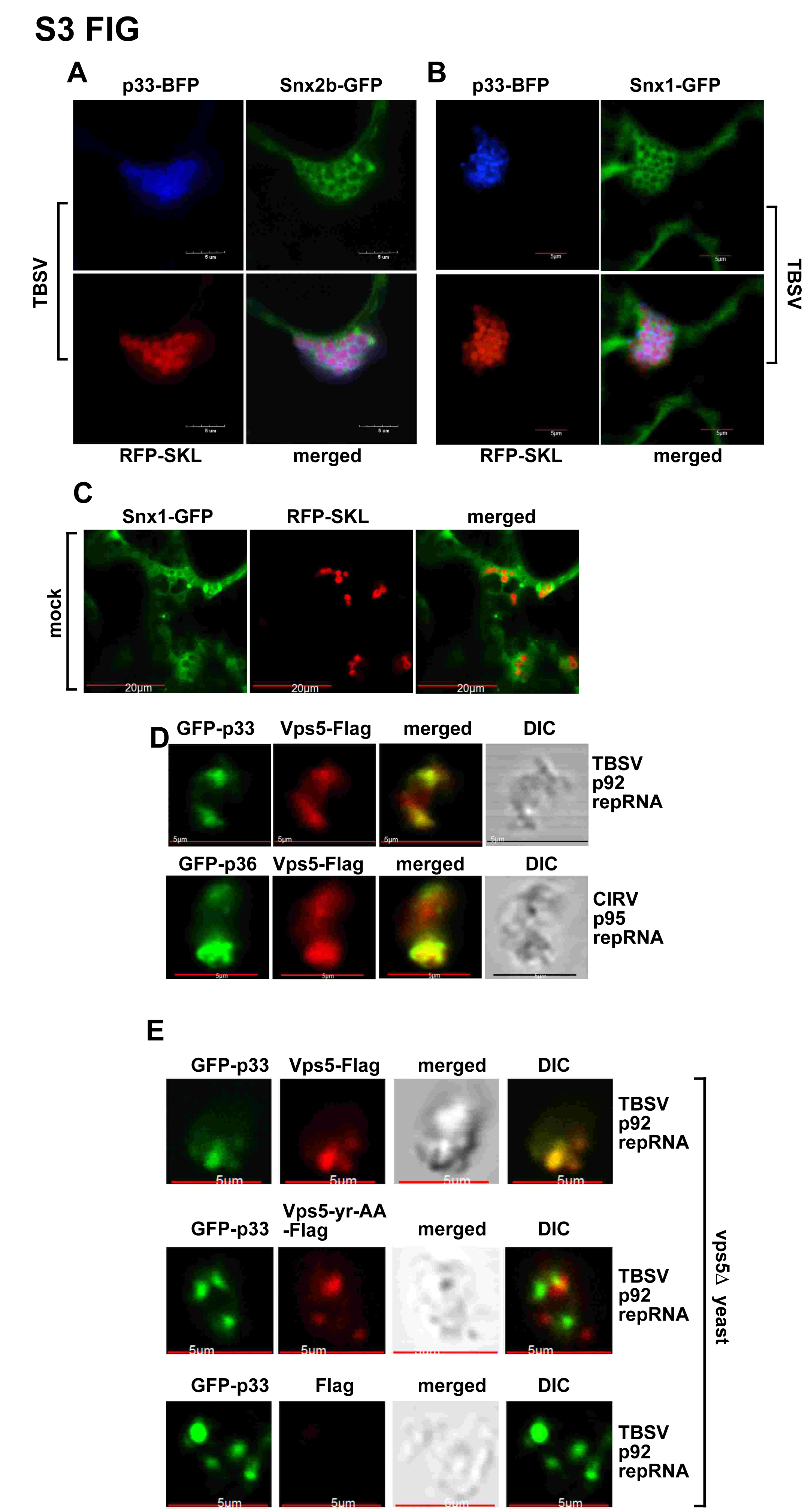

Supplement: S3 Fig — (A-B) Co-localization of TBSV p33-BFP with the GFP-tagged Snx1 and GFP-tagged Snx2b in N. benthamiana cells is detected by confocal laser microscopy. Scale bars represent 5 μm. (C) Localization pattern of Snx1-GFP in the absence of viral components in N. benthamiana cells is detected by confocal laser microscopy. (D) Co-localization of TBSV GFP-p33 and CIRV GFP-p36, respectively, with Vps5-Flag SNX-BAR protein in yeast cells replicating repRNA. Vps5-Flag was detected with anti-Flag antibody. Each experiment was repeated three times. (E) Absent of excessive co-localization of TBSV GFP-p33 with Vps5yr-AA-Flag mutant protein in yeast cells replicating repRNA. (TIF) [file ppat.1009120.s009.tif]

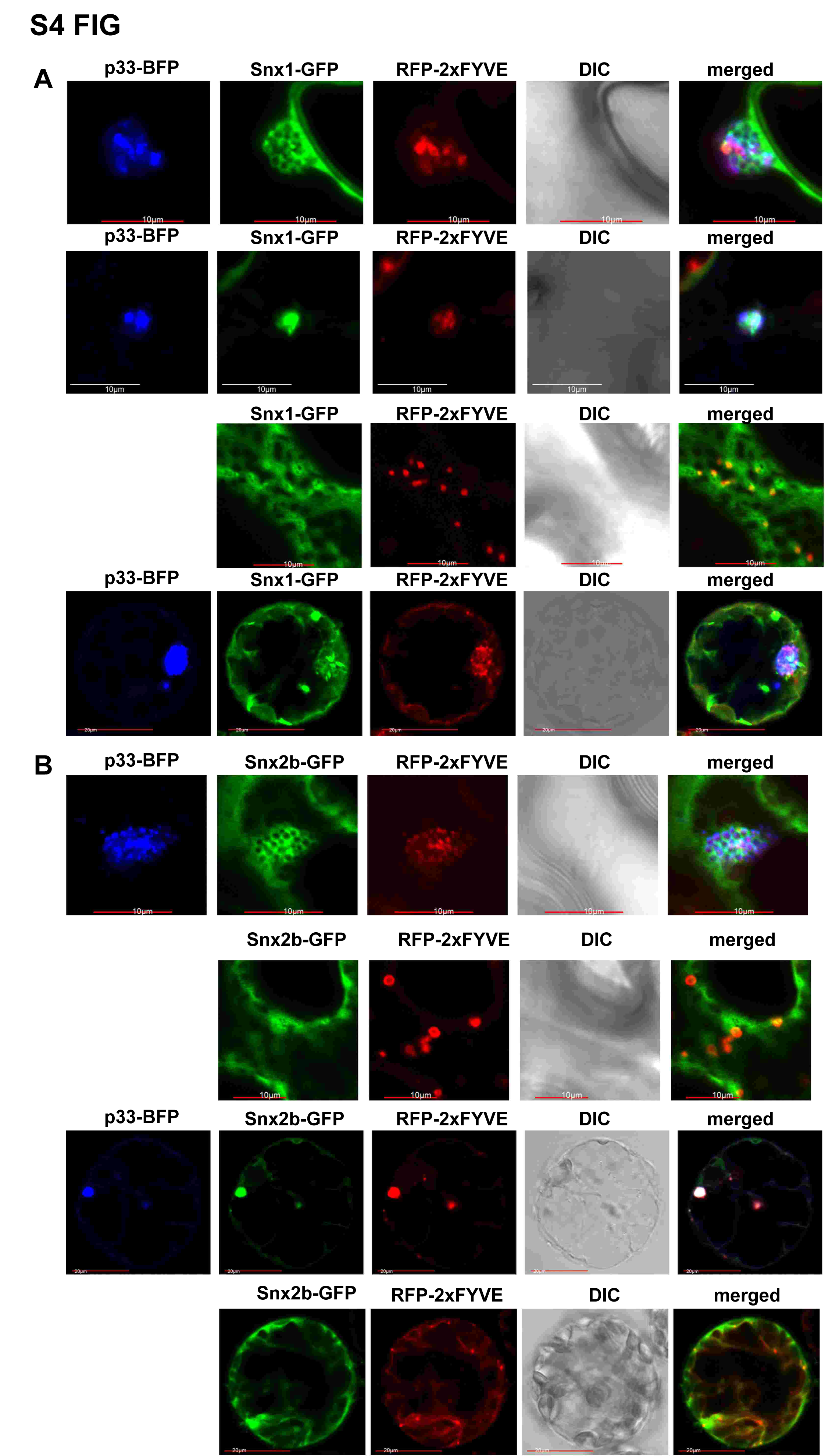

Supplement: S4 Fig — (A) Co-localization of TBSV p33-BFP with the GFP-tagged Snx1 and with RFP-2xFYVE protein in N. benthamiana leaf tissues and protoplasts is detected by confocal laser microscopy. RFP-2xFYVE protein binds PI(3)P selectively. Top two panels: Co-localization of TBSV p33-BFP with the GFP-tagged Snx1 and with RFP-2xFYVE protein in N. benthamiana leaves. Middle panel represents images in the absence of viral components. Scale bars represent 10 μm. Bottom panel shows the co-localization of TBSV p33-BFP with the GFP-tagged Snx1 and with RFP-2xFYVE protein in N. benthamiana protoplasts. Scale bars represent 20 μm. (B) Co-localization of TBSV p33-BFP with the GFP-tagged Snx2b and with RFP-2xFYVE protein in N. benthamiana leaf tissues and protoplasts is detected by confocal laser microscopy. Top panel: Co-localization of TBSV p33-BFP with the GFP-tagged Snx1 and with RFP-2xFYVE protein in N. benthamiana leaves. Second panel represents images in the absence of viral components. Scale bars represent 10 μm. Third panel shows the co-localization of TBSV p33-BFP with the GFP-tagged Snx2b and with RFP-2xFYVE protein in N. benthamiana protoplasts. Bottom panel represents images in the absence of viral components. Scale bars represent 20 μm. (TIF) [file ppat.1009120.s010.tif]

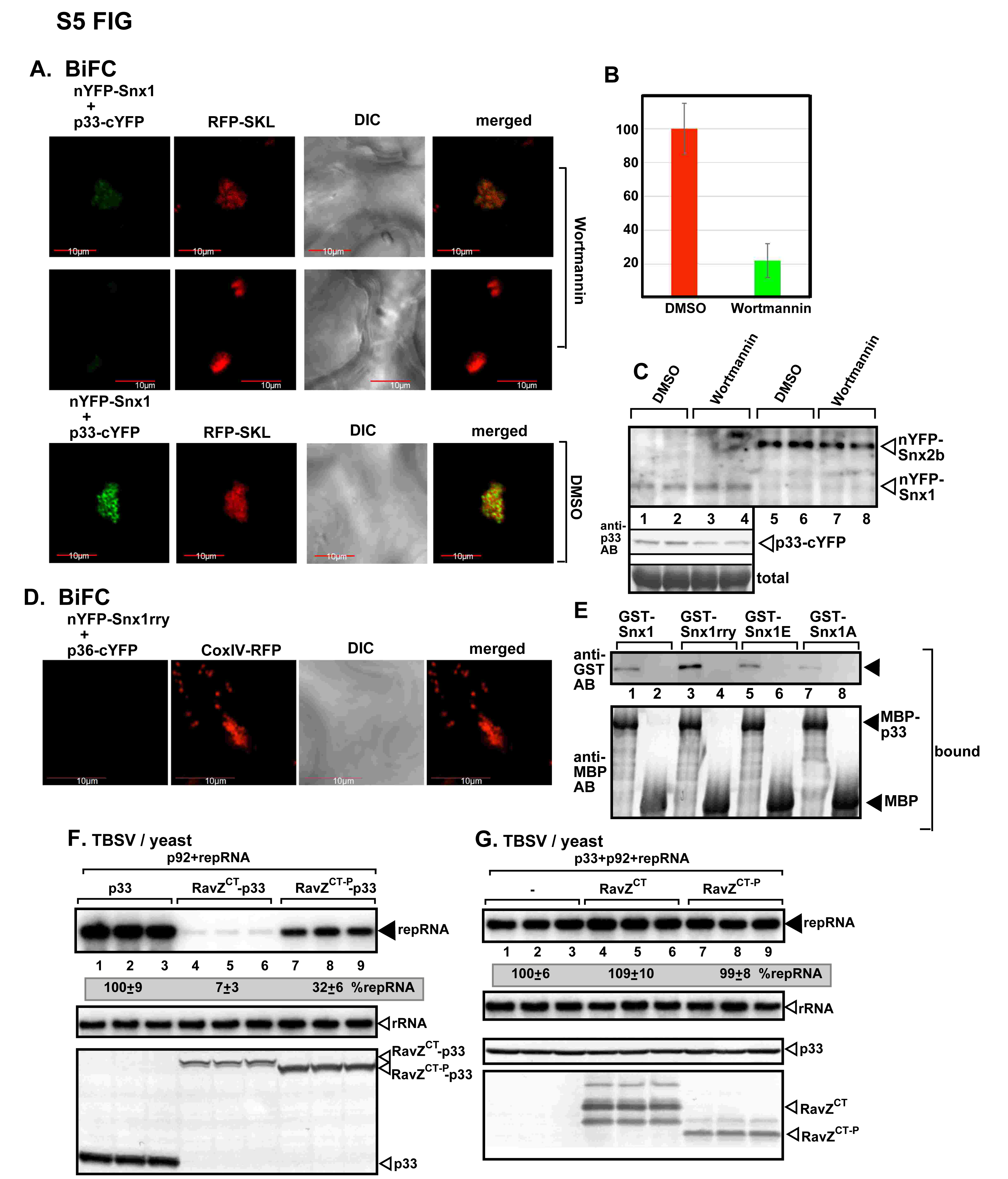

Supplement: S5 Fig — (A-B) BiFC assay shows the reduced level of interaction between p33 replication protein and the Snx1 protein in N. benthamiana treated with Wortmannin, a Vps34 PI3K inhibitor, or with DMSO as a negative control. TBSV p33-cYFP and nYFP-Snx1 proteins were co-expressed from the 35S promoter after co-agroinfiltration into N. benthamiana leaves. RFP-SKL was expressed as a peroxisomal marker to identify the viral replication compartments. Scale bars represent 10 μm. The BiFC signals were quantified via Image J. Each experiment was repeated. (C) Western blot analysis shows the accumulation level of nYFP-Snx1 and nYFP-Snx2b proteins and p33-cYFP in Wortmannin or DMSO-treated N. benthamiana leaves. (D) BiFC assay shows that nYFP-Snx1rry protein was not recruited by CIRV p36-cYFP replication protein into the viral replication compartment in N. benthamiana infected with CIRV. The CIRV replication compartment was decorated with CoxIV-RFP mitochondria marker protein. Scale bars represent 10 μm. (E) Pull-down assay shows direct interaction of TBSV p33 replication protein with the shown Snx1 mutants in vitro. Top panel: Western blot analysis of the captured GST-Snx1 mutants with the MBP-affinity purified p33 (lanes 1, 3, 5 and 7). The negative control was MBP (lanes 2, 4, 6 and 8). Bottom panel: The captured MBP-p33 and MBP were detected with anti-MBP antibody. Note that equal amount of each GST fusion protein was incubated with MBP-p33 or MBP. (F) Expression of RavZCT-p33 fusion protein inhibits TBSV replication in yeast. Reduced repRNA accumulation by expression of RavZCT-p33 fusion protein in comparison with p33 replication protein in wt yeast replicating TBSV repRNA. RavZCT-p33 fusion protein lacking the PI(3)P-binding domain (i.e., RavZCT-P) loses the strong inhibitory effect on TBSV replication. Top panel shows the northern blot analysis of (+)repRNA accumulation. Middle panel: the 18S rRNA level. Accumulation of p33 and the fusion proteins was shown in bottom panel. (G) Co- [file ppat.1009120.s011.tif]

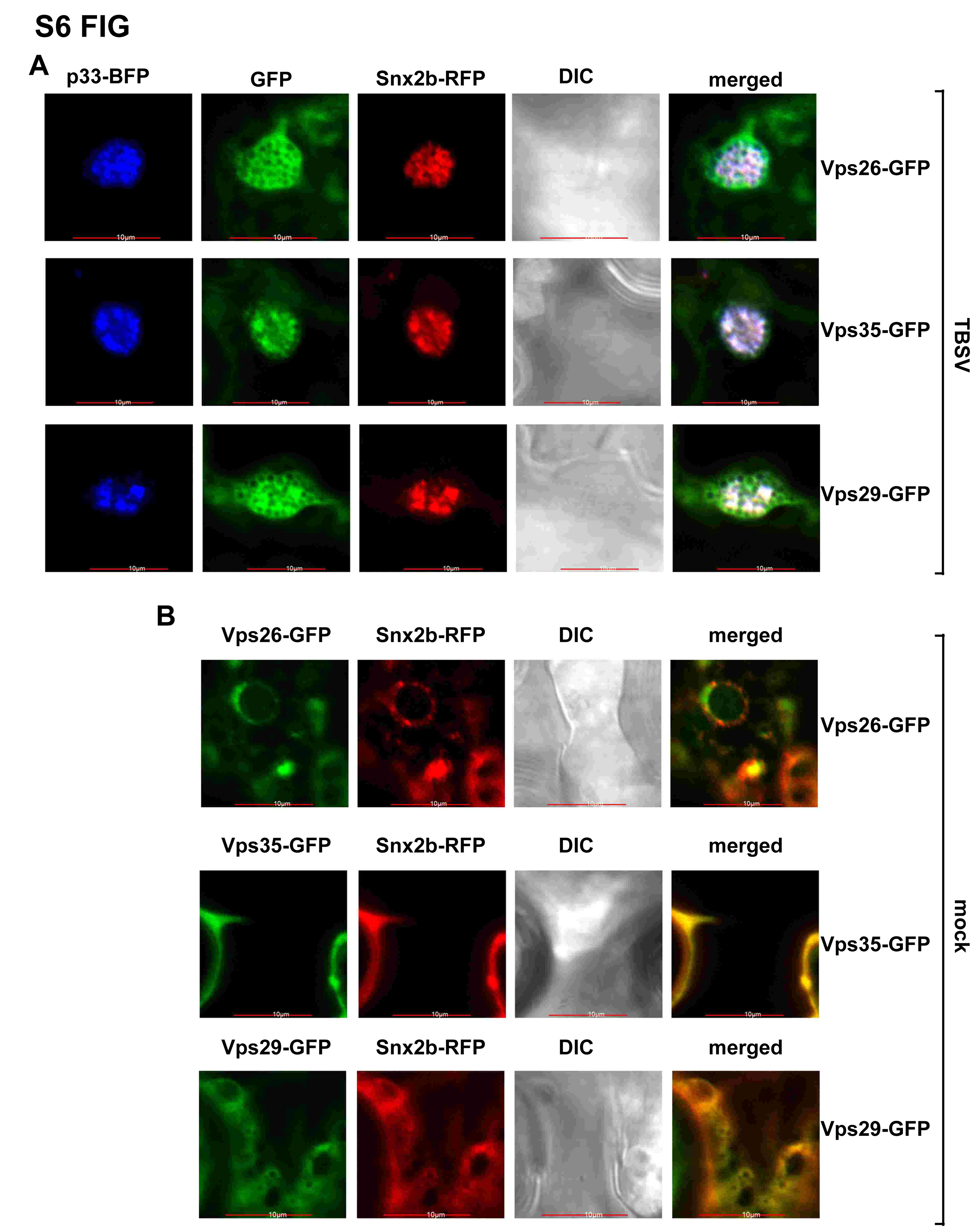

Supplement: S6 Fig — (A) Co-localization of TBSV p33-BFP with the GFP-tagged retromer proteins (Vps26, Vps35 and Vps29) and RFP-tagged Snx2b in N. benthamiana cells is detected by confocal laser microscopy. The plants were infected with TBSV. Scale bars represent 10 μm. (B) Different co-localization pattern of the GFP-tagged retromer proteins (Vps26, Vps35 and Vps29) and RFP-tagged Snx2b in the absence of viral components in N. benthamiana cells is detected by confocal laser microscopy. Scale bars represent 10 μm. (TIF) [file ppat.1009120.s012.tif]

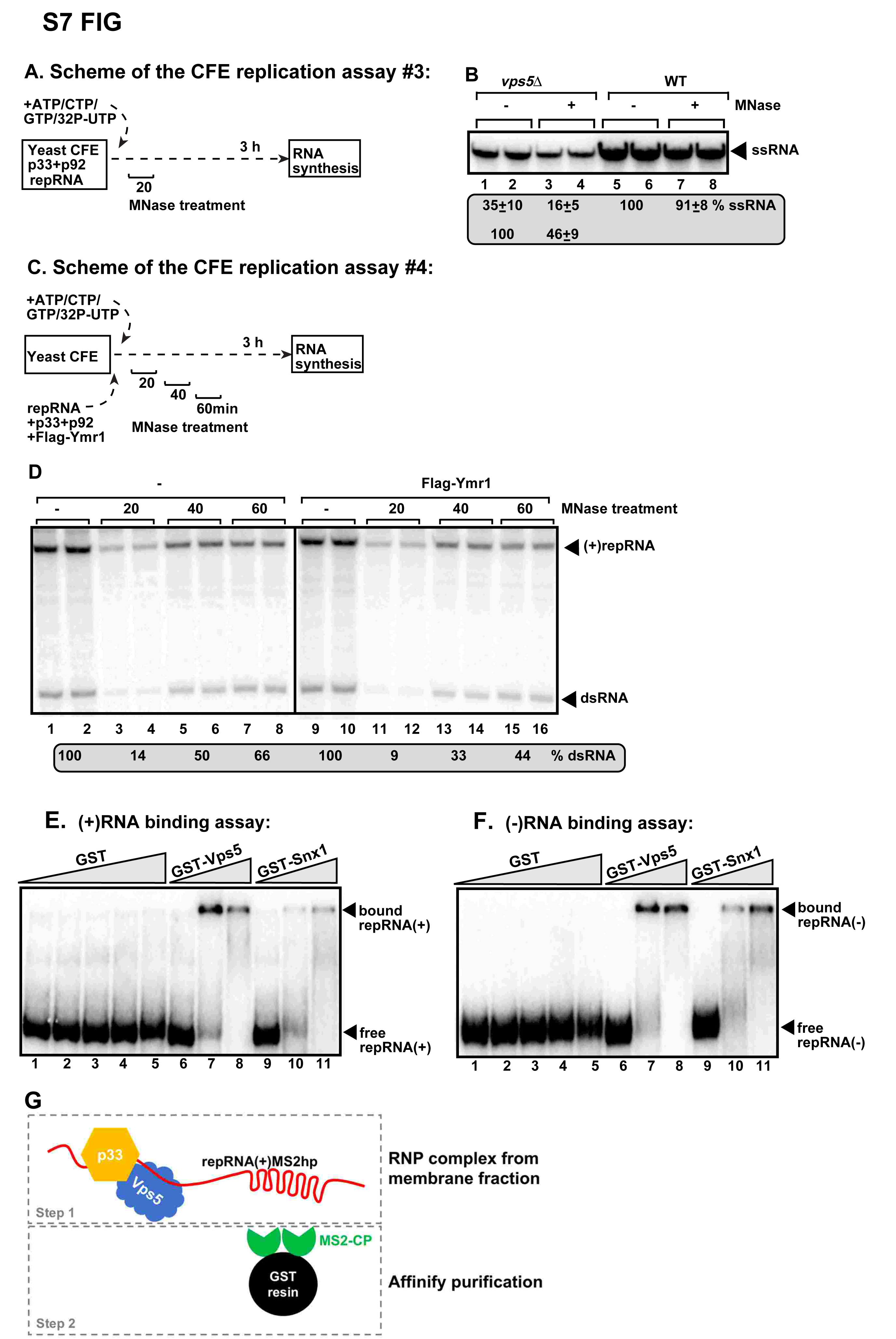

Supplement: S7 Fig — (A-B) Reduced repRNA production by the tombusvirus replicase assembled in vps5Δ yeast strain. The yeast strains expressed the p33 and p92pol replication proteins and (+)repRNA, allowing for the assembly of the viral replicase complex in yeast peroxisomal membranes. The CFEs were treated with MNase 20 min latter, followed by the inactivation of MNase after 15 min with EGTA. Non-denaturing PAGE analysis shows the 32P-labeled TBSV repRNA products from the in vitro assay. (C-D) In vitro reconstitution of the TBSV replicase using purified recombinant p33 and p92pol replication proteins and TBSV (+)repRNA. The CFEs were prepared from wt yeast and were pre-incubated with purified recombinant Flag-tagged Ymr1 PI(3)P phosphatase to reduce the PI(3)P level in the CFE. The MNase treatments, which lasted for 15 min, were done at three different time points as shown. Non-denaturing PAGE analysis was done as in Fig 5. (E-F) In vitro association of Vps5 or Snx1 SNX-BAR proteins with the viral RNAs. In vitro RNA gel mobility shift assay shows that GST-Vps5 or GST-Snx1 bind to the 32P-labeled (+)repRNA and (-)repRNA, respectively. Purified GST-Vps5, GST-Snx1 or GST was added in increasing amounts (1, 3, 6, 15 and 30 pMol for GST, 1, 3 and 6 pmol for GST-Vps5, and 6, 15 and 30 pmol for GST-Snx1) to the assays. The Vps5-repRNA or Snx1-repRNA complex was analyzed on non-denaturing 5% polyacrylamide gels. Each experiment was repeated. (G) Scheme of the MS2-CP-based RNA purification assay. Flag-p33, Flag-p92 and Vps5-3xHA and the repRNA derivatives were expressed in yeast to allow the formation of RNA-protein complexes. This scheme is to explain the experiments presented in Fig 7. (TIF) [file ppat.1009120.s013.tif]
